# Gradient matching accelerates mixed-effects inference for biochemical networks

**DOI:** 10.1093/bioinformatics/btaf154

**Published:** 2025-04-08

**Authors:** Yulan B van Oppen, Andreas Milias-Argeitis

**Affiliations:** Groningen Biomolecular Sciences and Biotechnology Institute, University of Groningen, Groningen 9747 AG, The Netherlands; Groningen Biomolecular Sciences and Biotechnology Institute, University of Groningen, Groningen 9747 AG, The Netherlands

## Abstract

**Motivation:**

Single-cell time series data often exhibit significant variability within an isogenic cell population. When modeling intracellular processes, it is therefore more appropriate to infer parameter distributions that reflect this variability, rather than fitting the population average to obtain a single point estimate. The Global Two-Stage (GTS) approach for nonlinear mixed-effects (NLME) models is a simple and modular method commonly used for this purpose. However, this method is computationally intensive due to its repeated use of nonconvex optimization and numerical integration of the underlying system.

**Results:**

We propose the Gradient Matching GTS (GMGTS) method as an efficient alternative to GTS. Gradient matching offers an integration-free approach to parameter estimation that is particularly powerful for systems that are linear in the unknown parameters, such as biochemical networks modeled by mass action kinetics. By incorporating gradient matching into the GTS framework, we expand its capabilities through uncertainty propagation calculations and an iterative estimation scheme for partially observed systems. Comparisons between GMGTS and GTS across various inference setups show that our method significantly reduces computational demands, facilitating the application of complex NLME models in systems biology.

**Availability and implementation:**

A Matlab implementation of GMGTS is provided at https://github.com/yulanvanoppen/GMGTS (DOI: http://doi.org/10.5281/zenodo.14884457).

## 1 Introduction

The development of experimental methods capable of tracking individual cells in time (Locke and Elowitz 2009, Muzzey and Van Oudenaarden 2009, Spiller *et al.* 2010) has revealed extensive variability in cellular behaviors within isogenic cell populations (Battich *et al.* 2015, Foreman and Wollman 2020, Levien *et al.* 2021, Pinheiro *et al.* 2022). Variability within a biochemical system can arise from the inherent stochasticity of chemical reactions that involve small numbers of molecules (Paulsson 2005, Raser and O’shea 2005, Raj and Van Oudenaarden 2008). On the other hand, variability extrinsic to the system of interest (e.g. cell-to-cell differences in growth rate, cell cycle stage, biosynthetic capacity, the cellular microenvironment, epigenetic states, or the abundance of species not explicitly accounted for) can also affect the observed dynamics (Huang 2009, Spencer *et al.* 2009, Zopf *et al.* 2013, Guantes *et al.* 2015, Keren *et al.* 2015, Sherman *et al.* 2015, Kramer *et al.* 2022). When a biochemical system involves highly abundant species that interact frequently, intrinsic stochasticity is typically ignored and the system is modeled deterministically via nonlinear ordinary differential equations (ODEs) (Aldridge *et al.* 2006, Albeck *et al.* 2008, Llamosi *et al.* 2016). While individual cells may behave deterministically, extrinsic sources of variability may still produce heterogeneity at the population level. When these extrinsic sources vary slowly compared to the system dynamics, the ODE-based individual model can be augmented with randomly distributed parameters and/or initial conditions to model the collective dynamics of a heterogeneous cell population (Albeck *et al.* 2008, Spencer *et al.* 2009, Waldherr *et al.* 2009, Almquist *et al.* 2015, Loos and Hasenauer 2019), giving rise to a *nonlinear mixed-effects* (NLME) dynamical system.

A number of inference methods for NLME models exist in the pharmacometric literature (Davidian and Giltinan 2003, Kuhn and Lavielle 2005), and have recently been applied to biochemical network inference (Llamosi *et al.* 2016, Dharmarajan *et al.* 2019). Stochastic Approximation Expectation Maximization (SAEM) (Kuhn and Lavielle 2005) is considered the gold standard for NLME inference. As it directly maximizes the likelihood of population-level parameters, SAEM is capable of handling complex nonlinear models with sparsely sampled individual-level data. On the other hand, it is computationally intensive, especially for larger models, as it involves repeated sampling and numerical optimization. A simpler and modular alternative is the Global Two-Stage (GTS) (Davidian and Giltinan 2017, Dharmarajan *et al.* 2019) method, which simplifies the inference problem by decoupling the estimation of individual and population-level parameters. This approach is applicable when a fair number of individual trajectories are available and each trajectory is sampled sufficiently densely to reveal the underlying system dynamics. When both SAEM and GTS are applicable, GTS has been shown to display similar accuracy while being consistently faster (Dharmarajan *et al.* 2019).

The GTS method begins by calculating cell-specific parameter estimates in the first stage and combines these estimates (and their associated uncertainties) to infer the population parameters via Expectation Maximization in the second stage. The second GTS stage improves upon the “naïve” estimator that directly computes statistics of the population distribution from the individual estimates, leading to inflated covariance estimates (Llamosi *et al.* 2016). Although GTS is highly reliable when individual cells are practically identifiable, the standard implementation of the first stage requires nonconvex optimization to estimate individual parameters by minimizing the discrepancy between the measurements and the ODE solutions. This approach, which we will call *trajectory matching*, requires repeated numerical integration of the underlying ODE system, while the optimization algorithm needs to be initialized at multiple points to avoid local optima. For these reasons, GTS quickly becomes computationally intensive as both the size of the underlying ODE system and the number of measured cells increase.

To avoid numerical integration of the system ODEs during parameter inference, an alternative class of methods, called *gradient matching* has been developed (Macdonald and Husmeier 2015). Instead of minimizing the discrepancy between measurements and ODE predictions, gradient matching minimizes the discrepancy between time derivatives predicted by the ODEs and those computed by smoothing the measurements. In this way, these methods can be thought of as minimizing the difference between the two sides of the system ODEs. Several variants of gradient matching have been proposed over time (Varah 1982, Ramsay *et al.* 2007, Calderhead *et al.* 2009, Dondelinger *et al.* 2013), with Varah (1982) being one of the first systematic treatments of the method.

Although gradient matching could greatly accelerate the first stage of GTS, it has not yet been used for the inference of NLME dynamical systems. We therefore developed the *Gradient Matching Global Two-Stage* (GMGTS) method, which constitutes the first implementation of gradient matching within the GTS framework. Besides providing point estimates of individual cell parameters, the first stage of GMGTS computes all the necessary uncertainty estimates for these parameters, which are subsequently fed into the second stage. GMGTS is particularly powerful when the ODE right-hand side is linear in the unknown parameters, as is the case for nonlinear models based on mass-action kinetics. For such systems, parameter estimation via gradient matching turns into a generalized least-squares problem that can be solved analytically via regression methods. Moving beyond the original gradient matching framework that requires full state information (Varah 1982), GMGTS is also applicable to partially observed systems thanks to an iterative estimation scheme that retains computational efficiency compared to trajectory matching.

To evaluate the applicability, efficiency, and accuracy of the GMGTS method, we carried out a set of tests using *in silico* data in different settings and for different examples of biochemical systems. We show that the computational cost of GMGTS increases much more gradually with the number of free parameters compared to GTS. Moreover, GMGTS is considerably more economical than GTS while providing sufficiently accurate estimates of population parameters within a realistic range of time step sizes and measurement noise levels. Since gradient matching benefits greatly when the underlying system is linear in parameters, we also demonstrate how certain biochemical systems with nonlinear parameter dependence can be reformulated to satisfy this requirement. Having validated the GMGTS method in a simulation-based environment, we utilize it to infer parameter variability from single-cell data for a system describing fluorescent protein maturation in budding yeast (Guerra *et al.* 2022). Our results for a set of twelve commonly used fluorescent proteins suggest that maturation rates of fluorescent proteins display considerable variability across an isogenic yeast population, reflecting recent findings in mammalian cells (Wu *et al.* 2020).

Overall, GMGTS combines the strengths of gradient matching and the GTS method to provide sufficiently accurate population parameter estimates at a fraction of the time required by GTS. In this way, GMGTS increases the applicability of NLME parameter inference to computationally demanding problems comprising a large number of single-cell measurements and complex dynamical systems. As the development of advanced experimental methods increases the amount and complexity of single-cell data, we expect that NLME dynamical systems will become a “standard element” of the systems biology modeling toolbox in the future, similar to what ODE models have been in the past. We therefore expect that our work will further stimulate and facilitate the use of this powerful class of models in systems biology applications.

## 2 Systems and methods

### 2.1 Nonlinear mixed-effects dynamical models and the inference problem

To capture the dynamic behavior of a heterogeneous cell population, we use a *nonlinear mixed-effects* (NLME) model. In this model, a set of ordinary differential equations (ODEs) describes the dynamics of the biochemical network of interest, while cell-to-cell variability is introduced by assuming that each cell “samples” a particular set of kinetic parameters from a multivariate probability distribution. More concretely, the dynamics of the *i*th cell in a population of *N* cells is described by a *K*-dimensional ODE system (the *individual model*) of the form

(1) $${\dot{x}}_{i}(t)=f(x_{i}(t);\beta_{i})\;\text{for}\;t⩾0,$$

with (known) initial condition $x_{i}(0)=x_{i}^{0}$ (an examination of this assumption and its implications is provided in the Section 4). We further assume that the cell-specific vector of unknown kinetic parameters $\beta_{i}$ is drawn from a multivariate normal distribution with mean **b** and covariance **D**; i.e. $\beta_{i}∼N(b,D)$ where **b** is a vector of *fixed effects* and **D** is the *random effects* covariance matrix. Alternatively, one can assume that $\beta_{i}$ follows a log-normal distribution, i.e. $\beta_{i}∼\text{LN}(b,D)$, where **b** and **D** denote the log-mean and log-covariance respectively.

In the following, we consider the case in which the right-hand side of the individual model is linear in the unknown parameters; i.e. when the right-hand side of (1) can be decomposed as

(2) $${\dot{x}}_{i}(t)=g(x_{i}(t))\;\beta_{i}+h(x_{i}(t))\;\text{for}\;t⩾0,$$

where g(·) is a matrix-valued function multiplying the parameter vector $\beta_{i},\;h(\cdot )$ is a vector-valued function, and both functions are independent of $\beta_{i}$. As we will detail below, parameter estimation via gradient matching is particularly efficient with models of the form (2) since it can be carried out via (generalized) least squares (GLS) methods (Varah 1982). Linearity in parameters arises naturally in models based on mass action kinetics and, more generally, when the right-hand side of (1) is a polynomial function of **x** with individual elements of $\beta_{i}$ as coefficients. As we will demonstrate below, an approximate inference problem can be solved for systems with Michaelis-Menten or Hill kinetics after turning these systems into a form that is linear in parameters. This can be done by writing elementary (mass-action) equations from which the Michaelis-Menten or Hill approximations were derived.

We assume that each cell is observed at a discrete set of time points $t_{1},\ldots ,t_{T}$. The number and locations of the observation points may differ among cells, but we will assume that they are the same to simplify the notation without loss of generality. The experimental observations of the *i*th cell are then related to the solution $x_{i}^{*}(t)$ of (1) given $\beta_{i}$ through the measurement model

(3) $$y_{i}(t)=Q\;x_{i}^{*}(t)+\varepsilon_{i}(t)\;\text{for}\;t=t_{1},\ldots ,t_{T}˙$$

**Q** is a binary matrix that is used to select the measured components of $x_{i}^{*}(t)$. For instance, when all states are observed, **Q** is equal to the *K*-dimensional identity matrix. For more general forms of measurement functions, see Supplementary Section S2.1. We further assume that the measurement errors $\varepsilon_{i}(t)$ are a combination of uncorrelated additive and multiplicative zero-mean Gaussian noise. Therefore, the *k*th component of $\varepsilon_{i}(t_{j})$, denoted $\varepsilon_{ik}(t_{j})$, has mean 0 and variance $\sigma_{k}^{2}+\tau_{k}^{2}{\left[x_{ik}^{*}(t_{j})\right]}^{2}$. In this expression, *σ_k_* and *τ_k_* are positive constants (the same for all cells), and $x_{ik}^{*}(t_{j})$ denotes the *k*th component of the solution $x_{i}^{*}(t)$ at time *t_j_*.

Given the mixed-effects model presented in (2) and (3), our goal is to infer the *population parameters* **b** and **D** that give rise to the observed variability in single-cell trajectories. To achieve this, the GMGTS method builds upon the GTS framework. The latter is presented in Davidian and Giltinan (2017) and summarized in Supplementary Section S2.2.

### 2.2 GMGTS with full state information

To introduce the key concepts of GMGTS, we begin by considering the case with full state information. While we assume that **Q** is a *K*-dimensional identity matrix, the case of a general invertible **Q** can be handled in a similar manner, with minor modifications to the smoothing step. GMGTS with full state information proceeds in two stages:

- For the *i*th cell, an individual kinetic parameter estimate ${\hat{\beta }}_{i}$ and its associated uncertainty (covariance matrix $C_{i}$) are obtained through gradient matching using linear regression; see (4) below.
- For normally distributed individual parameters, the population parameters **b** and **D** are inferred from the individual estimates $\left\{{\hat{\beta }}_{i},\;i=1,\ldots ,N\right\}$ and their uncertainties via an Expectation-Maximization scheme (Supplementary Section S4) identical to the second stage of the GTS method. For log-normally distributed parameters, we present an approximate inference method in Supplementary Section S5.

To estimate individual parameters in Stage I, the time evolution and time derivatives of the measured data need to be approximated. Therefore, for the *i*th cell, we compute a B-spline smoothing estimate ${\hat{x}}_{i}(t)$ of $y_{i}(t_{1}),\ldots ,y_{i}(t_{T})$, which is differentiated to obtain gradient estimates ${\dot{\hat{x}}}_{i}(t)$. The measurement noise parameters *σ_k_* and *τ_k_* are also estimated during the smoothing step (cf Supplementary Section S6 for the technical details).

Starting from the state and gradient estimates ${\hat{x}}_{i}(t)$ and ${\dot{\hat{x}}}_{i}(t)$, we use (2) to define a linear model

(4) $$\left[\begin{array}{c} {\dot{\hat{x}}}_{i}(t_{1})\\ \vdots \\ {\dot{\hat{x}}}_{i}(t_{T}) \end{array}\right]-\left[\begin{array}{c} h({\hat{x}}_{i}(t_{1}))\\ \vdots \\ h({\hat{x}}_{i}(t_{T})) \end{array}\right]=\left[\begin{array}{c} g({\hat{x}}_{i}(t_{1}))\\ \vdots \\ g({\hat{x}}_{i}(t_{T})) \end{array}\right]\beta_{i}+\Delta_{i},$$

where we assume that (*TK*)-dimensional vector of residuals $\Delta_{i}$ follows a multivariate normal distribution (Supplementary Section S2.3). In this model, the unknown parameter vector ${\hat{\beta }}_{i}$ can be estimated using linear regression methods. However, since the uncertainty of the measurements $y_{i}(t)$ propagates through the state and gradient estimates (${\hat{x}}_{i}(t)$ and ${\dot{\hat{x}}}_{i}(t)$) to both sides of (4), the covariance matrix $\text{Var}\;\Delta_{i}$ depends on the current estimate of $\beta_{i}$ and needs to be estimated iteratively within a Feasible Generalized Least Squares (FGLS) scheme (Greene 2003). This structure of the linear regression problem also complicates the approximation of $C_{i}$, which needs to be carried out using the delta method (Asmussen and Glynn 2007). In brief, Stage I of GMGTS consists of the following steps for the *i*th cell:

I.1 **Initialization.** Obtain estimates ${\hat{x}}_{i}(t)$ and ${\dot{\hat{x}}}_{i}(t)$ of the observed states and their gradients from B-spline smoothing of the observations $y_{i}(t_{1}),\ldots ,y_{i}(t_{T})$. Initialize $\text{Var}\;\Delta_{i}$ as a *TK*-dimensional identity matrix (cf SI, Remark S3).
I.2 **Gradient matching.** Using the current approximation of $\text{Var}\;\Delta_{i}$, obtain the generalized least squares (GLS) estimate of ${\hat{\beta }}_{i}$ from (4) (cf Supplementary Section S2.3).
I.3 **GLS residual covariance.** Approximate the covariance matrix $\text{Var }\Delta_{i}$ of the residuals of the next GLS iteration (Supplementary Section S7). Steps 2 and 3 constitute an FGLS iteration and are repeated until convergence.
I.4 Approximate the covariance matrix $C_{i}$ of ${\hat{\beta }}_{i}$ (Supplementary Section S7).

**Figure 1.**
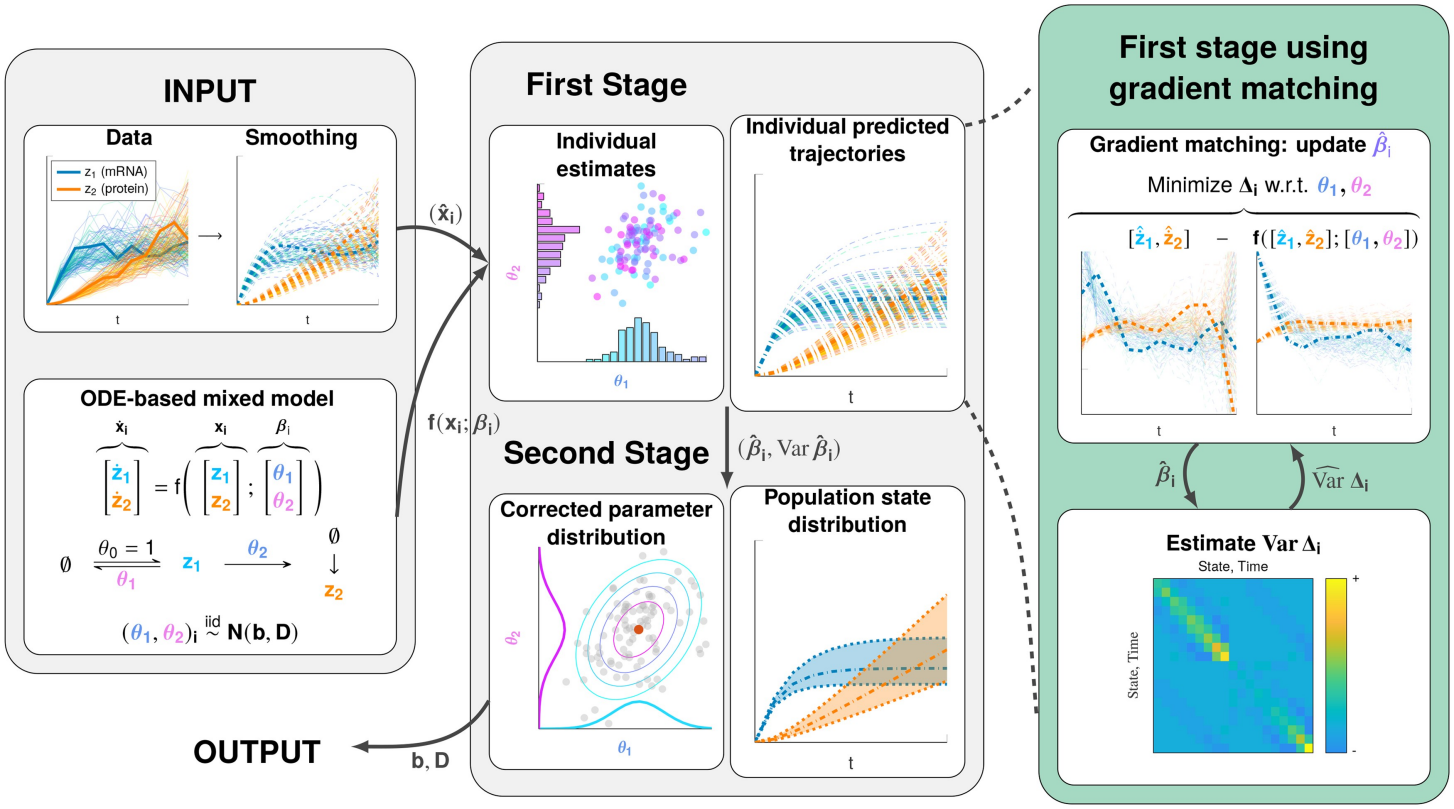
Graphical overview of the GMGTS method for fully observed systems. During initialization, the data are smoothed using penalized cubic B-splines (*left panel, top*) and are provided as input to the method along with an ODE-based model with randomly distributed parameters (*left panel, bottom*). The smoothed trajectories ${\hat{x}}_{i}$ are used in the first stage (*middle panel, top*) to obtain individual parameter estimates ${\hat{\beta }}_{i}$ through gradient matching (*right panel, top*). Because of the linearity in parameters of the ODE system, gradient matching is carried out by solving a generalized least squares regression problem. This regression problem has a vector of residuals $\Delta_{i}$ with an unknown covariance matrix, which needs to be iteratively estimated (*right panel, bottom*). Upon convergence, uncertainties of the individual parameter estimates ($\text{Var}\;{\hat{\beta }}_{i}$) are computed by propagating the uncertainty from the single-cell data through the spline estimates. The parameter estimates ${\hat{\beta }}_{i}$ and their corresponding uncertainties are used in the second stage (*middle panel, bottom*), where the population parameter estimates $\hat{b},\hat{D}$ are obtained. The box corresponding to the first stage of GMGTS is highlighted.

Figure 1 shows a graphical overview of the GMGTS approach. Supplementary Section S2.3 provides the mathematical details of GMGTS and Supplementary Section S7 provides uncertainty-related calculations. Implementation details of GTS and GMGTS are provided in Supplementary Section S11. Matlab implementations and documentation can be found at https://github.com/yulanvanoppen/GMGTS. To facilitate the smoothing step of GMGTS, our implementation contains an interactive smoothing app. An automatic tuning heuristic based on the detection of salient time series features (described in Supplementary Section S11.1) provides initial smoothing estimates which can be further adjusted by the user.

### 2.3 GMGTS with partial state information

By construction, the original gradient matching method (Varah 1982) requires measurements of the entire state vector to evaluate the model gradients. However, it is not always feasible to observe all states of a biochemical system. For these cases, we extend the estimation procedure in the first GMGTS stage with another iterative scheme. In this scheme, gradient matching and residual covariance estimation are alternated with an additional step in which we numerically integrate the individual model (2) to obtain estimates of the hidden states and their gradients. Although ODE integrations are not entirely avoided in this case, their number is negligible compared to those required for trajectory matching, as we will show below.

As with full state information, to compute the GLS estimate of individual parameters at the gradient matching step, we need an estimate of the residual covariance matrix $\text{Var}\;\Delta_{i}$. The approximation of this matrix becomes significantly more elaborate in the partially observed case since uncertainty in the measurements also needs to be propagated via the hidden states estimates (see Supplementary Section S8). For the same reason, approximating the uncertainty $C_{i}$ of the parameter estimate ${\hat{\beta }}_{i}$ is now an integral part of these uncertainty calculations, whose details are presented in Supplementary Section S8.

Concretely, in the modified first stage (I) GMGTS for partially observed systems, we perform the following operations for the *i*th cell:

I.1* **Initialization.** Smooth the observed data $y_{i}(t)$, integrate (2) with the initial guess ${\hat{\beta }}^{0}$ (Supplementary Section S2.4) to obtain estimates of hidden states and their gradients, and compute an initial estimate of $\text{Var}\;\Delta_{i}$ (Supplementary Section S8).
I.2* **Gradient matching.** Obtain the parameter vector estimate ${\hat{\beta }}_{i}$ using (4).
I.3* **Estimation of hidden states.** Update the hidden state and gradient estimates by integrating (2) using ${\hat{\beta }}_{i}$.
I.4* **GLS residual covariance.** Estimate the uncertainty $C_{i}$ of ${\hat{\beta }}_{i}$ (Supplementary Section S8) and use it to propagate the uncertainty from the observed and hidden state and gradient estimates to $\text{Var}\;\Delta_{i}$ for the next GLS iteration. Repeat steps 2–4 until convergence.

**Figure 2.**
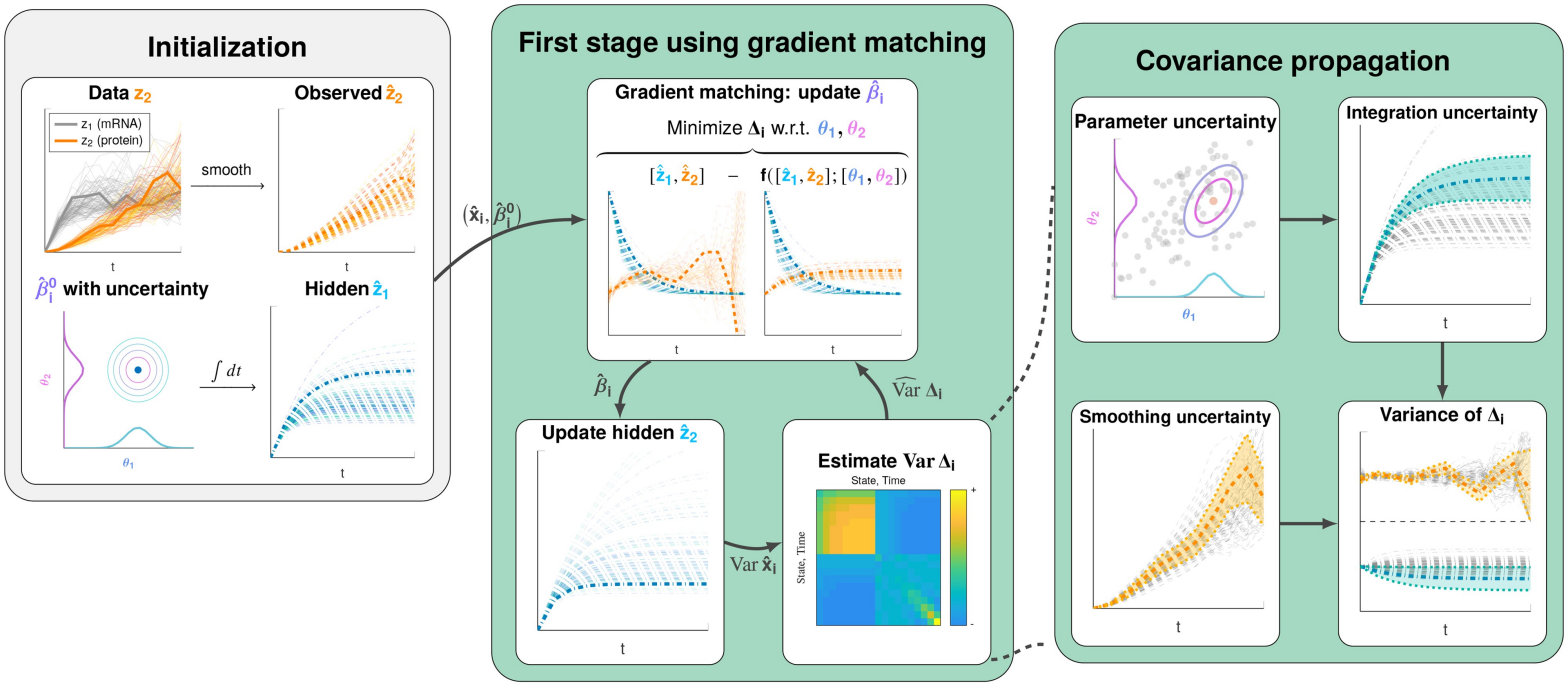
Graphical overview of the GMGTS method modifications to the first stage for partially observed systems. Besides smoothing the measured states, an initial parameter guess ${\hat{\beta }}^{\text{init}}$ is required to initialize the hidden state estimates by numerically integrating the ODE system (*left panel*). With full state information now available, an extended iterative scheme comprises the modified first stage of GMGTS. In this scheme, an individual parameter estimate ${\hat{\beta }}_{i}$ is obtained via gradient matching using generalized linear regression (*middle panel, top*). Numerical integration of the ODE system yields updated hidden state estimates (*middle panel, bottom left*), whose uncertainty needs to be considered together with the smoothing uncertainty to approximate the residual covariance matrix $\text{Var}\;\Delta_{i}$ (*middle panel, bottom right*). The smoothing uncertainty affects the residual covariances directly but also propagates through the updated parameter estimates to the hidden states via the numerical ODE solutions (*right panel*). The boxes corresponding to the first stage of GMGTS are highlighted.

Figure 2 provides a graphical overview of the modified first-stage estimation procedure for partially observed systems. Steps 2–4 alternate between gradient matching and hidden state estimation to reconstruct the missing state information. As we detail in Supplementary Section S10, this process can be interpreted as a fixed-point iteration. In most of our tests, five or ten iterations suffice to achieve convergence for >90% of the individual parameter estimates. With each iteration involving a small number of numerical ODE integrations (cf Supplementary Section S11.3), the total number of integrations is significantly reduced compared to the hundreds of integrations required by trajectory matching for a single optimization starting point. The mathematical details of this scheme are provided in Supplementary Section S2.4, and implementation details are provided in Supplementary Section S11.

## 3 Results

### 3.1 GMGTS achieves good accuracy across a range of sampling intervals and noise levels

Given that GMGTS relies on time series smoothing, we first tested how the accuracy of the estimated random effects distribution depends on measurement noise levels and the number of measurement time points. For these tests, we used two example systems under different scenarios of data sparsity and noisiness.

The first example is a model of a bifunctional two-component signaling cascade (Kurdyaeva and Milias-Argeitis 2021) comprising a histidine kinase *H* and a response regulator *R*. In response to an external stimulus, the histidine kinase autophosphorylates (*H_p_*) and transfers its phosphate group to the response regulator (*R_p_*). At the same time, the nonphosphorylated form of the bifunctional kinase (*H*) acts as a phosphatase toward *R_p_*. The system comprises six nonlinear ODEs with polynomial right-hand sides that are linear in parameters. Full model details are provided in Supplementary Section S3.2. Besides the fully observed case, we also treated the case where the observed variables are the total phosphorylated kinase (Duvall and Childers 2020) and response regulator (Butcher and Tabor 2022), along with total unbound kinase and response regulator. To avoid loss of structural identifiability, we fixed one rate constant in each pair of reverse reactions ($k_{1}=k_{2}=k_{6}=0.5$ and $k_{4}=1.5$), and let $k_{3},k_{5},k_{7},k_{8}$ vary across a population according to normal distributions with means 0.5,0.1,0.8,0.2 and a common coefficient of variation equal to 0.25.

The second example is a model of fluorescent protein synthesis and maturation in budding yeast (Guerra *et al.* 2022). In this system, transcription of the protein is activated in a step-wise manner at *t *=* *0, immature (“dark”) protein (*D*) is translated from mRNA (*M*), and matures into fluorescent protein (*F*). The dynamics of this system is described by a system of delay differential equations provided in Supplementary Section S3.3. We considered both fully and partially observed cases, with only measurements of *F* being available in the latter. To avoid loss of structural identifiability (Guerra *et al.* 2022), the dilution rate was fixed to $k_{\text{dil}}=0.004\;{\text{min}}^{-1}$ and the mRNA synthesis rate was set to $k_{r}=0.1$ transcripts per minute. Since our tests indicated that $k_{\text{dr}}$ is weakly identifiable even when it has a common value in all cells, we assumed that it is fixed at a $k_{\text{dr}}=0.07\;{\text{min}}^{-1}$. This value corresponds to an mRNA half-life of ∼10 min, which is in line with previous estimates for budding yeast (Neymotin *et al.* 2016). We further assumed that $k_{p}$ and $k_{m}$ follow normal distributions across a cell population with respective means 0.025 and 0.05, and with coefficients of variation equal to 0.25.

**Figure 3.**
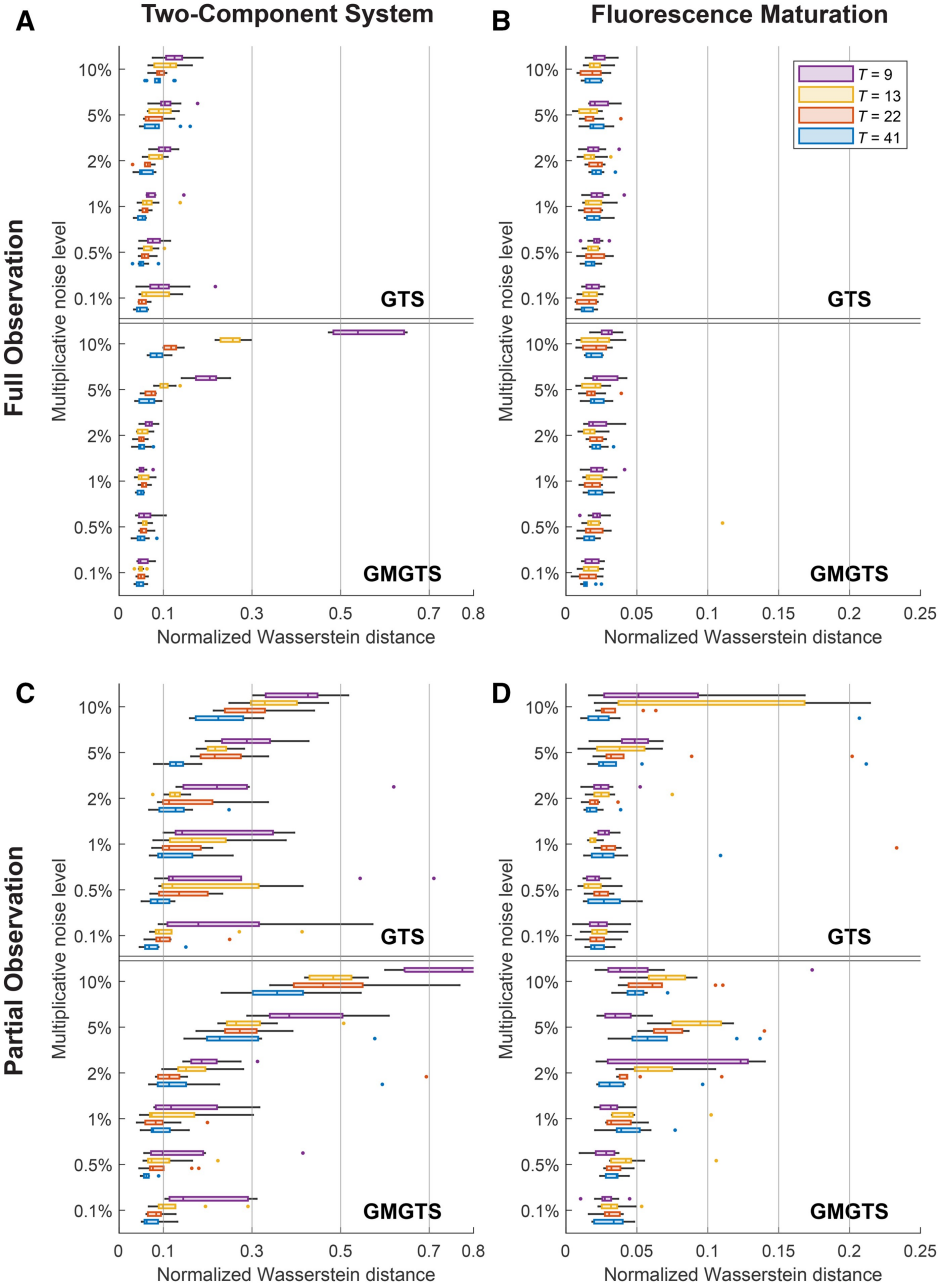
Accuracy comparison between GTS and GMGTS for two-component and FP maturation systems. The multiplicative noise level (*τ*, *y*-axis) and the number of simulated measurement time points (*T*, color labels) were varied to test the accuracy of each method. For each setup, 10 independent sets of individual parameters and corresponding measurements were sampled and used for inference of the random effects distributions. The boxplots summarize the mismatch between the inferred and the data-generating random effects distributions in terms of normalized Wasserstein distances. Points lying further than 1.5 times the interquartile range of each box are considered outliers and are displayed as dots. (A and C) Results for the bifunctional two-component signaling cascade. (B and D) Results for the one-step fluorescent protein expression and maturation model. For all but the noisiest and sparsest measurements, the additional accuracy loss of GMGTS is small compared to that of GTS.

**Figure 4.**
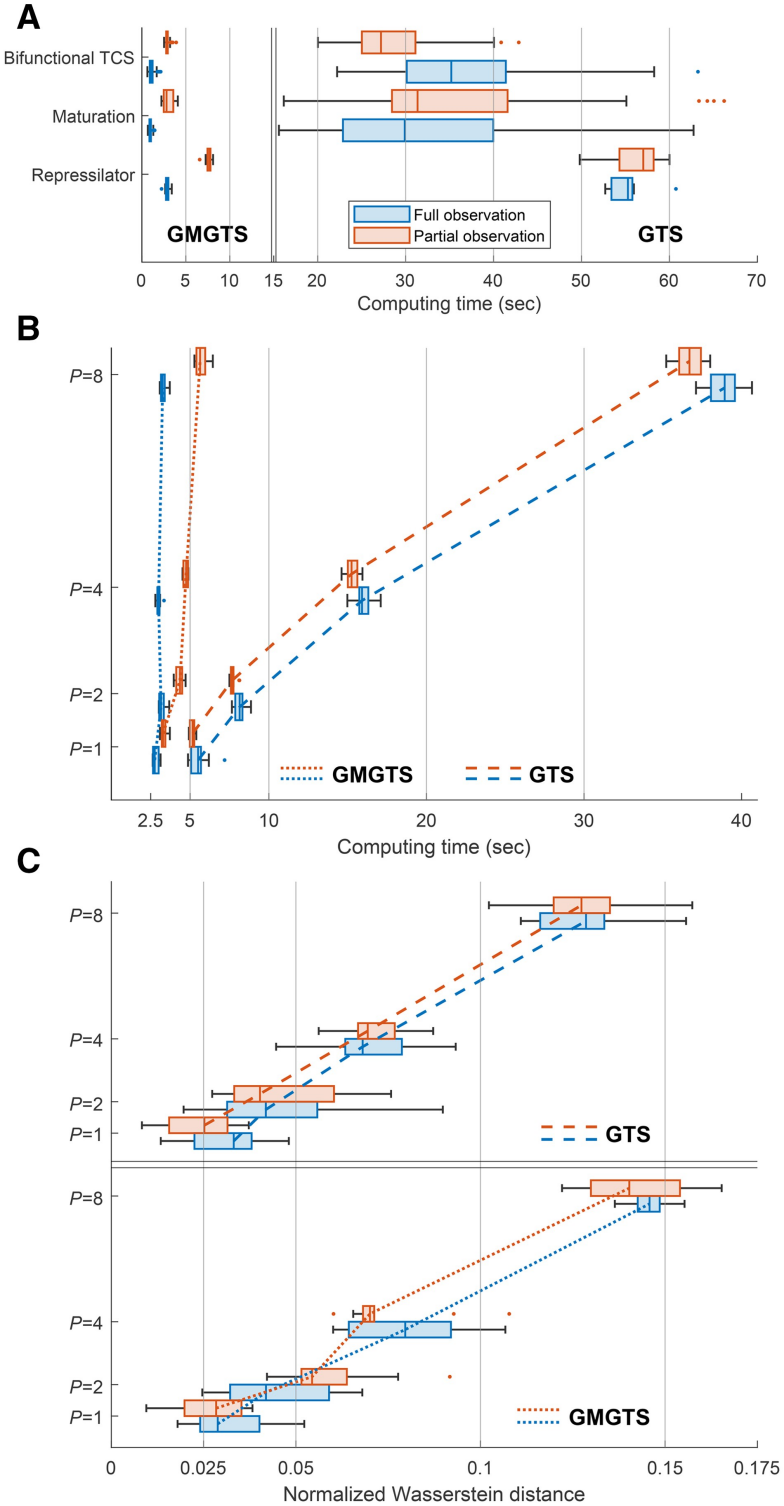
Computational efficiency comparison between GMGTS and GTS in different simulation studies. Computing times and inaccuracies of the inferred population distributions were obtained using simulated data as ground truth, and were summarized using boxplots. For each test setup, both GMGTS and GTS were applied to fully and partially observed systems. The B-spline knots were kept the same across all cells and test settings (for details, see Supplementary Section S11.1). Points lying further than 1.5 times the interquartile range of each box are displayed as dots. (A) Summary of computing times for multiple data generation and inference runs for the mechanistic models presented above (240 runs for the two-component system and the maturation model, 10 for the repressilator). The GMGTS method provides a 8- to 40-fold speedup compared to GTS, both with full and partial state information. (B) Computational efficiency comparison between the two methods with respect to the number of free parameters (*P*) in a fixed Lotka-Volterra model structure. For each value of *P*, 10 independent data samples and corresponding parameter distributions were generated and inferred. In the partially observed case, the first four states were assumed to be hidden. (C) Accuracy comparison between the two methods for the simulation setup considered in Panel B.

To understand how the inferred random effects distributions depend on the quality and quantity of measurement data in GTS and GMGTS, we generated noisy measurement time series (between *t *=* *0 and t=100 min for the two-component system and between *t *=* *0 and t=200 min for the protein maturation model) while varying the number of time points *T* and the multiplicative error factor *τ*. For each combination of settings, we generated 10 independent samples of individual parameters assuming a population of *N *=* *200 measured cells in each repetition (*N *=* *500 for the FP model) and monitored how the random effects distribution was recovered by GTS and GMGTS. Figure 3 summarizes the resulting mismatch between ground truth (data-generating) and estimated distributions in terms of a normalized Wasserstein distance *W*_2_ (details provided in Supplementary Section S11.5). Overall, with the exception of overly sparse and noisy measurements, the accuracy of the GMGTS distribution estimates is comparable to that of GTS and displays good robustness to measurement noise. For a visual accuracy comparison, Supplementary Figs S7 and S8 show representative examples of the inferred random effects distributions and the predicted state distributions for the two-component system and maturation models. Although the additional loss in accuracy incurred by GMGTS is small, the method is significantly more computationally efficient than GTS, which takes 8–40 times longer for the two-component system and 10–40 times longer for the maturation model. A comparison of computing times for the two methods is provided in Fig. 4A.

### 3.2 Expanding system equations using mass action kinetics enables parameter inference via gradient matching using linear regression

When the individual ODE model is linear in the unknown parameters, the gradient matching step of GMGTS is carried out via linear regression, which is considerably faster than numerical optimization. Biochemical models derived from the law of mass action automatically fulfill this requirement. On the other hand, models with nonlinear dependence on parameters are often based on quasi-steady-state approximations of mass action kinetics. Therefore, it is sometimes possible to expand these models so that they become linear in parameters. In such cases, GMGTS can still be used to infer the population parameters in a computationally efficient manner.

To illustrate this point, we considered the mathematical model of the repressilator, a synthetic gene network that exhibits self-sustaining oscillations via delayed negative feedback (Elowitz and Leibler 2000). The system consists of three transcriptional repressors arranged in a ring topology so that each protein represses the expression of the next and is repressed by the previous one. Its dynamics is described by six coupled ODEs

(5) $$\begin{array}{c} \begin{array}{c} {\dot{m}}_{n_{1}}(t)=0.0005+\frac{0.5\alpha_{n_{1}}}{\alpha_{n_{1}}+0.00025{\left(p_{n_{2}}(t)\right)}^{2}}-0.3466m_{n_{1}}(t),\\ {\dot{p}}_{n_{1}}(t)=20m_{n_{1}}(t)-\beta_{n_{1}}p_{n_{1}}(t), \end{array}\\ \text{for}\;(n_{1},n_{2})=(1,3),(2,1),(3,2), \end{array}$$

where *m_n_* and *p_n_* denote the respective mRNA and protein concentrations (µM) of the *n*th repressor (*n *=* *1, 2, 3). For this example, we assumed that *α_n_* and *β_n_* (*n *=* *1, 2, 3) vary across a cell population according to a normal distribution with means 0.16 µM and 0.0693 min^–1^, respectively. Moreover, each parameter had a coefficient of variation equal to 0.1, and each pair $\left(\alpha_{n},\beta_{n}\right)$ had a correlation coefficient ρ=0.5. With these parameter settings, the system displays sustained oscillations in protein and mRNA concentrations across a large range of random effect values.

The model equations (5) contain Hill functions, which are nonlinear in the unknown parameters. Since these Hill functions have been derived from quasi-steady-state approximations of protein-DNA interactions, (5) can be expanded with additional states (protein-DNA complexes) to produce a system that is described by mass-action kinetics and is thus linear in parameters. The derivation of the expanded system can be found in Supplementary Section S3.1; Supplementary Fig. S1 shows that the original and expanded systems display very similar behavior, as expected. With this expansion, we naturally assumed that the additional protein-DNA complexes are hidden states.

To compare the performance of GTS and GMGTS on the inference of random effects distribution for this example, we sampled *N *=* *200 individual parameter vectors (corresponding to 100 cells) and generated measurements for each pair *m_n_* and *p_n_* assuming a 5-min sampling interval over a period of 100 min and multiplicative measurement noise (τ=5%). The whole inference process was repeated ten times with independently sampled datasets of 100 cells, each using the expanded model (Supplementary Section S3.1) for both GTS and GMGTS. Supplementary Fig. S9 shows the parameter and state distributions for the first repressor fitted by GTS and GMGTS in a representative inference run, and provides accuracy comparisons between GMGTS and GTS. Both approaches provide adequate population parameter estimates and predicted population trajectories, but the time required by GTS is 8–20 times longer compared to GMGTS (Fig. 4A).

### 3.3 The computational cost of GMGTS scales modestly with the number free parameters

Parameter estimates obtained via gradient matching may be less precise than those obtained by trajectory matching, but the former can be considerably faster when the individual model is linear in the unknown parameters. Our goal here is to explore this trade-off between GMGTS and GTS more systematically.

Figure 4A summarizes the computation times for the simulation studies presented so far. In brief, GMGTS offers a 8- to 40-fold speedup over GTS. To further understand this behavior, we tested how the computational cost and accuracy of GMGTS and GTS scale with the number of free parameters within a fixed model structure. To this end, we considered a generalized Lotka-Volterra system with 16 species (*x_n_*, n=1,…16) governed by the following set of differential equations

(6) $${\dot{x}}_{n}(t)=\{\begin{array}{cc} x_{n}(t)(r_{n}-k_{n}x_{n+1}(t)) & \;\text{for}\;n=1,\ldots ,15,\\ x_{n}(t)(r_{n}-k_{n}x_{1}(t)) & \;\text{for}\;n=16. \end{array}$$

In these equations, the *n*th birth rate *r_n_* was fixed at n/80 for all species, and a subset of the *k_n_* parameters were assumed to be variable with mean 0.02 and standard deviation 0.005. The number *m* of free parameters was fixed at m=1,2,4,8 by setting $k_{n_{1}}=k_{n_{2}}$ when $n_{1}\equiv n_{2}\;\mathrm{mod}\;m$ (e.g. $k_{1}=k_{3}=\cdots$ when *m *=* *2). We considered both fully and partially observed systems with the first four states assumed to be hidden in the latter case. For each simulation setting, we sampled *N *=* *100 vectors of individual random effects $\left(k_{1},\ldots ,k_{m}\right)$ from the random effects distribution described above, and generated the corresponding measured time series assuming multiplicative measurement noise (τ=5%) and fixed measurement time points t=0,1,2,…,20. For both GMGTS and GTS, we recorded the computation time and the normalized Wasserstein distance of the estimated random effects distribution from the ground truth. Figure 4B summarizes the computing times for ten independent runs of each method, showing that the cost of GTS scales quite steeply with the number of free parameters, while the cost of GMGTS shows only a modest increase and the accuracies of both methods are comparable.

### 3.4 Fluorescent protein maturation rates show considerable variability across budding yeast populations

The maturation rate of a fluorescent protein (FP) limits the temporal resolution at which dynamical intracellular processes can be monitored and is a major determinant of *in vivo* FP brightness in fast-dividing cells (Balleza *et al.* 2018, Guerra *et al.* 2022). For example, to monitor transient gene expression changes during the cell cycle of fast-dividing cells, FPs need to have sufficiently short maturation half-times compared to the typical cell cycle duration of those cells (Guerra *et al.* 2022). Whereas maturation rates are typically reported using population-averaged measurements, recent work uncovered significant cell-to-cell variability in FP maturation rates across isogenic mammalian cells (Wu *et al.* 2020).

Motivated by these findings, we asked to what extent cell-to-cell variability affects FP maturation kinetics in *Saccharomyces cerevisiae* (budding yeast). In our previous work (Guerra *et al.* 2022), we presented a systematic *in vivo* characterization of maturation for a set of twelve FPs that are commonly used in this organism. Our experimental data comprised single-cell time course measurements of FP accumulation upon step-wise induction of protein expression with an optogenetic system. FP maturation rates were then inferred by fitting a mathematical model to the *averages* of the single-cell traces. While the use of averaged data allowed us to align and compare our maturation rate estimates with previous work, the datasets of (Guerra *et al.* 2022) contained rich single-cell information (∼50 to 100 cells per experiment) which could provide insights into the variability of maturation rates across cell populations. We therefore applied GMGTS to infer ODE-based mixed-effects models of FP expression and maturation for the same set of twelve FPs.

When FP expression is under the control of a strong promoter, ODE models have proven to be a suitable approximation for capturing single-cell FP dynamics (Llamosi *et al.* 2016, Gligorovski *et al.* 2023). To our knowledge, FP maturation has not been explicitly modeled at the single-cell level, but there is little reason to expect significant intrinsic variability in this process. Fluorophore maturation involves one or two rate-limiting steps, meaning that individual FP molecules mature with waiting times following an exponential or gamma distribution. However, a strong promoter such as the one used in our previous work (Guerra *et al.* 2022), drives the production of thousands of immature FP molecules. This large number ensures that the randomness of individual waiting times averages out, allowing the maturation process to be modeled deterministically.

**Figure 5.**
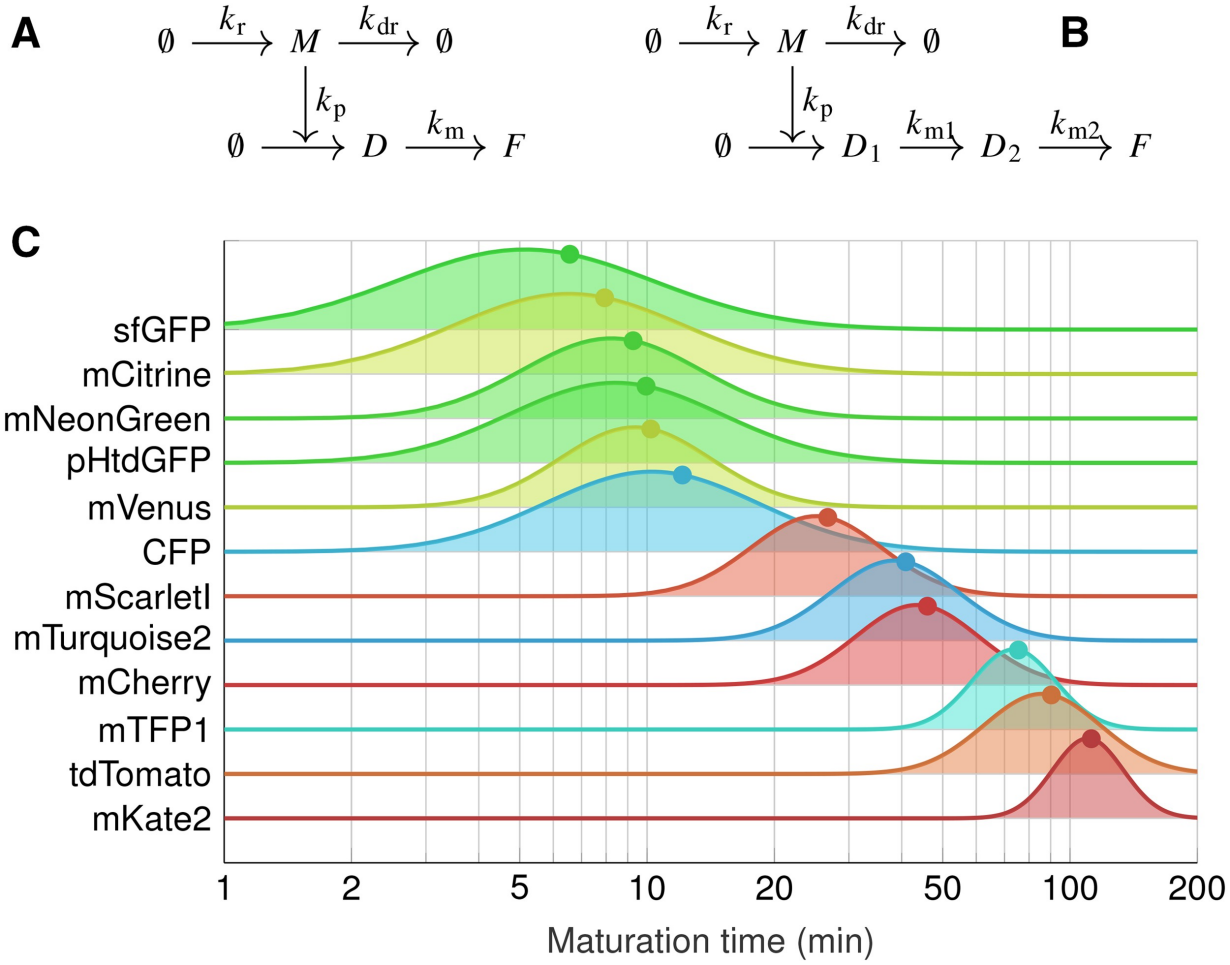
Schematic representation of the mathematical models describing FP maturation, and inferred distributions of maturation half-times. (A and B) Schematics of the one-step (left) and two-step (right) models. Dark protein (*D* or *D*_1_) is synthesized from mRNA (*M*) and matures into *F* (via *D*_2_ in the two-step model). Each state is diluted at a common rate due to cell growth (omitted here for clarity). (C) Estimated maturation half-time distributions across the different tested FPs. Half-time distributions were computed from the corresponding distributions of maturation rate estimates (Supplementary Section S3.6), and are ordered based on the half-times obtained from their average maturation rate (indicated with dots). For two-step proteins, an approximate maturation half-time was derived analytically (Supplementary Section S3.5). Single-step maturing FPs were estimated to be considerably faster, and their maturation rates displayed larger variability compared to proteins undergoing two-step maturation (cf Supplementary Table S2).

Similarly to Guerra *et al.* (2022), we considered models with one- and two-step FP maturation kinetics, to account for proteins with one or two rate-limiting maturation steps. In the one-step model, *D* matures into mature fluorescent protein (*F*) via a single rate-limiting step with rate $k_{m}$. In the two-step model, *D*_1_ first transitions into an intermediate (nonfluorescent) form (*D*_2_) at rate $k_{m1}$ before maturing into *F* at rate $k_{m2}$. $k_{m1}$ and $k_{m2}$ cannot be inferred individually given only fluorescence data, but replacing them with a single parameter (i.e. assuming $k_{m}:=k_{m1}=k_{m2}$) introduces negligible error when the two rates are of the same order of magnitude (Guerra *et al.* 2022). Figure 5A and B contain reaction schematics for the two models and the delay differential equations describing model dynamics can be found in Supplementary Section S3.3.

Given that only the concentration of *F* can be measured by fluorescence microscopy, we assumed that states *M* and *D* (*D*_1_ and *D*_2_ in the two-step model) are not observed. To ensure structural identifiability of the models, the average dilution rate due to cell growth was estimated by tracking cell volume measurements over time (Guerra *et al.* 2022), and was fixed to an experiment-specific value for each protein ($k_{\text{dil}}\approx 0.0045\;{\text{min}}^{-1}$). We assumed a common dilution rate for all cells in a given experiment due to practical constraints imposed by the single-cell data (Supplementary Section S13.1). However, as discussed in Supplementary Section S13.1, disregarding dilution rate variability during inference does not substantially increase the variability of maturation rate estimates, provided that the variability in maturation rates is significantly greater than the experimentally observed variability in dilution rates.

Given the fact that the product $k_{r}k_{p}$ is identifiable but the individual synthesis rates are not (Guerra *et al.* 2022), we fixed $k_{r}$ and let $k_{p}$ be variable. Finally, given the poor practical identifiability of $k_{\text{dr}}$ (cf simulation study above), we fixed it at $0.07\;{\text{min}}^{-1}$, which is within the range of previously obtained mRNA degradation rate estimates for GFP in budding yeast (Neymotin *et al.* 2016). With these choices, the rate of protein synthesis $k_{p}$ (a measurement scaling factor) and the maturation rate $k_{m}$ were left to be estimated from the data.

After some simple preprocessing steps of the model and the data (Supplementary Sections S3.3 and S3.4), we estimated the joint distribution of $k_{p}$ and $k_{m}$ across a cell population for each of the 12 FPs quantified in Guerra *et al.* (2022) using GMGTS. In contrast to the *in silico* studies above, where normal population distributions were inferred in the second GMGTS stage, here we assumed that $k_{p}$ and $k_{m}$ follow a log-normal distribution. The assumption of variable $k_{m}$ is supported by our data, since the model with a common maturation rate had consistently lower marginal likelihood compared to the models with variable maturation rate for all FPs in our dataset (Supplementary Section S13.2 and Supplementary Table S3).

To infer the parameters of the log-normal population distributions, we used an approximate inference scheme described in Supplementary Section S5. Figure 5C summarizes the estimated maturation half-time distributions, ordered with respect to their corresponding mean rates. A description of how these distributions were obtained from the log-normally distributed maturation rates, as well as an analytical approximation of the maturation half-time for two-step maturation models, are provided in Supplementary Sections S3.5 and S3.6. Supplementary Figs S10–S21 show the full random effects distributions and the measurements overlaid with the predicted state distributions, as inferred by GMGTS for each FP.

The estimates of Fig. 5 confirm the empirical observation that proteins with a single rate-limiting maturation step are faster than proteins with two steps, while the means of the population distributions are largely in line with those obtained in Guerra *et al.* (2022). Similarly to Wu *et al.* (2020), FPs seem to display considerable intercellular variability in terms of maturation half-times, with fast-maturing proteins varying more compared to slow-maturing proteins (Supplementary Table S4).

## 4 Discussion

Dynamic NLME models offer a computationally efficient alternative to the fully stochastic models of intracellular dynamics when the main sources of cell-to-cell variability are extrinsic to the system of interest and vary slowly compared to the system dynamics. Still, parameter inference for this class of models remains a computationally intensive task. To speed up the inference of dynamic NLME models, we presented the Gradient Matching Global Two-Stage (GMGTS) method, an adaptation of the well-established Global Two-Stage (GTS) approach (Davidian and Giltinan 2017). GMGTS relies on gradient matching to obtain first-stage individual parameter estimates which are subsequently used to estimate the population parameters via Expectation-Maximization. When the underlying dynamical system is linear in parameters, the first stage of GMGTS solves a linear regression problem that replaces the nonconvex optimization required by the trajectory matching approach. Thanks to the speed of GMGTS, tuning of the method is quite straightforward since the user gets near-instant feedback.

We demonstrated the computational efficiency of GMGTS compared to GTS in various *in silico* tests and showed that GMGTS achieves satisfactory accuracy across a wide range of data sparsity and noise levels. If greater accuracy in the first-stage individual estimates is needed (e.g. in the presence of highly sparse or noisy data), the GMGTS estimates can be further refined using a trajectory matching step. Initializing the optimization with the individual GMGTS estimates should significantly accelerate convergence compared to a random starting point, with minimal additional cost.

Having established the performance of our method on simulated data, we applied it to recover the maturation times of fluorescent proteins using single-cell fluorescence time series in budding yeast. Our findings are in line with current literature in terms of the mean maturation times (Guerra *et al.* 2022), but we additionally observed that maturation rates show considerable variability across isogenic populations. The origins of this variability are unknown, but it has been conjectured that it is caused by cell-to-cell differences in the intracellular redox state (Wu *et al.* 2020). Due to their implications for single-cell fluorescence microscopy experiments, these results warrant further investigation via an orthogonal experimental method.

Besides introducing gradient matching for mixed-effects model inference, GMGTS addresses two shortcomings of commonly used gradient matching methods: reliance on full state information and lack of provision of parameter uncertainties. To address the former, we developed an algorithm that treats unobserved states as latent variables and estimates them iteratively together with the system parameters. This scheme relies on a small number of numerical integration runs which is insignificant compared to the hundreds of runs that trajectory matching generates for each initial point during optimization. We have also addressed the lack of parameter uncertainties for both fully and partially observed systems by propagating the measurement variability to the individual parameter estimates via the estimated states and their gradients.

Our gradient matching approach is based on and extends that of Varah (1982). Alternative approaches have been proposed, such as gradient matching using Gaussian processes (GPs) (Calderhead *et al.* 2009, Dondelinger *et al.* 2013, Barber and Wang 2014) and the generalized profiling method (Ramsay *et al.* 2007). While these methods are also able to handle unobserved states, to the best of our knowledge, no parameter uncertainty computations have been provided for them. Moreover, GP-based approaches require the estimation of posterior distributions whose evaluation is analytically intractable and instead necessitates computationally intensive sampling schemes. While generalized profiling does not have this limitation, it solves a nonconvex optimization problem over a large number of parameters by optimizing the parameters of the smoothing estimates and ODEs simultaneously. Smoothing the experimental data using cubic B-splines prior to gradient matching (as we do here) implies that smoothing estimates are not constrained by the ODE structure. However, our approach provides a significant computational edge to GMGTS at the cost of a manageable loss in estimation accuracy while properly accounting for parameter uncertainties.

Since smoothing precedes parameter estimation in GMGTS, careful smoothing of the measurements is important for obtaining accurate estimates of population parameters. To facilitate and speed up this process, we have implemented an interactive smoothing app to provide immediate visual feedback on the choice of the B-spline knot locations. The app also provides smoothing estimates using an automated tuning heuristic which performed closely to manual knot selection in our tests (Supplementary Section S11.1). The smooth estimates obtained via the app are directly fed into our GMGTS implementation, making it easier to assess individual parameter estimates obtained with different smoothing settings. While we chose B-splines for their simplicity and flexibility, we should note that GMGTS can be seamlessly adapted to other types of (penalized) splines or Gaussian process-based smoothing, since it only requires estimates of smoothed trajectories, their gradients, and their covariances.

Besides uncertainty in parameterization, biochemical systems may also display variability in their initial conditions. Our GMGTS implementation assumes that initial conditions are known, and therefore cannot handle systems with unknown initial conditions. This is not a major limitation in the fully observed case, where the smoothing of experimental data also provides initial condition estimates. As discussed in Varah (1982), these estimates can be further refined via a trajectory matching step after gradient matching is completed. Inference of unknown initial conditions is less straightforward in the partially observed case, where a filtering approach (Särkkä and Svensson 2023) could be used to infer hidden states and their initial conditions. The details of such a scheme remain the topic of future work.

With respect to uncertainty modeling, GTS is typically based on the assumption that individual parameters follow a multivariate normal distribution. Though biochemical network parameters are often nonnegative, this assumption suffices if their coefficient of variation is not too large. On the other hand, normality is only imposed in the second (GM)GTS stage (i.e. the EM algorithm) while the first stage is distribution-agnostic. Postulating a nonnormal distribution for the individual parameters is also possible, though it requires more complex deconvolution methods (Efron 2016) to infer the population parameters. Since log-normal parameter distributions are often encountered in biochemical network models (Eydgahi *et al.* 2013, Geris and Gomez-Cabrero 2016, Tsigkinopoulou *et al.* 2017), we presented two alternative approximate inference schemes tailored for inferring log-normal population distributions in Supplementary Section S5.

In summary, GMGTS is a highly efficient and powerful inference method for dynamic NLME models that is also highly extensible, serving as the basis for the development of more powerful and general inference methods in the future.

## Supplementary Material

btaf154_Supplementary_Data

## Data Availability

No new data were generated or analysed in support of this research.
