# Supplementary material for: Gradient matching accelerates mixed-effects inference for biochemical networks
Source: Bioinformatics. 2025 Apr 8;41(4):btaf154. doi: 10.1093/bioinformatics/btaf154 (PMC12034378; doi:10.1093/bioinformatics/btaf154)
Supplement: btaf154_Supplementary_Data [file btaf154_supplementary_data.pdf]

Supplemental Information

Gradient matching accelerates mixed-effects inference for biochemical networks

Yulan B. van Oppen and Andreas Milias-Argeitis*

Groningen Biomolecular Sciences and Biotechnology Institute
University of Groningen, The Netherlands

Contents

S1 Notation **1**

S2 Methodological details **1**
S2.1 Model . . . 1
S2.2 Global Two-Stage (GTS) aproach for nonlinear mixed-effects models . . . 2
S2.3 The Gradient Matching Global Two-Stage (GMGTS) approach . . . 3
S2.4 GMGTS for partially observed systems . . . 5

S3 Pre- and post-processing of dynamical systems **5**
S3.1 Reformulation of the repressilator equations . . . 5
S3.2 Bifunctional two-component system dynamics in terms of the total abundances of phosphorylated and free H and R 6
S3.3 Reformulation of the delay-differential equations for one- and two-step fluorescent protein maturation with an ODE system . . . 7
S3.4 Preprocessing of experimental data obtained through fluorescence microscopy . . . 8
S3.5 Approximation of an equivalent maturation half-time for proteins undergoing two-step maturation . . . 9
S3.6 The distribution of maturation half-times . . . 9

S4 Derivation of the second-stage EM algorithm **9**

S5 Inference of log-normal random effects distributions **10**
S5.1 Alternative 1: approximation of first-stage normal distributions by log-normal ones . . . 10
S5.2 Alternative 2: approximation of the second-stage marginal likelihood . . . 11
S5.3 Performance of the approximations . . . 13

S6 B-spline smoothing **13**

S7 Full observation: estimation of the residual covariance matrix and the covariances of individual parameter estimates **14**

S8 Partial observation: estimation of the residual covariance matrix and the covariance of individual parameter estimates **16**

S9 Including prior information and modifications for maximum a posteriori estimates **18**

S10 Convergence of GMGTS with partial state information **19**

S11 Implementation details of GTS and GMGTS **21**
S11.1 Smoothing . . . 21
S11.2 Individual gradient matching estimates with full observation . . . 23
S11.3 Individual gradient matching estimates with partial observation . . . 23
S11.4 GTS settings used for performance comparisons . . . 24
S11.5 Wasserstein distance between multivariate normal distributions . . . 24

S12 Figures related to simulation studies **26**

S13 Results related to the inference of FP maturation rates **28**
S13.1 Neglecting dilution rate variability does not significantly inflate the variability of maturation rate estimates . . . 28

*Correspondence: a.milias.argeitis@rug.nl

S13.2 Comparison of FP maturation models with common vs. variable maturation rate 29

S14 Additional results on the inference of variable FP maturation rates **29**

References **42**

List of Tables

S1 Selected B-spline knots for each system 23
S2 Simulation study on ignoring dilution rate variability in the FP maturation system 29
S3 Marginal likelihood values of FP maturation models with fixed vs. variable maturation rate 29
S4 Summary of inferred maturation half time distributions and comparison with previously published results 29

List of Figures

S1 Repressilator trajectories obtained from the expanded and original system equations 7
S2 Level sets of log-normal ground-truth distributions for the maturation system and their approximations 13
S3 Dependence hierarchy in the iterative optimization scheme for partially observed systems 17
S4 Basins of attraction for individual parameter estimates obtained by GMGTS for partially observed systems. 20
S5 Comparison of smoothing using manually selected knots and the knot placement heuristic 22
S6 Normalized Wasserstein distances for various differences in distributions 25
S7 Simulation study of the partially observed bifunctional two-component system: examples of inference and prediction results 26
S8 Simulation study of partially observed FP maturation model: illustration of inference and prediction results 27
S9 Simulation study of the partially observed repressilator: illustration of inference and predictions results 28
S10 sfGFP: parameter inference and state predictions. 30
S11 CFP: parameter inference and state predictions. 31
S12 pHtdGFP: parameter inference and state predictions. 32
S13 mNeonGreen: parameter inference and state predictions. 33
S14 mCitrine: parameter inference and state predictions. 34
S15 mVenus: parameter inference and state predictions. 35
S16 mScarlet-I: parameter inference and state predictions. 36
S17 mCherry: parameter inference and state predictions. 37
S18 mTurquoise2: parameter inference and state predictions. 38
S19 mTFP1: parameter inference and state predictions. 39
S20 tdTomato: parameter inference and state predictions. 40
S21 mKate2: parameter inference and state predictions. 41

S1 Notation

This section briefly lists some national conventions used in the supplemental mathematical derivations. Numerous references are also made to the main paper; to be able to clearly discern referenced sections and equations, all items appearing in this document are numbered starting with 'S'. Moreover:

- Vectors and matrices are written in boldface. They can be defined in terms of scalars or smaller vectors or matrices by enclosing these quantities in square brackets. For matrices $\mathbf{A}_1, \ldots, \mathbf{A}_n \in \mathbb{R}^{N_1 \times N_2}$ (where possibly $N_1 = N_2 = 1$), we define a "stacked" matrix using the shorthand notation of a comma-separated bracketed list; that is,

$$[\mathbf{A}_1, \ldots, \mathbf{A}_n] := \begin{bmatrix} \mathbf{A}_1 \\ \vdots \\ \mathbf{A}_n \end{bmatrix} \in \mathbb{R}^{nN_1 \times N_2}.$$

  In particular, we let $[a_1, \ldots, a_n]$ denote an n-dimensional column vector with elements $a_1, \ldots, a_n$. Note that if the elements of a block matrix (or vector) are *not* separated by commas, then no stacking operation is implied.

- Elements of vectors $\mathbf{v}$ and matrices $\mathbf{A}$ are written in non-boldface, with the element index in subscript. So, the j-th element of $\mathbf{v}$ and the (i, j)-th element of $\mathbf{A}$ are written as

$$v_j \quad \text{and} \quad A_{jk}.$$

  In case vectors $\mathbf{v}_i$ and matrices $\mathbf{A}_i$ are already indexed (in this case, by i), we their j-th and (j, k)-th element as

$$v_{i,j} \quad \text{and} \quad A_{i,jk},$$

  respectively. If no ambiguity arises, they are also written as v_{ij} and A_{ijk}.

- Vectors are assumed to be columns unless noted otherwise.
- For vectors $\mathbf{v}$ and matrices $\mathbf{A}$, $\mathbf{v}'$ and $\mathbf{A}'$ denote the respective transposes.
- The determinant of a matrix $\mathbf{A}$ is denoted by $|\mathbf{A}|$.
- The Kronecker product between matrices $\mathbf{A}$ and $\mathbf{B}$ is written as $\mathbf{A} \otimes \mathbf{B}$.
- Whenever a quantity $\mathbf{A}$ is a function of estimates $\hat{\mathbf{B}}, \hat{\mathbf{C}}$ of (scalar, vector, or matrix) quantities $\mathbf{B}, \mathbf{C}$, we write

$$\mathbf{A}_{\mathbf{B}}(\hat{\mathbf{B}}, \hat{\mathbf{C}}) := \frac{\partial}{\partial \mathbf{B}} \mathbf{A}(\hat{\mathbf{B}}, \hat{\mathbf{C}}) := \frac{\partial}{\partial \mathbf{B}} \mathbf{A}(\mathbf{B}, \mathbf{C}) \bigg|_{(\mathbf{B},\mathbf{C})=(\hat{\mathbf{B}},\hat{\mathbf{C}})}.$$

  Additionally, wherever it is clear from the context, $\mathbf{A}(\hat{\mathbf{B}}, \hat{\mathbf{C}})$ may be abbreviated as $\hat{\mathbf{A}}$. Using this notation, we write

$$\hat{\mathbf{A}}_{\mathbf{B}} := \frac{\partial}{\partial \mathbf{B}} \hat{\mathbf{A}} := \frac{\partial}{\partial \mathbf{B}} \mathbf{A}(\hat{\mathbf{B}}, \hat{\mathbf{C}}).$$

- The $(n \times n)$-dimensional identity matrix is denoted by $\mathbf{I}_n$ and the respective $(n \times m)$-dimensional matrices of zeros is denoted by $\mathbf{0}_{n \times m}$; we also write $\mathbf{0}_n := \mathbf{0}_{n \times n}$.

S2 Methodological details

S2.1 Model

Consider a population of N cells, each having an underlying mechanistic model given by a K-dimensional ODE system:

$$\dot{\mathbf{x}}_i(t) = \mathbf{f}(\mathbf{x}_i(t); \boldsymbol{\beta}_i) \quad \text{for} \quad t \geqslant 0 \quad \text{and} \quad i = 1, \ldots, N, \tag{S2.1}$$

where $\mathbf{f} : \mathbb{R}^K \times \mathbb{R}^P \to \mathbb{R}^K$ is common for all cells. The dynamics of the i-th cell are specified by a set of known initial conditions $\mathbf{x}_i(0) = \mathbf{x}_i^0$ and an unknown P-dimensional parameter vector $\boldsymbol{\beta}_i$. We assume that $\boldsymbol{\beta}_i$ is drawn from a normal distribution with mean vector $\mathbf{b}$ and covariance matrix $\mathbf{D}$, i.e.

$$\boldsymbol{\beta}_1, \ldots, \boldsymbol{\beta}_N \overset{\text{iid}}{\sim} \mathrm{N}(\mathbf{b}, \mathbf{D}). \tag{S2.2}$$

An alternative modeling assumption is that $\boldsymbol{\beta}_i$ follows a log-normal distribution, that is

$$\boldsymbol{\beta}_1, \ldots, \boldsymbol{\beta}_N \overset{\text{iid}}{\sim} \mathrm{LN}(\mathbf{b}, \mathbf{D}), \tag{S2.3}$$

where $\mathbf{b}$ is a log-mean vector and $\mathbf{D}$ a log-covariance matrix. Equations (S2.1) (the individual model) and (S2.2) or (S2.3) (the population model) comprise an *ODE-based mixed-effects* model, where $\boldsymbol{\beta}_1, \ldots, \boldsymbol{\beta}_N$ are called *random effects* vectors, $\mathbf{b}$ is the *fixed effects* vector, and $\mathbf{D}$ is the *random effects covariance matrix.*

The measurements for each cell i are assumed to be a T-dimensional vector of noisy samples of $\mathbf{x}^*(t;\boldsymbol{\beta}_i)$, the solution to (S2.1) corresponding to parameter vector $\boldsymbol{\beta}_i$; that is,

$$\mathbf{y}_i(t) = \mathbf{Q}\mathbf{x}^*(t;\boldsymbol{\beta}_i) + \varepsilon_i(t) \quad \text{for} \quad t = t_1, \ldots, t_T \tag{S2.4}$$

In (S2.4), $\mathbf{Q}$ is a binary matrix used to select the observed components of $\mathbf{x}_i^*(t;\boldsymbol{\beta}_i)$ and $\varepsilon_i(t)$ is a vector containing the measurement noise for each state. We consider the measurement dimension to be common across cells, but accommodating different measurement times for each cell is straightforward. Not all states are necessarily observed in practice. Whenever that is the case, we assume without loss of generality only the first $L \leqslant K$ states are measured, i.e., $\mathbf{Q}$ consists of the first L rows of the K-dimensional identity matrix. The methodology described in the following sections readily extends to (possibly nonlinear) measurement functions $\mathbf{Q}: \mathbb{R}^K \to \mathbb{R}^L$ of the underlying process $\mathbf{x}_i(t)$ with a differentiable partial inverse, such as a partially invertible linear map.

The measurement errors $\varepsilon_i(t_j)$ are assumed to be a combination of additive and multiplicative noise, and hence have components $\varepsilon_{ik}(t_j)$ independently distributed as

$$\varepsilon_{ik}(t_j) \sim \mathrm{N}\left(0, \sigma_k^2 + \tau_k^2\left[x_{ik}^*(t_j;\boldsymbol{\beta}_i)\right]^2\right) \quad \text{for} \quad k = 1, \ldots, L \tag{S2.5}$$

for some $\sigma_k, \tau_k \geqslant 0$ taken to be common across cells, and $x_{ik}^*(t_j;\boldsymbol{\beta}_i)$ denotes the k-th component of $\mathbf{x}_i^*(t_j;\boldsymbol{\beta}_i)$.

In the context of gradient matching (discussed in more detail in a later section), we focus on the special case where $\mathbf{f}$ is linear in the unknown parameters $\boldsymbol{\beta}_i$. In that case, (S2.1) for the i-th cell can be decomposed as

$$\dot{\mathbf{x}}_i(t) = \mathbf{g}(\mathbf{x}_i(t))\boldsymbol{\beta}_i + \mathbf{h}(\mathbf{x}_i(t)) \quad \text{for} \quad t \geqslant 0. \tag{S2.6}$$

Here, $\mathbf{g}: \mathbb{R}^K \to \mathbb{R}^K \times \mathbb{R}^P$ and $\mathbf{h}: \mathbb{R}^K \to \mathbb{R}^K$ are common across cells and independent of $\boldsymbol{\beta}_i$, but neither is restricted to be linear in $\mathbf{x}_i(t)$.

S2.2 Global Two-Stage (GTS) aproach for nonlinear mixed-effects models

Here, we provide a brief overview of the *global two-stage* (GTS) approach [Davidian and Giltinan, 2017, Ch. 5] which can be used for inference of ODE-based mixed-effects models. In GTS, estimates of individual cell parameters $\boldsymbol{\beta}_i$ are obtained in the first stage, along with estimates of their associated precisions $\mathbf{C}_i^{-1}$. In the second stage, the random effects distribution is inferred from the estimated $\boldsymbol{\beta}_i$'s, taking into account their covariances $\mathbf{C}_i$. Specifically, for the model described by (S2.1), (S2.2), (S2.4) and (S2.5), GTS proceeds as follows:

I. For each $i = 1, \ldots, N$, estimate $\boldsymbol{\beta}_i$ by minimizing the *weighted sum of squares* (WSS)

$$\mathrm{WSS}_i(\boldsymbol{\beta}_i) := \sum_{k=1}^{K}\sum_{j=1}^{T} \frac{\left(y_{ik}(t_j) - x_{ik}^*(t_j;\boldsymbol{\beta}_i)\right)^2}{\sigma_k^2 + \tau_k^2\left[x_{ik}^*(t_j;\boldsymbol{\beta}_i)\right]^2}, \tag{S2.7}$$

where $y_{ik}(t_j)$ denotes the k-th component of $\mathbf{y}_{ik}(t_j)$. In practice, the measurement model parameters σ_k, τ_k are unknown and need to be iteratively estimated together with the system parameters. This joint estimation problem is solved with a *feasible weighted least squares* (FWLS) algorithm [Greene, 2003, Ch. 10]. Given an estimate $\hat{\boldsymbol{\beta}}_i$ of $\boldsymbol{\beta}_i$, consistent estimates of the measurement model parameters can be obtained by maximizing the following log-likelihood function for each state:

$$\hat{l}_k(\sigma_k, \tau_k) := \sum_{i=1}^{N}\sum_{j=1}^{T}\left[-\frac{1}{2}\frac{\left(y_{ik}(t_j) - x_{ik}^*(t_j;\hat{\boldsymbol{\beta}}_i)\right)^2}{\sigma_k^2 + \tau_k^2\left[x_{ik}^*(t_j;\hat{\boldsymbol{\beta}}_i)\right]^2} - \frac{1}{2}\log\left(\sigma_k^2 + \tau_k^2\left[x_{ik}^*(t_j;\hat{\boldsymbol{\beta}}_i)\right]^2\right)\right]. \tag{S2.8}$$

Let $\widehat{\mathrm{WSS}}_i(\boldsymbol{\beta}_i)$ denote $\mathrm{WSS}_i(\boldsymbol{\beta}_i)$ when σ_k and τ_k are substituted by their estimators. Each estimate of $\boldsymbol{\beta}_i$ may then be obtained by means of FWLS using the following scheme:

1. Initialize variance parameters, e.g., each $\hat{\sigma}_k = 1$ and $\hat{\tau}_k = 0$.
2. Update $\hat{\boldsymbol{\beta}}_i \leftarrow \arg\min_{\boldsymbol{\beta}_i} \widehat{\mathrm{WSS}}_i(\boldsymbol{\beta}_i)$ using the current variance parameter estimates $\hat{\sigma}_k, \hat{\tau}_k$; cf. (S2.7).
3. Update $(\hat{\sigma}_k, \hat{\tau}_k) \leftarrow \arg\max_{\sigma_k,\tau_k} \hat{l}_k(\sigma_k, \tau_k)$ using the current parameter estimate $\hat{\boldsymbol{\beta}}_i$; cf. (S2.8);
4. Repeat steps 2 and 3 until convergence.

Estimates $\hat{\boldsymbol{\beta}}_i$ are produced for each cell $i = 1, \ldots, N$. Subsequently, each error covariance matrix $\mathbf{C}_i$ is estimated using the Hessian of $\widehat{\mathrm{WSS}}_i(\hat{\boldsymbol{\beta}}_i)$, i.e.,

$$\widehat{\mathbf{C}}_i := -2\left(\frac{\partial^2\widehat{\mathrm{WSS}}_i}{\partial\boldsymbol{\beta}_i\partial\boldsymbol{\beta}_i'}(\hat{\boldsymbol{\beta}}_i)\right)^{-1}.$$

II. Infer the population parameters $\mathbf{b}$ and $\mathbf{D}$ via maximum likelihood using an *expectation maximization* (EM) algorithm. In this algorithm, the random effects $\boldsymbol{\beta}_i$ are considered to be latent variables, and their estimates $\hat{\boldsymbol{\beta}}_i$ from the first stage are treated as

data. As stated in [Davidian and Giltinan, 2017, § 5.3.2], using an asymptotic approximation as the number of measured time points (T) increases, we have

$$\hat{\boldsymbol{\beta}}_i \mid \boldsymbol{\beta}_i \sim \mathrm{N}(\boldsymbol{\beta}_i, \mathbf{C}_i), \quad \text{and marginally,} \quad \hat{\boldsymbol{\beta}}_i \sim \mathrm{N}(\mathbf{b}, \mathbf{D} + \mathbf{C}_i). \tag{S2.9}$$

We initialize the estimates $\widehat{\mathbf{b}}$ and $\widehat{\mathbf{D}}$ using the sample mean and covariance of $\hat{\boldsymbol{\beta}}_1, \ldots, \hat{\boldsymbol{\beta}}_N$. Treating $\boldsymbol{\beta}_i$ as latent variables, their estimates $\tilde{\boldsymbol{\beta}}_i$ are initialized at their respective first stage estimate $\hat{\boldsymbol{\beta}}_i$. At the *expectation step* (E-step), we compute the expectation of $\boldsymbol{\beta}_i$ conditional on $\hat{\boldsymbol{\beta}}_i$ and the current estimates $\widehat{\mathbf{b}}$ and $\widehat{\mathbf{D}}$ for each cell. This means that we update

$$\tilde{\boldsymbol{\beta}}_i \leftarrow \left(\widehat{\mathbf{D}}^{-1} + \mathbf{C}_i^{-1}\right)^{-1}\left(\widehat{\mathbf{D}}^{-1}\widehat{\mathbf{b}} + \mathbf{C}_i^{-1}\hat{\boldsymbol{\beta}}_i\right). \tag{E-step}$$

Next, we update $\widehat{\mathbf{b}}$ and $\widehat{\mathbf{D}}$ given their current values and each updated $\tilde{\boldsymbol{\beta}}_i$ in the *maximization step* (M-step) as

$$\widehat{\mathbf{b}} \leftarrow \frac{1}{N}\sum_{i=1}^{N}\tilde{\boldsymbol{\beta}}_i \quad \text{and} \quad \widehat{\mathbf{D}} \leftarrow \frac{1}{N}\sum_{i=1}^{N}\left[\left(\widehat{\mathbf{D}}^{-1} + \widehat{\mathbf{C}}_i^{-1}\right)^{-1} + \left(\tilde{\boldsymbol{\beta}}_i - \widehat{\mathbf{b}}\right)\left(\tilde{\boldsymbol{\beta}}_i - \widehat{\mathbf{b}}\right)'\right]. \tag{M-step}$$

For a derivation of these update steps, see § S4. The EM algorithm iterates (E-step) and (M-step) until convergence. The refined estimates $\tilde{\boldsymbol{\beta}}_i$ are called *empirical Bayes predictors*, and correspond to modified first-stage estimates that are shrunk toward the population mean. In this work, we disregard these estimates and focus on the estimated population parameters $\widehat{\mathbf{b}}$ and $\widehat{\mathbf{D}}$ and random effects $\hat{\boldsymbol{\beta}}_i$.

Remark S1. Note that each evaluation of $\widehat{\mathrm{WSS}}_i(\boldsymbol{\beta}_i)$ generally requires a numerical solution of (S2.1) to be computed. Moreover, the optimization problem of the cell-specific model parameters is typically non-convex [Schittkowski, 2002].

Remark S2. The first stage of the method does not depend on the assumption (S2.2) that random effects follow a multivariate normal distribution in the population. However, the normality assumption becomes important in the second stage, where the population parameters are inferred. Using another distributional assumption for the random effects would necessitate the solution of a deconvolution problem, which can be technically challenging. For this reason, the normality assumption (S2.2) is standard in the mixed-effects literature. In § S5, we present two approximate solutions of the deconvolution problem when (S2.3) is used in place of (S2.2).

S2.3 The Gradient Matching Global Two-Stage (GMGTS) approach

Gradient matching [Varah, 1982] can be applied to any nonlinear dynamical system, but it is particularly efficient for systems that are linear in parameters (S2.6). GMGTS replaces trajectory matching with gradient matching in the first step of the GTS method. Using the uncertainty estimates for the cell-specific estimates produced in the first stage, the second stage remains unmodified (see § S2.2). This section provides details on the first stage of GMGTS corresponding to the case of full state information, i.e. when $L = K$. For the implementation details of this stage, see § S11. A more involved procedure (presented in the next section) is required for partially observed systems since modifications are needed to estimate the hidden states.

In brief, the following steps comprise the first stage of the GMGTS method. Treating each cell $i = 1, \ldots, N$ independently, we:

I.A Smooth the measurements $y_{ik}(t)$ with a B-spline $\hat{x}_{ik}(t)$ using FWLS for each state $k = 1, \ldots, K$, with weights dependent on the estimated measurement error variance. The details of the smoothing method are given in § S6.

I.B Use the splines estimated in Step I.A to approximate (S2.6), where the left-hand side is obtained by differentiating the splines, while the right-hand side is produced from substitution of the splines into $\mathbf{g}(\cdot)$ and $\mathbf{h}(\cdot)$. Denoting the spline approximations by $\hat{\mathbf{x}}_i(t) := (\hat{x}_{i1}(t), \ldots, \hat{x}_{iL}(t))$ for $t_1 \leqslant t \leqslant t_T$, we introduce these splines in (S2.6) with the goal of finding $\boldsymbol{\beta}_i$ so that

$$\dot{\hat{\mathbf{x}}}_i(t) \approx \mathbf{g}(\hat{\mathbf{x}}_i(t))\boldsymbol{\beta}_i + \mathbf{h}(\hat{\mathbf{x}}_i(t)) \quad \text{for} \quad t_1 \leqslant t \leqslant t_T. \tag{S2.10}$$

Since $\boldsymbol{\beta}_i$ is assumed to be independent of t, we can stack the matrices containing the spline data in (S2.10) to obtain

$$\begin{bmatrix} \dot{\hat{\mathbf{x}}}_i(t_1) \\ \vdots \\ \dot{\hat{\mathbf{x}}}_i(t_T) \end{bmatrix} - \begin{bmatrix} \mathbf{h}(\hat{\mathbf{x}}_i(t_1)) \\ \vdots \\ \mathbf{h}(\hat{\mathbf{x}}_i(t_T)) \end{bmatrix} \approx \begin{bmatrix} \mathbf{g}(\hat{\mathbf{x}}_i(t_1)) \\ \vdots \\ \mathbf{g}(\hat{\mathbf{x}}_i(t_T)) \end{bmatrix} \boldsymbol{\beta}_i. \tag{S2.11}$$

The arrangement (S2.11) facilitates the estimation of $\boldsymbol{\beta}_i$ by means of convex optimization, such as generalized linear regression. The challenge lies in deriving a covariance matrix estimate for the residuals of (S2.11).

The remainder of this section is dedicated to the details of the gradient matching procedure performed in Step I.B. Since the model parameters for different cells are optimized independently at this stage, we drop the subscripts i to simplify the notation, writing $\boldsymbol{\beta} := \boldsymbol{\beta}_i$ and $\mathbf{x} := \mathbf{x}_i$ and following the same convention for estimates or components of these vectors. Denote the stacked matrices of spline approximations $\hat{\mathbf{x}}(t)$, their derivatives $\dot{\hat{\mathbf{x}}}(t)$, and function evaluations $\mathbf{g}(\hat{\mathbf{x}}(t)), \mathbf{h}(\hat{\mathbf{x}}(t))$ as

$$\hat{\mathbf{X}} := \begin{bmatrix} \hat{\mathbf{x}}(t_1) \\ \vdots \\ \hat{\mathbf{x}}(t_T) \end{bmatrix}, \quad \dot{\hat{\mathbf{X}}} := \begin{bmatrix} \dot{\hat{\mathbf{x}}}(t_1) \\ \vdots \\ \dot{\hat{\mathbf{x}}}(t_T) \end{bmatrix}, \quad \hat{\mathbf{G}} := \mathbf{G}(\hat{\mathbf{X}}) := \begin{bmatrix} \mathbf{g}(\hat{\mathbf{x}}(t_1)) \\ \vdots \\ \mathbf{g}(\hat{\mathbf{x}}(t_T)) \end{bmatrix}, \quad \text{and} \quad \hat{\mathbf{H}} := \mathbf{H}(\hat{\mathbf{X}}) := \begin{bmatrix} \mathbf{h}(\hat{\mathbf{x}}(t_1)) \\ \vdots \\ \mathbf{h}(\hat{\mathbf{x}}(t_T)) \end{bmatrix},$$

allowing us to transform (S2.11) into a formal model

$$\hat{\hat{\mathbf{X}}} - \hat{\mathbf{H}} = \hat{\mathbf{G}}\beta + \boldsymbol{\Delta}, \qquad \boldsymbol{\Delta} \sim \mathrm{N}(\mathbf{0}, \mathbf{V}). \tag{S2.12}$$

Although (S2.12) suggests β can be any real vector, in practical situations it makes sense to limit the parameter search space to avoid nonsensical parameter estimates (e.g. negative values of rate constants due to overfitting) and prevent numerical instabilities. We therefore assume

$$\beta \in (c_1, d_1) \times \cdots \times (c_P, d_P) \subseteq \mathbb{R}^P, \qquad c_p, d_p \in \mathbb{R} \cup \{\pm\infty\} \quad \text{for} \quad p = 1, \ldots, P. \tag{S2.13}$$

More complex parameter spaces may also be considered, as long as they are defined by linear inequalities. Even though the presence of constraints may be at odds with a population distribution that has unbounded support, the impact of the bounds should be minimal, as long as most of the probability lies within these bounds. An alternative to imposing parameter bounds is the use of a prior distribution, which is further discussed in § S9.

Equation (S2.12) is a generalized linear regression model with response vector $\hat{\hat{\mathbf{X}}} - \hat{\mathbf{H}}$, design matrix $\hat{\mathbf{G}}$, and a (multivariate) normally distributed vector of residuals $\boldsymbol{\Delta}$. The main difficulty of this model is the fact that the covariance structure of the residuals is not straightforward to obtain, since the uncertainty in the measurements propagates to $\hat{\mathbf{G}}$ through the smoothed measurements $\hat{\mathbf{x}}_i(t)$. We address this problem by calculating a delta-method approximation of the covariance matrix of $\boldsymbol{\Delta}$ conditional on β, that is

$$\mathrm{Var}\{\boldsymbol{\Delta} \mid \beta\} = \mathrm{Var}\{\hat{\hat{\mathbf{X}}} - \hat{\mathbf{G}}\beta - \hat{\mathbf{H}} \mid \beta\} \approx \left[-\frac{\partial}{\partial \hat{\mathbf{X}}}\left(\mathbf{G}(\hat{\mathbf{X}})\beta + \mathbf{H}(\hat{\mathbf{X}})\right) \quad \mathbf{I}_{TK}\right] \mathrm{Var}\begin{bmatrix}\hat{\mathbf{X}} \\ \hat{\hat{\mathbf{X}}}\end{bmatrix} \left[-\frac{\partial}{\partial \hat{\mathbf{X}}}\left(\mathbf{G}(\hat{\mathbf{X}})\beta + \mathbf{H}(\hat{\mathbf{X}})\right) \quad \mathbf{I}_{TK}\right]' =: \hat{\mathbf{V}}(\beta),$$

where $\mathrm{Var}[\hat{\mathbf{X}}, \hat{\hat{\mathbf{X}}}]$ is given in (S7.4) and follows from the B-spline smoothing procedure (see § S6 for smoothing and § S7 for the calculation of $\hat{\mathbf{V}}(\beta)$ in terms of the smoothing estimates).

The respective estimates $\hat{\beta}$ and $\hat{\mathbf{V}} := \hat{\mathbf{V}}(\hat{\beta})$ of the model parameters and the residual covariance matrix need to be computed iteratively in a *feasible generalized least squares* (FGLS) algorithm [Greene, 2003]:

1. Initialize $\hat{\mathbf{V}}$ at the TK-dimensional identity matrix (cf. Remark S3).

2. Given the current value of $\hat{\mathbf{V}}$, update the model parameter estimate $\hat{\beta}$ by minimizing the generalized sum of squares criterion

$$\mathrm{GSS}(\beta) = \left(\hat{\hat{\mathbf{X}}} - \hat{\mathbf{G}}\beta - \hat{\mathbf{H}}\right)' \hat{\mathbf{V}}^{-1} \left(\hat{\hat{\mathbf{X}}} - \hat{\mathbf{G}}\beta - \hat{\mathbf{H}}\right), \tag{S2.14}$$

   When parameter constraints are present, minimization of (S2.14) leads to a constrained least squares problem. This gives rise to a convex quadratic program which can be solved efficiently via a quadratic programming active-set method. When the optimum is not located on the boundary of the parameter space (see Remark S5), it coincides with the generalized least squares estimate of β:

$$\hat{\beta} := \left(\hat{\mathbf{G}}'\hat{\mathbf{V}}^{-1}\hat{\mathbf{G}}\right)^{-1} \hat{\mathbf{G}}'\hat{\mathbf{V}}^{-1} \left(\hat{\hat{\mathbf{X}}} - \hat{\mathbf{H}}\right). \tag{S2.15}$$

3. Update $\hat{\mathbf{V}} := \hat{\mathbf{V}}(\hat{\beta})$ according to (S7.6) using the updated estimate $\hat{\beta}$.

4. Repeat Step 2 and Step 3 until convergence (see § S11 for the implementation details of this scheme).

Since the $\hat{\mathbf{X}}$ appears on the right-hand side of the generalized linear model, using the Fisher information matrix corresponding to (S2.14) to approximate the uncertainty of the final estimate $\hat{\beta}$ leads to an upward bias in terms of precision. Rather, we estimate this uncertainty using a delta-method approximation [Berger and Casella, 2001, § 5.5] as

$$\mathbf{C} := \mathrm{Var}\,\hat{\beta} \approx \begin{bmatrix}\frac{\partial}{\partial \hat{\mathbf{X}}}\hat{\beta} \\ \frac{\partial}{\partial \hat{\hat{\mathbf{X}}}}\hat{\beta}\end{bmatrix} \mathrm{Var}\begin{bmatrix}\hat{\mathbf{X}} \\ \hat{\hat{\mathbf{X}}}\end{bmatrix} \begin{bmatrix}\frac{\partial}{\partial \hat{\mathbf{X}}}\hat{\beta} \\ \frac{\partial}{\partial \hat{\hat{\mathbf{X}}}}\hat{\beta}\end{bmatrix}', \tag{S2.16}$$

where $\mathrm{Var}[\hat{\mathbf{X}}, \hat{\hat{\mathbf{X}}}]$ is given in (S7.4). The derivations of $\frac{\partial}{\partial \hat{\mathbf{X}}}\hat{\beta}$ and $\frac{\partial}{\partial \hat{\hat{\mathbf{X}}}}\hat{\beta}$, with $\hat{\beta}$ given by (S2.15), are provided in § S7.

Remark S3. In case of large differences in state magnitudes, $\hat{\mathbf{V}}$ may alternatively be initialized as a diagonal matrix with elements inversely proportional to squared mean state values. Otherwise, initializing $\hat{\mathbf{V}} := \mathbf{I}_{TK}$ suffices as per Step 1.

Remark S4. We retained the $t_1, \ldots, t_T$ in this section for notational convenience. However, in case the measured time series are sampled too densely, it is worth considering a smaller set of measurement time points that still adequately capture the dynamics of the system. This choice offers a significant reduction in computational cost since GMGTS performs a large number of operations on large unstructured data matrices. Because the data are smoothed prior to gradient matching, the gradient matching time points can be freely chosen anywhere within the smoothing interval (cf. § S11).

Remark S5. It should be noted that estimates on the boundaries of the parameter space are generally undesirable. When this happens for some cells, we halt the FGLS iterations and omit the corresponding estimates from the second stage. In case a large fraction of cell-specific parameters are estimated at the boundary, we can either extend the search space or consider a suitable parameter prior, see § S9.

S2.4 GMGTS for partially observed systems

In the case of partial observation, the overall process of smoothing followed by gradient matching is preserved. However, since gradient matching requires full state information, we need to iteratively estimate the hidden states via ODE integration given the current estimate $\hat{\boldsymbol{\beta}}$, and update the estimate $\hat{\boldsymbol{\beta}}$ given the full state estimates and measurements (as in §S2.3, we omit cell subscripts i to simplify the notation). Without loss of generality, we assume that the first $L < K$ states are measured. Denoting the solution to (S2.6) by $\mathbf{F}(t; \hat{\boldsymbol{\beta}})$, we obtain predictions

$$\hat{\mathbf{x}}^H(t) = \mathbf{F}^H(t; \hat{\boldsymbol{\beta}}) \quad \text{and} \quad \hat{\dot{\mathbf{x}}}^H(t) = \mathbf{g}^H(\mathbf{F}(t; \hat{\boldsymbol{\beta}}))\hat{\boldsymbol{\beta}} + \mathbf{h}^H(\mathbf{F}(t; \hat{\boldsymbol{\beta}})), \tag{S2.17}$$

where $\mathbf{F}^H(t; \hat{\boldsymbol{\beta}})$ consists of the elements of $\mathbf{F}(t; \hat{\boldsymbol{\beta}})$ corresponding to the hidden states (similarly for $\mathbf{g}(\cdot)$ and $\mathbf{h}(\cdot)$). As before, we smooth the L observed states with B-splines to obtain $\hat{\mathbf{x}}^O(t)$. The hidden state predictions and smoothed state estimates can then be combined into full state and gradient estimates

$$\hat{\mathbf{x}}(t) = \left(\hat{\mathbf{x}}^O(t), \hat{\mathbf{x}}^H(t)\right) \quad \text{and} \quad \hat{\dot{\mathbf{x}}}(t) = \left(\hat{\dot{\mathbf{x}}}^O(t), \hat{\dot{\mathbf{x}}}^H(t)\right)$$

to perform gradient matching as in the fully observed case. However, the new parameter estimate $\hat{\boldsymbol{\beta}}$ will result in new hidden state predictions, so we need to iterate the prediction and gradient matching steps. The key steps of this iteration are described below. Similarly to §S2.3, we allow individual parameters to be constrained by bounds (S2.13).

1. Initialize $\hat{\boldsymbol{\beta}}$ at the same value $\hat{\boldsymbol{\beta}}^0$ for all cells (e.g., from prior knowledge or by fitting the average of the single-cell measurements, as described in §S11.3) and numerically integrate (S2.6) using $\hat{\boldsymbol{\beta}}^0$. The hidden state predictions are obtained directly from the numerical solution, and the gradient predictions for the hidden states are produced by substituting the solution into the right-hand side of the ODE. The uncertainty of the initial estimate $\hat{\boldsymbol{\beta}}^0$ needs to be prescribed, and should be taken large enough to allow subsequent estimates $\hat{\boldsymbol{\beta}}$ to move away from $\hat{\boldsymbol{\beta}}^0$ (see also §S11.3). With this uncertainty, initialize the error covariance matrix $\hat{\mathbf{V}}$ using (S8.4).
2. Given the full state estimate $\hat{\mathbf{x}}$ and the error covariance matrix $\hat{\mathbf{V}}$, update $\hat{\boldsymbol{\beta}}$ by minimizing (S2.14) as above.
3. Numerically integrate the system using the updated $\hat{\boldsymbol{\beta}}$ to obtain updated hidden state and gradient predictions; cf. (S2.17).
4. Update $\hat{\mathbf{V}}$ by propagating the uncertainty from the splines via $\hat{\boldsymbol{\beta}}$ to the hidden state components of $\hat{\mathbf{X}}$, $\hat{\dot{\mathbf{X}}}$, and subsequently to $\hat{\dot{\mathbf{X}}} - \mathbf{G}(\hat{\mathbf{X}})\hat{\boldsymbol{\beta}} - \mathbf{H}(\hat{\mathbf{X}})$ (see §S8).
5. Repeat Steps 2–4 until convergence (see §S11 for the implementation details of this scheme).

Since part of the system states are estimated using numerical ODE integration, Step 4 is now much more involved compared to the case of full observation since uncertainty feeds into the hidden states from the parameter estimate. As mentioned in Step 1, the uncertainty for the initial estimate $\hat{\boldsymbol{\beta}}^0$ is prescribed by the user. To propagate this uncertainty to the hidden state approximations, finite differences of the corresponding ODE solutions $\mathbf{F}^H(t; \hat{\boldsymbol{\beta}}^0)$ are the most time-efficient approach (see also §S11.3). Note the initial hidden state approximations are independent from the smoothing measurements. However, after the first gradient matching steps, the hidden and observed states become correlated through the updated parameter estimate $\hat{\boldsymbol{\beta}}$, which depends on the smoothing. Figure S3 shows how variables related to the iterative estimation of $\boldsymbol{\beta}$ are related, indicating how uncertainty propagates from $\hat{\boldsymbol{\beta}}^0$ and the smoothed measurements $\hat{\mathbf{x}}^O(t)$. As with full state information, the uncertainty $\mathbf{C}$ of $\hat{\boldsymbol{\beta}}$ should be approximated using (S2.16) once the iterations have converged. Note that in the partially observed case, this quantity is already calculated during the calculation of $\hat{\mathbf{V}}$ (see §S8).

Steps 2–4 constitute an iterative scheme that alternates between gradient matching and hidden state estimation to fill in the missing state information. As we detail in §S10, this scheme can be viewed as a fixed-point iteration. The convergence properties of this iteration are also discussed in §S10.

S3 Pre- and post-processing of dynamical systems

S3.1 Reformulation of the repressilator equations

To reformulate (5) into an ODE system with linear dependence in parameters, we consider a plausible set of elementary reactions that can produce the rational terms in (5) via a quasi-steady-state approximation. In these reactions, a monomer of repressor n_1 (r_{n_1}, where $n_1 = 1$, 2 or 3) forms a dimer (q_{n_1}) and binds to a free promoter of repressor n_2 (d_{n_2}) to form a DNA-protein complex (qd_{n_1,n_2}). Using a quasi-equilibrium approximation for the dimerization reaction, we can write the dimer concentration as a function of the total repressor concentration $p_n = r_n + 2q_n$. Concretely, using the dissociation constant K_d of the dimerization reaction and the law of mass action to write the equilibrium condition, we get

$$K_d q_n = (p_n - 2q_n)^2.$$

This equation can be solved in terms of q_n to find a function that connects q_n to the total concentration p_n. Setting $K_d = 4000$ µM, the physically meaningful solution of the quadratic equation is

$$q_n = g(p_n) := \tfrac{1}{2}p_n - \tfrac{1}{8}K_d\left(\sqrt{\frac{8p_n}{K_d} + 1} - 1\right) = \tfrac{1}{2}p_n - 500\left(\sqrt{p_n/500 + 1} - 1\right).$$

Finally, we rewrite (5) by explicitly modeling the binding of the dimer q_{n_2} to the promoter d_{n_1}, assuming that the rate constant of the binding reaction is equal to 1 µM^{-1}min^{-1} and the rate constant of the unbinding reaction is α_{n_1}:

$$\begin{aligned}\dot{qd}_{n_2,n_1}(t) &= g\left(p_{n_2}(t)\right)\left(d_{n_1}^{\text{total}} - qd_{n_2,n_1}(t)\right) - \alpha_{n_1} qd_{n_2,n_1}(t), \\ \dot{m}_{n_1}(t) &= 0.0005 + 0.5\left(1 - qd_{n_2,n_1}(t)/d_{n_1}^{\text{total}}\right) - 0.3466 m_{n_1}(t), \\ \dot{p}_{n_1}(t) &= 20 m_{n_1}(t) - 2g(p_{n_1}(t))\left(d_{n_3}^{\text{total}} - qd_{n_1,n_3}(t)\right) + 2\alpha_{n_3} qd_{n_1,n_3}(t) - \beta_{n_1} p_{n_1}(t),\end{aligned} \quad \text{for} \quad (n_1, n_2, n_3) = (1,3,2), (2,1,3), (3,2,1). \tag{S3.1}$$

In these equations, d_n^{total} is the total concentration of DNA that repressor n can bind. This concentration is set to 1.0µM for each repressor gene, which is much smaller than the concentration of the repressor itself, so that the dynamics of repressor concentration remains roughly the same. Note that the right-hand side of the system is nonlinear in each p_n but linear in each α_n, β_n. Since the complexes qd_{n_1,n_2} are generally not observed, we treat these states as hidden in the partially observed case.

The system (S3.1) can be used to infer random effects distributions of each α_n, β_n using GMGTS. To see how it is equivalent to (5), assume that the DNA-protein complexes reach a fast equilibrium. This means that the first ODE in (S3.1) is at equilibrium, and the DNA-repressor complex varies according to the total amount of repressor, implying

$$qd_{n_2,n_1}(t) = \frac{g(p_{n_2}(t)) d_{n_1}^{\text{total}}}{g(p_{n_2}(t)) + \alpha_{n_1}.} \tag{S3.2}$$

Using the fact that the dimer and monomer also reach a fast equilibrium, we can substitute the dimer abundance with the monomer squared divided by K_d. That is,

$$q_n(t) = \frac{r_n^2(t)}{K_d} = \frac{r_n^2(t)}{4000} = 0.00025 r_n^2(t). \tag{S3.3}$$

Substituting relations (S3.2) and (S3.3) into (S3.1), we get

$$\begin{aligned}\dot{m}_{n_1}(t) &= 0.0005 + \frac{0.5\alpha_{n_1}}{\alpha_{n_1} + g(p_{n_2}(t))} - 0.3466 m_{n_1}(t) = 0.0005 + \frac{0.5\alpha_{n_1}}{\alpha_{n_1} + 0.00025(r_{n_2}(t))^2} - 0.3466 m_{n_1}(t), \\ \dot{p}_{n_1}(t) &= 20 m_{n_1}(t) - 2g(p_{n_1}(t))\left(d_{n_3}^{\text{total}} - qd_{n_1,n_3}(t)\right) + 2\alpha_{n_3} qd_{n_1,n_3}(t) - \beta_{n_1} p_{n_1}(t),\end{aligned} \tag{S3.4}$$

Given the large dissociation constant K_d of the dimerization reaction and the range of repressor concentrations reached for the nominal parameter values, it is safe to assume that $r_{n_1} \approx p_{n_1}$ in (S3.4) to arrive at the differential equations for mRNA in (5). Furthermore, given the small concentration of DNA compared to the repressor concentrations, the middle terms in the protein equation have only a very small impact on the protein dynamics. Overall, (S3.4) produce mRNA and protein trajectories that are very close to those of the reduced system, as can be verified by simulation of the two systems. See Figure S1 for data simulated from both systems for a sample of sets of parameters.

S3.2 Bifunctional two-component system dynamics in terms of the total abundances of phosphorylated and free H and R

As discussed in [Kurdyaeva and Milias-Argeitis, 2021], the dynamics of the bifunctional two-component system is described by a six-dimensional set of ordinary differential equations. Omitting the time argument for clarity, these equations are

$$\begin{aligned}\dot{H} &= -k_1 H + k_4 H_p R - k_5 R_p \cdot H + (k_6 + k_7) R_p H + k_8 H_p, \\ \dot{H}_p &= k_1 H + k_3 H_p R - k_2 H_p \cdot R - k_8 H_p, \\ \dot{R} &= -k_2 H_p \cdot R + k_3 H_p R + k_7 R_p H, \\ \dot{R}_p &= -k_5 Rp \cdot H + k_4 H_p R + k_6 R_p H, \\ \dot{H_p R} &= k_2 H_p \cdot R - (k_3 + k_4) H_p R, \\ \dot{R_p H} &= k_5 R_p \cdot H - (k_6 + k_7) R_p H,\end{aligned} \tag{S3.5}$$

where H and H_p denote the unphosphorylated and phosphorylated histidine kinase respectively, and a similar convention is used for the response regulator R. $H_p R$ and $R_p H$ are the complexes formed by the binding of the kinase H_p to R and the phosphatase H to R_p. We will express (S3.5) in terms of quantities that can be observed experimentally, such as the total amounts of phosphorylated H and R, denoted by

$$H_p^{\text{total}} = H_p + H_p R \quad \text{and} \quad R_p^{\text{total}} = R_p + R_p H,$$

and the total amounts of free H and R, denoted

$$H^{\text{free}} = H + H_p = H + H_p^{\text{total}} - H_p R \quad \text{and} \quad R^{\text{free}} = R + R_p = R + R_p^{\text{total}} - R_p H. \tag{S3.6}$$

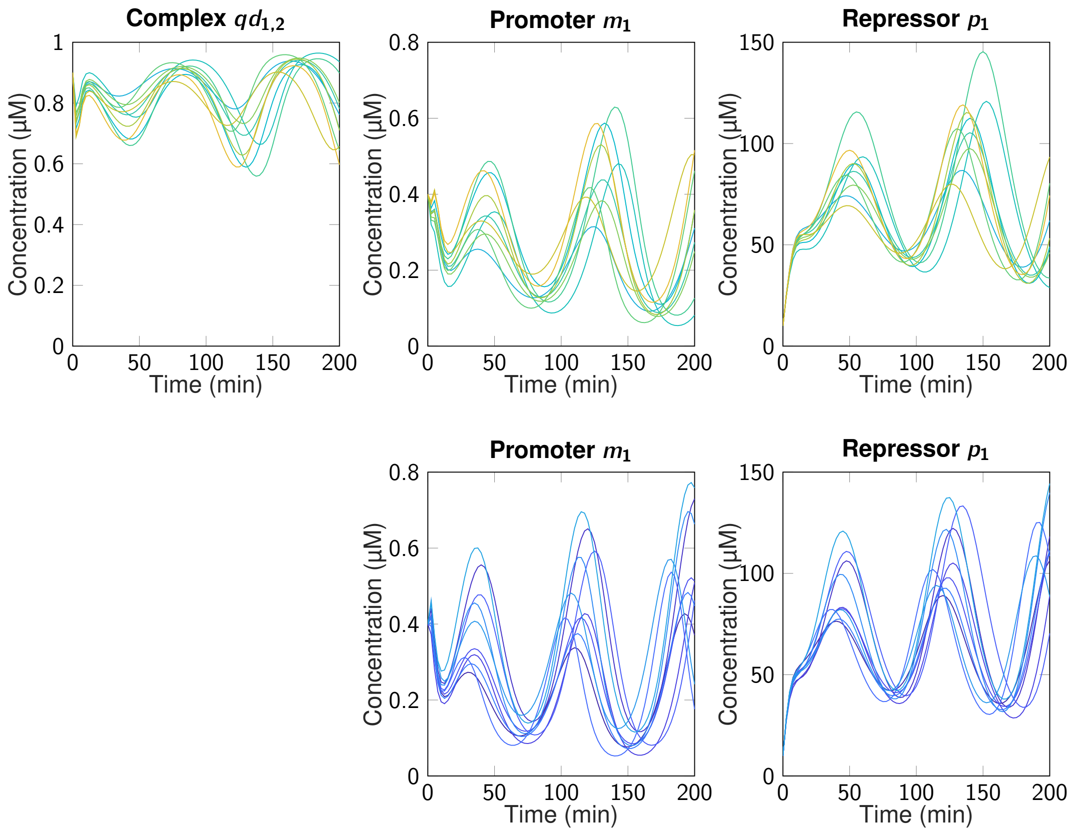

Figure S1: Repressilator trajectories obtained from the expanded and original system equations. The states that directly influence repressor p_1 are plotted for a sample of ten parameter sets α_n, β_n ($n = 1, 2, 3$) drawn from the random effects distribution described in Results. Similar trajectories are obtained for the remaining states. *Top panels:* Trajectories simulated from the repressilator system with explicitly modeled protein-DNA interactions (S3.1). *Bottom panels:* Trajectories simulated from the condensed repressilator system obtained from quasi-steady-state approximations and simplifying assumptions (5).

Using that $H_p = H_p^{\text{total}} - H_pR$ and $R_p = R_p^{\text{total}} - R_pH$, we can rewrite (S3.5) as

$$\begin{aligned}
\dot{H} &= -k_1 H + k_4 H_pR - k_5 (R_p^{\text{total}} - R_pH) \cdot H + (k_6 + k_7) R_pH + k_8 (H_p^{\text{total}} - H_pR), \\
\dot{R} &= -k_2 (H_p^{\text{total}} - H_pR) \cdot R + k_3 H_pR + k_7 R_pH, \\
\dot{H_pR} &= k_2 (H_p^{\text{total}} - H_pR) \cdot R - (k_3 + k_4) H_pR, \\
\dot{R_pH} &= k_5 (R_p^{\text{total}} - R_pH) \cdot H - (k_6 + k_7) R_pH, \\
\dot{H}_p^{\text{total}} &= k_1 H - k_8 (H_p^{\text{total}} - H_pR) - k_4 H_pR, \\
\dot{R}_p^{\text{total}} &= k_4 H_pR - k_7 R_pH.
\end{aligned} \tag{S3.7}$$

Using the relations (S3.6), we can further transform (S3.7) into

$$\begin{aligned}
\dot{H_pR} &= k_2 (H_p^{\text{total}} - H_pR) \cdot R - (k_3 + k_4) H_pR, \\
\dot{R_pH} &= k_5 (R_p^{\text{total}} - R_pH) \cdot H - (k_6 + k_7) R_pH, \\
\dot{H}^{\text{free}} &= (k_3 + k_4) H_pR + (k_6 + k_7) R_pH - k_2 (H_p^{\text{total}} - H_pR) \cdot R - k_5 (R_p^{\text{total}} - R_pH) \cdot H, \\
\dot{R}^{\text{free}} &= (k_3 + k_4) H_pR + (k_6 + k_7) R_pH - k_2 (H_p^{\text{total}} - H_pR) \cdot R - k_5 (R_p^{\text{total}} - R_pH) \cdot H, \\
\dot{H}_p^{\text{total}} &= k_1 H - k_8 (H_p^{\text{total}} - H_pR) - k_4 H_pR, \\
\dot{R}_p^{\text{total}} &= k_4 H_pR - k_7 R_pH.
\end{aligned} \tag{S3.8}$$

For the example we present in the Results of the main text, we assumed that the complexes H_pR and R_pH are hidden. To prevent a lack of identifiability, we also assumed that the reaction rate constants for one direction of each reaction were fixed, with the rate constant of the other direction being free.

S3.3 Reformulation of the delay-differential equations for one- and two-step fluorescent protein maturation with an ODE system

The kinetic models of one- and two-step fluorescent protein maturation used in [Guerra et al., 2022] are described in terms of delay differential equations to account for the delays in transcription and translation. To facilitate the application of gradient matching in

that case, the fluorescent protein dynamics in these models can be rewritten in terms of time-shifted ODE solutions of an unobserved state, effectively removing the delays from the reformulated system.

In the original models [Guerra et al., 2022], mRNA transcription is activated by an optogenetic system at time $t = 0$. mRNA (M) starts appearing τ minutes later, and undergoes degradation at a fixed rate per molecule. Dark (immature) fluorescent protein (D) is translated from mRNA with a time delay d, and eventually matures into fluorescent protein (F). All system components are diluted due to cell growth. These processes are described by the following set of delay differential equations:

$$\begin{aligned} \dot{M}(t) &= k_{\mathrm{r}} s(t-\tau) - (k_{\mathrm{dr}} + k_{\mathrm{dil}}) M(t), \\ \dot{D}(t) &= k_{\mathrm{p}} M(t-d) - (k_{\mathrm{m}} + k_{\mathrm{dil}}) D(t), \quad \text{for} \quad t \geqslant 0, \\ \dot{F}(t) &= k_{\mathrm{m}} D(t) - k_{\mathrm{dil}} F(t), \end{aligned} \tag{S3.9}$$

where $s : \mathbb{R} \to \{0, 1\}$ denotes the Heaviside step function. In the two-step maturation model, dark protein (D_1) goes through a second non-fluorescent form (D_2) before maturing into F. The analogous delay differential equations are

$$\begin{aligned} \dot{M}(t) &= k_{\mathrm{r}} s(t-\tau) - (k_{\mathrm{dr}} + k_{\mathrm{dil}}) M(t), \\ \dot{D}_1(t) &= k_{\mathrm{p}} M(t-d) - (k_{\mathrm{m1}} + k_{\mathrm{dil}}) D_1(t), \\ \dot{D}_2(t) &= k_{\mathrm{m1}} D_1(t) - (k_{\mathrm{m2}} + k_{\mathrm{dil}}) D_2(t), \\ \dot{F}(t) &= k_{\mathrm{m2}} D_2(t) - k_{\mathrm{dil}} F(t), \end{aligned} \quad \text{for} \quad t \geqslant 0. \tag{S3.10}$$

In either system, only the concentration of F can be measured by means of fluorescence microscopy, so we assume that states M and D (or D_1 and D_2) are unobserved. When F is measured, the transcriptional delay τ can easily be ignored by redefining the time axis of the system, so that time point zero of (S3.9) and (S3.10) coincides with time point τ of the measurements. The delay due to translation can also be removed, as detailed below.

Since the mRNA synthesis rate k_{r} and the dilution rate k_{dil} have already been fixed, additionally fixing the mRNA degradation rate k_{dr} effectively turns M into an input function to the dark protein equation. The solution of the mRNA differential equation with $M(0) = 0$ is readily verified to be

$$M(t) = \mathbb{1}_{[\tau,\infty)}(t) \frac{k_{\mathrm{r}}}{k_{\mathrm{dr}} + k_{\mathrm{dil}}} \left(1 - e^{-(k_{\mathrm{dr}}+k_{\mathrm{dil}})(t-\tau)}\right) \quad \text{for} \quad t \geqslant 0,$$

where $\mathbb{1}_A : \mathbb{R} \to \{0, 1\}$ denotes the indicator function on an interval A. We can then address the second time delay (in the dark protein equation) by substituting the delayed solution $M(t - d)$ into the differential equation for D, yielding (analogously for D_1)

$$\dot{D}(t) = \mathbb{1}_{[\tau+d,\infty)}(t) \frac{k_{\mathrm{p}} k_{\mathrm{r}}}{k_{\mathrm{dr}} + k_{\mathrm{dil}}} \left(1 - e^{-(k_{\mathrm{dr}}+k_{\mathrm{dil}})(t-\tau-d)}\right) - (k_{\mathrm{m}} + k_{\mathrm{dil}}) D(t) \quad \text{for} \quad t \geqslant 0.$$

However, because τ and d only appear in the input function $M(t - d)$, they vanish when we redefine the time axis in terms of $u := t - \tau - d$. Note this operation of shifting the time axis means that time point zero of (S3.11) and (S3.12) corresponds with time point $\tau + d$ of the measurements. Accordingly, we obtain a delay-free two-dimensional system

$$\begin{aligned} \dot{D}(u) &= \frac{k_{\mathrm{p}} k_{\mathrm{r}}}{k_{\mathrm{dr}} + k_{\mathrm{dil}}} \left(1 - e^{-(k_{\mathrm{dr}}+k_{\mathrm{dil}})u}\right) - (k_{\mathrm{m}} + k_{\mathrm{dil}}) D(u), \\ \dot{F}(u) &= k_{\mathrm{m}} D(u) - k_{\mathrm{dil}} F(u), \end{aligned} \quad \text{for} \quad u \geqslant 0 \tag{S3.11}$$

for the one-step maturation model, and a three-dimensional system

$$\begin{aligned} \dot{D}_1(u) &= \frac{k_{\mathrm{p}} k_{\mathrm{r}}}{k_{\mathrm{dr}} + k_{\mathrm{dil}}} \left(1 - e^{-(k_{\mathrm{dr}}+k_{\mathrm{dil}})u}\right) - (k_{\mathrm{m1}} + k_{\mathrm{dil}}) D_1(u), \\ \dot{D}_2(u) &= k_{\mathrm{m1}} D_1(u) - (k_{\mathrm{m2}} + k_{\mathrm{dil}}) D_2(u), \\ \dot{F}(u) &= k_{\mathrm{m2}} D_2(u) - k_{\mathrm{dil}} F(u), \end{aligned} \quad \text{for} \quad u \geqslant 0 \tag{S3.12}$$

for the two-step maturation model.

S3.4 Preprocessing of experimental data obtained through fluorescence microscopy

Prior to inference, the measurements for each FP need to be preprocessed. First, the average fluorescence intensity of a mother cell was used as a proxy of the FP concentration in that cell [Guerra et al., 2022] (assuming that concentration of the FP in the mother and in the bud is the same). By the nature of the transcription and translation processes, mRNA synthesis starts with a delay of two minutes [Rullan et al., 2018], and translation is delayed by another four minutes [Guerra et al., 2022]. Given the reformulated systems described above, this delay is now realized by shifting the measurements by six minutes (see (S3.11) and (S3.12)). Since the measurements are taken every five minutes, the six-minute shift is obtained by linear interpolation. Subsequently, in the new shifted time grid, only times $u = 0, 5, 10, \ldots, 100$ minutes are considered for the one-step model ($u = 0, 5, \ldots, 200$ minutes for the two-step model), with u defined as in §S3.3.

Since the fluorescence measurements at the start of the experiment are dominated by cellular autofluorescence, the corresponding fluorescence time series were shifted downwards in order to start at zero. Any measurements that became negative as a result of this modification due to measurement noise were truncated at zero. Cells with missing or too few measurements were omitted from the parameter inference problem.

S3.5 Approximation of an equivalent maturation half-time for proteins undergoing two-step maturation

For proteins with a single rate-limiting maturation step, the maturation half-time $\mathrm{t}_{1/2}$ and the maturation rate are related through $\mathrm{t}_{1/2} = \log(2)k_\mathrm{m}^{-1} \approx 0.693k_\mathrm{m}^{-1}$. The relationship between maturation rates and the maturation half-time is less straightforward for proteins with two rate-limiting maturation steps, as the half-times of the individual steps only form a lower bound for the actual maturation half-time of the protein [Guerra et al., 2022]. To approximate this half-time analytically, we consider the solution to the initial value problem

$$\begin{aligned} \dot{D}_1(t) &= -k_{\mathrm{m}1}D_1(t), \\ \dot{D}_2(t) &= k_{\mathrm{m}1}D_1(t) - k_{\mathrm{m}2}D_2(t), \qquad D_1(0) = 1, \qquad D_2(0) = F(0) = 0, \\ \dot{F}(t) &= k_{\mathrm{m}2}D_2(t), \end{aligned} \tag{S3.13}$$

and find the time $\mathrm{t}_{1/2}$ at which $F(\mathrm{t}_{1/2}) = 0.5$. As we have previously shown [Guerra et al., 2022], independent inference of $k_{\mathrm{m}1}$ and $k_{\mathrm{m}2}$ is not possible when only measurements of $F(t)$ are available, because $k_{\mathrm{m}1}$ and $k_{\mathrm{m}2}$ are practically non-identifiable. To overcome this problem, we assume that $k_\mathrm{m} := k_{\mathrm{m}1} = k_{\mathrm{m}2}$. Under this assumption, the solution the solution $F(t)$ to (S3.13) is

$$F(t) = 1 - e^{-tk_\mathrm{m}}(1 + tk_\mathrm{m}) \quad \text{for} \quad t \geqslant 0.$$

Therefore, $\mathrm{t}_{1/2}$ solves

$$-\frac{1}{2e} = -(1 + \mathrm{t}_{1/2}k_\mathrm{m})e^{-(1+\mathrm{t}_{1/2}k_\mathrm{m})},$$

implying

$$\mathrm{t}_{1/2} = k_\mathrm{m}^{-1}\left(-W_{-1}\left(-\frac{1}{2e}\right) - 1\right) \approx 1.678k_\mathrm{m}^{-1}, \tag{S3.14}$$

where $W_{-1} : [-1/e, 0) \to \mathbb{R}$ denotes the real part of the the (-1)-branch of the Lambert W function [Corless et al., 1996]. This relation provides a straightforward connection between the maturation half-time and the maturation rates of the two-step model.

S3.6 The distribution of maturation half-times

When β follows a multivariate log-normal distribution $\mathrm{LN}(\mathbf{b}, \mathbf{D})$, its p-th component β_p follows a one-dimensional log-normal distribution with mean b_p and variance D_{pp}, the respective elements of $\mathbf{b}$ and $\mathbf{D}$. When β_p represents the FP maturation rate, the log-mean of maturation half-time $t_{1/2}$ is proportional to β_p^{-1}.

To find the distribution of $t_{1/2}$, we note that when $\beta_p \sim \mathrm{LN}(b_p, D_{pp})$, then $\beta_p^{-1} \sim \mathrm{LN}(-b_p, D_{pp})$. Moreover, it is easy to verify that multiplication of β_p^{-1} by a constant results in a shifted log-normal distribution: $c \cdot \beta_p^{-1} \sim \mathrm{LN}(-b_p + \log c, D_{pp})$. As such, in case of single-step maturation,

$$t_{1/2} \approx 0.693\beta_p^{-1} \sim \mathrm{LN}(-b_p + \log 0.693, D_{pp}),$$

while for two-step maturation,

$$t_{1/2} \approx 1.678\beta_p^{-1} \sim \mathrm{LN}(-b_p + \log 1.678, D_{pp});$$

cf. (S3.14).

S4 Derivation of the second-stage EM algorithm

In the second step of the GTS method (§ S2.2), an EM algorithm is used to estimate the population parameters $\mathbf{b}$ and $\mathbf{D}$, treating the first-stage individual estimates $\hat{\beta}_i$ as data and the unknown β_i as latent variables. Though this EM algorithm has been frequently mentioned in the NLME literature over the years, we have been unable to find the calculations behind the EM steps outlined in § S2.2. We therefore provide a derivation of these steps below.

The EM algorithm starts with the complete-data likelihood function of $\mathbf{b}, \mathbf{D}$, denoted $p(\beta_1, \ldots, \beta_N, \hat{\beta}_1, \ldots, \hat{\beta}_N \mid \mathbf{b}, \mathbf{D})$. In the E-step, we compute the expected value $q(\mathbf{b}, \mathbf{D} \mid \hat{\mathbf{b}}, \hat{\mathbf{D}})$ of the complete-data log-likelihood with respect to the conditional distribution of $\beta_1, \ldots, \beta_N$ given $\hat{\beta}_1, \ldots, \hat{\beta}_N$ and the current estimates $\hat{\mathbf{b}}, \hat{\mathbf{D}}$. Symbolically,

$$q(\mathbf{b}, \mathbf{D} \mid \hat{\mathbf{b}}, \hat{\mathbf{D}}) = \mathbb{E}_{\beta_1,\ldots,\beta_N \mid \hat{\beta}_1,\ldots,\hat{\beta}_N, \hat{\mathbf{b}}, \hat{\mathbf{D}}}\left\{\log p(\beta_1, \ldots, \beta_N, \hat{\beta}_1, \ldots, \hat{\beta}_N \mid \mathbf{b}, \mathbf{D})\right\} = \sum_{i=1}^{N} \mathbb{E}_{\beta_i \mid \hat{\beta}_i, \hat{\mathbf{b}}, \hat{\mathbf{D}}}\left\{\log p(\beta_i, \hat{\beta}_i \mid \mathbf{b}, \mathbf{D})\right\},$$

where we used the conditional independence of individual estimates to factorize the likelihood.

At the M-step, we maximize $q(\mathbf{b}, \mathbf{D} \mid \hat{\mathbf{b}}, \hat{\mathbf{D}})$ with respect to $\mathbf{b}$ and $\mathbf{D}$ to obtain updated estimates $\hat{\mathbf{b}}^{\mathrm{new}}, \hat{\mathbf{D}}^{\mathrm{new}}$. That is,

$$\left(\hat{\mathbf{b}}^{\mathrm{new}}, \hat{\mathbf{D}}^{\mathrm{new}}\right) := \arg\max_{\mathbf{b},\mathbf{D}} q(\mathbf{b}, \mathbf{D} \mid \hat{\mathbf{b}}, \hat{\mathbf{D}}).$$

To derive the conditional distribution of each β_i given $\hat{\beta}_i$, we use Bayes' theorem together with (S2.9) to write

$$\begin{aligned} \log p(\beta_i \mid \hat{\beta}_i, \hat{\mathbf{b}}, \hat{\mathbf{D}}) &\propto \log p(\hat{\beta}_i \mid \beta_i, \hat{\mathbf{b}}, \hat{\mathbf{D}}) + \log p(\beta_i \mid \hat{\mathbf{b}}, \hat{\mathbf{D}}) - \log p(\hat{\beta}_i \mid \hat{\mathbf{b}}, \hat{\mathbf{D}}) \\ &\propto -\tfrac{1}{2}\left(\hat{\beta}_i - \beta_i\right)' \mathbf{C}_i^{-1}\left(\hat{\beta}_i - \beta_i\right) - \tfrac{1}{2}\left(\beta_i - \hat{\mathbf{b}}\right)' \hat{\mathbf{D}}^{-1}\left(\beta_i - \hat{\mathbf{b}}\right) + \tfrac{1}{2}\left(\hat{\beta}_i - \hat{\mathbf{b}}\right)' \left(\hat{\mathbf{D}}^{-1} + \mathbf{C}_i^{-1}\right)^{-1}\left(\hat{\beta}_i - \hat{\mathbf{b}}\right) \end{aligned}$$

$$\propto -\tfrac{1}{2}\beta_i'\left(\hat{\mathbf{D}}^{-1}+\mathbf{C}_i^{-1}\right)\beta_i+\beta_i'\left(\hat{\mathbf{D}}^{-1}\hat{\mathbf{b}}+\mathbf{C}_i^{-1}\hat{\beta}_i\right)$$
$$\propto -\tfrac{1}{2}\left(\beta_i-\left(\hat{\mathbf{D}}^{-1}+\mathbf{C}_i^{-1}\right)^{-1}\left(\hat{\mathbf{D}}^{-1}\hat{\mathbf{b}}+\mathbf{C}_i^{-1}\hat{\beta}_i\right)\right)'\left(\hat{\mathbf{D}}^{-1}+\mathbf{C}_i^{-1}\right)\left(\beta_i-\left(\hat{\mathbf{D}}^{-1}+\mathbf{C}_i^{-1}\right)^{-1}\left(\hat{\mathbf{D}}^{-1}\hat{\mathbf{b}}+\mathbf{C}_i^{-1}\hat{\beta}_i\right)\right).$$

Therefore, the conditional distribution of β_i given $\hat{\beta}_i$ is normal with

$$\mathbb{E}\left\{\beta_i \mid \hat{\beta}_i,\hat{\mathbf{b}},\hat{\mathbf{D}}\right\}=\left(\hat{\mathbf{D}}^{-1}+\mathbf{C}_i^{-1}\right)^{-1}\left(\hat{\mathbf{D}}^{-1}\hat{\mathbf{b}}+\mathbf{C}_i^{-1}\hat{\beta}_i\right)=:\tilde{\beta}_i \quad\text{and}\quad \operatorname{Var}\left\{\beta_i \mid \hat{\beta}_i,\hat{\mathbf{b}},\hat{\mathbf{D}}\right\}=\left(\hat{\mathbf{D}}^{-1}+\mathbf{C}_i^{-1}\right)^{-1}.$$

To calculate the expectation of the complete-data log-likelihood with respect to this conditional distribution, we make use of the fact that, for a normally distributed random vector $\mathbf{z}$ and a symmetric matrix $\mathbf{A}$ of conforming dimension, $\mathbb{E}\{\mathbf{z}'\mathbf{A}\mathbf{z}\}=\mathbb{E}\{\mathbf{z}'\}\mathbf{A}\,\mathbb{E}\{\mathbf{z}\}+\operatorname{tr}(\mathbf{A}\operatorname{Var}\mathbf{z})$ [Kendrick, 2002, App. B.1]. Therefore, we obtain

$$\mathbb{E}_{\beta_i|\hat{\beta}_i,\hat{\mathbf{b}},\hat{\mathbf{D}}}\left\{\log p(\beta_i,\hat{\beta}_i \mid \mathbf{b},\mathbf{D})\right\}\propto \mathbb{E}_{\beta_i|\hat{\beta}_i,\hat{\mathbf{b}},\hat{\mathbf{D}}}\left\{-\tfrac{1}{2}\left(\hat{\beta}_i-\beta_i\right)'\mathbf{C}_i^{-1}\left(\hat{\beta}_i-\beta_i\right)-\tfrac{1}{2}\left(\beta_i-\mathbf{b}\right)'\mathbf{D}^{-1}\left(\beta_i-\mathbf{b}\right)-\tfrac{1}{2}\log|\mathbf{D}|\right\}$$
$$\propto -\tfrac{1}{2}\left(\tilde{\beta}_i-\mathbf{b}\right)'\mathbf{D}^{-1}\left(\tilde{\beta}_i-\mathbf{b}\right)-\tfrac{1}{2}\operatorname{tr}\left(\mathbf{D}^{-1}\left(\hat{\mathbf{D}}^{-1}+\mathbf{C}_i^{-1}\right)^{-1}+\tfrac{1}{2}\log\left|\mathbf{D}^{-1}\right|\right).$$

Consequently,

$$q(\mathbf{b},\mathbf{D}\mid\hat{\mathbf{b}},\hat{\mathbf{D}})\propto-\tfrac{1}{2}\sum_{i=1}^{N}\left[\left(\tilde{\beta}_i-\mathbf{b}\right)'\mathbf{D}^{-1}\left(\tilde{\beta}_i-\mathbf{b}\right)+\operatorname{tr}\left(\mathbf{D}^{-1}\left(\hat{\mathbf{D}}^{-1}+\mathbf{C}_i^{-1}\right)^{-1}-\log\left|\mathbf{D}^{-1}\right|\right)\right].$$

To derive the M-step update, note $q(\mathbf{b},\mathbf{D}\mid\hat{\mathbf{b}},\hat{\mathbf{D}})$ has partial derivatives [Petersen and Pedersen, 2008, Ch. 2]

$$\tfrac{\partial}{\partial\mathbf{b}}q(\mathbf{b},\mathbf{D}\mid\hat{\mathbf{b}},\hat{\mathbf{D}})=-N\mathbf{D}^{-1}\mathbf{b}+\sum_{i=1}^{N}\mathbf{D}^{-1}\tilde{\beta}_i \quad\text{and}\quad \tfrac{\partial}{\partial\mathbf{D}^{-1}}q(\mathbf{b},\mathbf{D}\mid\hat{\mathbf{b}},\hat{\mathbf{D}})=-\tfrac{1}{2}\sum_{i=1}^{N}\left(\left(\tilde{\beta}_i-\mathbf{b}\right)\left(\tilde{\beta}_i-\mathbf{b}\right)'+\left(\hat{\mathbf{D}}^{-1}+\mathbf{C}_i^{-1}\right)^{-1}\right)+\tfrac{1}{2}N\mathbf{D}.$$

The first-order conditions $\frac{\partial}{\partial\mathbf{b}}q(\mathbf{b},\mathbf{D}\mid\hat{\mathbf{b}},\hat{\mathbf{D}})=\frac{\partial}{\partial\mathbf{D}^{-1}}q(\mathbf{b},\mathbf{D}\mid\hat{\mathbf{b}},\hat{\mathbf{D}})=0$ imply

$$\hat{\mathbf{b}}^{\text{new}}:=\frac{1}{N}\sum_{i=1}^{N}\tilde{\beta}_i \quad\text{and}\quad \hat{\mathbf{D}}^{\text{new}}:=\frac{1}{N}\sum_{i=1}^{N}\left(\left(\hat{\mathbf{D}}^{-1}+\mathbf{C}_i^{-1}\right)^{-1}+\left(\tilde{\beta}_i-\mathbf{b}\right)\left(\tilde{\beta}_i-\mathbf{b}\right)'\right),$$

and the second-order conditions are readily verified to confirm $\hat{\mathbf{b}}^{\text{new}}$ and $\hat{\mathbf{D}}^{\text{new}}$ maximize $q(\mathbf{b},\mathbf{D}\mid\hat{\mathbf{b}},\hat{\mathbf{D}})$ with respect to $\mathbf{b}$ and $\mathbf{D}$.

S5 Inference of log-normal random effects distributions

While the first stage of (GM)GTS is distribution-agnostic, the most commonly made assumption in the second stage is that the random effects distribution is a multivariate normal. Under this assumption, the parameters of the random effects distribution can be inferred with the help of the EM scheme described in § S4.

The normality assumption implies that individual parameters can take negative values. Even if this may happen with extremely small probability, negative values are often not permissible in models of biological systems. A common strategy for ensuring that individual parameters remain positive in GTS is to perform the first-stage inference in the log-parameter space and assume that the parameter logarithms follow a normal distribution in the second stage. Effectively, this approach amounts to inferring a log-normal random effects distribution in the second GTS stage. However, the system that is inferred in the first stage is no longer linear in parameters, which implies we cannot make use of linear regression in GMGTS.

In principle, inference in the log-space is not necessary in the first GTS stage, as one can always infer a log-normal random effects distribution in the second stage, making use of the first-stage uncertainties. However, this deconvolution problem is much harder to solve compared to the case of normal random effects. To infer log-normal random effects distributions without losing the linearity in parameters in the first stage of GMGTS, we developed two alternative approaches, each based on a different type of approximation.

S5.1 Alternative 1: approximation of first-stage normal distributions by log-normal ones

The generalized least squares estimator (S2.15) at the heart of the first GMGTS stage produces asymptotically normal individual parameter estimates $\hat{\beta}_i$, $i=1,\ldots,N$ whose covariance matrix $\mathbf{C}_i=\operatorname{Var}\hat{\beta}_i$, $i=1,\ldots,N$ is given by (S2.16). This normal distribution can be approximated by a log-normal, provided the individual estimates are not too close to zero and their variances are not too large. To simplify the notation, we will again omit the cell index i in the following.

There are several ways to produce a multivariate log-normal distribution $\operatorname{LN}(\mathbf{m},\mathbf{S})$ that matches the normal $\operatorname{N}(\hat{\beta},\mathbf{C})$. Minimization of the Kullback-Leibler divergence is a principled choice, but it quickly becomes computationally cumbersome as the dimensionality of $\hat{\beta}$ increases. Matching the first and second moments of the two distributions is another option, which leads to analytically tractable approximations. In our experience, matching the marginal medians and covariances leads to a log-normal approximation that best captures the central tendency of the normal distribution.

Concretely, this approximation amounts to setting

$$m_p=\log\hat{\beta}_p \quad\text{for}\quad p=1,\ldots,P, \tag{S5.1}$$

where m_p and $\hat{\beta}_p$ denote the p-th components of $\mathbf{m}$ and $\hat{\beta}$, and where P is the dimension of $\hat{\beta}$. To match the covariance matrices of the $\mathrm{LN}(\mathbf{m}, \mathbf{S})$ and $\mathrm{N}(\hat{\beta}, \mathbf{C})$ distributions, we equate

$$C_{pr} = e^{m_p+m_r+\frac{1}{2}(S_{pp}+S_{rr})}\left(e^{S_{pr}}-1\right) = \hat{\beta}_p\hat{\beta}_r e^{\frac{1}{2}(S_{pp}+S_{rr})}\left(e^{S_{pr}}-1\right) \quad \text{for} \quad p, r = 1, \ldots, P, \tag{S5.2}$$

where C_{pr} and S_{pr} denote the p, r-th elements of $\mathbf{C}$ and $\mathbf{S}$, respectively. Setting $p = r$ in (S5.2), we can obtain the diagonal elements of $\mathbf{S}$ by noting that

$$C_{pp} = \hat{\beta}_p^2 \cdot e^{S_{pp}}\left(e^{S_{pp}}-1\right) \quad \text{for} \quad p = 1, \ldots, P,$$

which results in quadratic equations for $u := \mathrm{e}^{S_{pp}}$:

$$\hat{\beta}_p^2 u^2 - \hat{\beta}_p^2 u - C_{pp} = 0 \quad \text{for} \quad p = 1, \ldots, P.$$

Solving this equation for u yields one positive solution

$$u = \frac{\hat{\beta}_p^2 + \sqrt{\hat{\beta}_p^4 + 4\hat{\beta}^2 C_{pp}}}{2\hat{\beta}_p^2},$$

and therefore

$$S_{pp} = \log\left(\frac{1}{2} + \sqrt{\frac{1}{4} + \frac{C_{pp}}{\hat{\beta}_p^2}}\right). \tag{S5.3}$$

Substituting this expression back in (S5.2), we can calculate the off-diagonal elements S_{pr} in terms of $\hat{\beta}$ and the diagonal of $\mathbf{S}$:

$$S_{pr} = \log\left(1 + C_{pr}(\hat{\beta}_p\hat{\beta}_r)^{-1}e^{-\frac{1}{2}(S_{pp}+S_{rr})}\right). \tag{S5.4}$$

Equations (S5.1), (S5.3), and (S5.4) completely specify the approximating log-normal distribution.

With the individual estimates approximately following a $\mathrm{LN}(\mathbf{m}, \mathbf{S})$ distribution, the original EM scheme of the GTS method can now be applied to the logarithms of these parameters, which follow a normal distribution with mean vector $\mathbf{m}$ and covariance matrix $\mathbf{S}$. The EM scheme will produce a normal random effects distribution for the parameter logarithms, which is equivalent to a log-normal distribution for the parameters themselves.

S5.2 Alternative 2: approximation of the second-stage marginal likelihood

An alternative to approximating the normally distributed parameter uncertainties of the first stage is to approximate the marginal likelihood of the second stage. The full probabilistic model in that case will look as follows: individual cell parameters β_i, $i = 1, \ldots, N$, follow a log-normal distribution $\mathrm{LN}(\mathbf{b}, \mathbf{D})$, where $\mathbf{b}$ is the log-mean and $\mathbf{D}$ the log-covariance matrix. These individual parameters are estimated in the first stage of the GMGTS method assumed having normally distributed additive error; that is, $\hat{\beta}_i = \beta_i + \epsilon_i$, $i = 1, \ldots, N$, where ϵ_i follows a normal distribution with zero mean and covariance matrix $\mathbf{C}_i$.

Given $\mathbf{b}$ and $\mathbf{D}$, we will denote the likelihood of observing $\hat{\beta}_i$, $i = 1, \ldots, N$, by $p(\hat{\beta}_1, \ldots, \hat{\beta}_N \mid \mathbf{b}, \mathbf{D})$. This is obtained by the following expression:

$$p(\hat{\beta}_1, \ldots, \hat{\beta}_N \mid \mathbf{b}, \mathbf{D}) = \prod_{i=1}^{N} \int p(\hat{\beta}_i \mid \beta_i)\, p(\beta_i \mid \mathbf{b}, \mathbf{D})\; d\beta_i, \tag{S5.5}$$

where $p(\hat{\beta}_i \mid \beta_i)$ is a normal density and $p(\beta_i \mid \mathbf{b}, \mathbf{D})$ is a log-normal. To facilitate the approximation, we first perform a change of variables by setting $\mathbf{u}_i := \log \beta_i$:

$$p(\hat{\beta}_1, \ldots, \hat{\beta}_N \mid \mathbf{b}, \mathbf{D}) = \prod_{i=1}^{N} \int p(\hat{\beta}_i \mid \mathrm{e}^{\mathbf{u}_i})\, p(\mathrm{e}^{\mathbf{u}_i} \mid \mathbf{b}, \mathbf{D}) \left(\prod_{p=1}^{P} \beta_{i,p}\right) d\mathbf{u}_i. \tag{S5.6}$$

As a log-normal density, $p(\beta_i \mid \mathbf{b}, \mathbf{D})$ has the form

$$p(\beta_i \mid \mathbf{b}, \mathbf{D}) = (2\pi)^{-P/2}|\mathbf{D}|^{-1/2}\left(\prod_{p=1}^{P} \beta_{i,p}\right)^{-1} \exp\left(-\frac{1}{2}(\log\beta_i - \mathbf{b})'\, \mathbf{D}^{-1}\, (\log\beta_i - \mathbf{b})\right).$$

Plugging this expression in (S5.6) and taking into account the form of the normal density $p(\hat{\beta}_i \mid \mathrm{e}^{\mathbf{u}_i})$, we obtain

$$p(\hat{\beta}_1, \ldots, \hat{\beta}_N \mid \mathbf{b}, \mathbf{D}) = \prod_{i=1}^{N} \int (2\pi)^{-P}|\mathbf{C}_i|^{-1/2}|\mathbf{D}|^{-1/2} \exp\left(\left(\hat{\beta}_i - \mathrm{e}^{\mathbf{u}_i}\right)' \mathbf{C}_i^{-1}\left(\hat{\beta}_i - \mathrm{e}^{\mathbf{u}_i}\right)\right) \exp\left(-\frac{1}{2}(\mathbf{u}_i - \mathbf{b})'\, \mathbf{D}^{-1}\, (\mathbf{u}_i - \mathbf{b})\right) du_i. \tag{S5.7}$$

Exact evaluation of the integrals in (S5.7) is still not possible, but each exponential term

$$f(u_i) = \exp\left(\left(\hat{\beta}_i - \mathrm{e}^{\mathbf{u}_i}\right)' \mathbf{C}_i^{-1}\left(\hat{\beta}_i - \mathrm{e}^{\mathbf{u}_i}\right)\right)$$

can be approximated by a Gaussian function centered at $\mathbf{u}_i^* = \log \hat{\boldsymbol{\beta}}_i$, the location of the maximum of $f(\mathbf{u}_i)$. This approach is reminiscent of the Laplace approximation and should provide a good approximation of $f(\mathbf{u}_i)$ when the magnitude of the covariance matrix $\mathbf{C}_i$ is not too large.

Approximation of $f(\mathbf{u}_i)$ by a Gaussian amounts to approximating $\log f(\mathbf{u}_i)$ by a quadratic function, for which we need the Hessian of $f(\mathbf{u}_i)$ at $\mathbf{u}_i^*$. Starting from the gradient of $f(\mathbf{u}_i)$, we obtain

$$\frac{\partial f}{\partial \mathbf{u}_i} = \begin{bmatrix} \mathrm{e}^{u_{i,1}} & & \\ & \ddots & \\ & & \mathrm{e}^{u_{i,P}} \end{bmatrix} \mathbf{C}_i^{-1} \left(\hat{\boldsymbol{\beta}}_i - \mathrm{e}^{\mathbf{u}_i} \right).$$

Denoting the Hessian of $f(\mathbf{u}_i)$ at $\mathbf{u}_i^*$ by $H_f(\mathbf{u}_i^*)$, we obtain the diagonal matrix

$$H_f(\mathbf{u}_i^*) = \begin{bmatrix} \mathrm{e}^{u_{i,1}^*} & & \\ & \ddots & \\ & & \mathrm{e}^{u_{i,P}^*} \end{bmatrix} \operatorname{diag}\left(\mathbf{C}_i^{-1} \right) \begin{bmatrix} \hat{\beta}_{i,1} - 2\mathrm{e}^{u_{i,1}^*} & & \\ & \ddots & \\ & & \hat{\beta}_{i,P} - 2\mathrm{e}^{u_{i,P}^*} \end{bmatrix}.$$

The Gaussian approximation of $f(\mathbf{u}_i)$ centered at $\mathbf{u}_i^*$ therefore becomes

$$f_G(\mathbf{u}_i) = \exp\left(\frac{1}{2} \left(\mathbf{u}_i - \mathbf{u}_i^* \right)' H_f(\mathbf{u}_i^*) \left(\mathbf{u}_i - \mathbf{u}_i^* \right) \right) \tag{S5.8}$$

since the gradient of $f(\mathbf{u}_i)$ is zero at $\mathbf{u}_i^*$. Plugging (S5.8) into (S5.7), the individual terms of the product become approximately equal to integrals of products of Gaussians:

$$p(\hat{\boldsymbol{\beta}}_1, \ldots, \hat{\boldsymbol{\beta}}_N \mid \mathbf{b}, \mathbf{D}) \approx \prod_{i=1}^{N} \int (2\pi)^{-P} |\mathbf{C}_i|^{1/2} |\mathbf{D}|^{-1/2} \exp\left(\frac{1}{2} \left(\mathbf{u}_i - \mathbf{u}_i^* \right)' H_f(\mathbf{u}_i^*) \left(\mathbf{u}_i - \mathbf{u}_i^* \right) \right) \exp\left(-\frac{1}{2} \left(\mathbf{u}_i - \mathbf{b} \right)' \mathbf{D}^{-1} \left(\mathbf{u}_i - \mathbf{b} \right) \right) d\mathbf{u}_i. \tag{S5.9}$$

Dropping the multiplicative factor $(2\pi)^{-P} |\mathbf{C}_i|^{-1/2}$, which does not depend on $\mathbf{b}$ or $\mathbf{D}$, the marginal likelihood (S5.7) becomes approximately proportional to

$$L(\mathbf{b}, \mathbf{D}) = \prod_{i=1}^{N} \int |\mathbf{D}|^{-1/2} \exp\left(\frac{1}{2} \left(\mathbf{u}_i - \mathbf{u}_i^* \right)' H_f(\mathbf{u}_i^*) \left(\mathbf{u}_i - \mathbf{u}_i^* \right) \right) \exp\left(-\frac{1}{2} \left(\mathbf{u}_i - \mathbf{b} \right)' \mathbf{D}^{-1} \left(\mathbf{u}_i - \mathbf{b} \right) \right) d\mathbf{u}_i. \tag{S5.10}$$

Denoting

$$\boldsymbol{\Lambda}_i := \left(-H_f(u_i^*) + \mathbf{D}^{-1} \right)^{-1}$$

and

$$\boldsymbol{\mu}_i := \boldsymbol{\Lambda}_i \left(-H_f(\mathbf{u}_i^*) \mathbf{u}_i^* + \mathbf{D}^{-1} \mathbf{b} \right),$$

(S5.10) becomes

$$L(\mathbf{b}, \mathbf{D}) = \prod_{i=1}^{N} \int |\mathbf{D}|^{-1/2} \exp\left(-\frac{1}{2} \left(\mathbf{u}_i - \boldsymbol{\mu}_i \right)' \boldsymbol{\Lambda}_i^{-1} \left(\mathbf{u}_i - \boldsymbol{\mu}_i \right) \right) \exp\left(\frac{1}{2} \left[(\mathbf{u}_i^*)' H_f(\mathbf{u}_i^*) \mathbf{u}_i^* - \mathbf{b}' \mathbf{D}^{-1} \mathbf{b} + \boldsymbol{\mu}_i' \boldsymbol{\Lambda}_i^{-1} \boldsymbol{\mu}_i \right] \right) d\mathbf{u}_i. \tag{S5.11}$$

Since

$$\int \exp\left(-\frac{1}{2} \left(\mathbf{u}_i - \boldsymbol{\mu}_i \right)' \boldsymbol{\Lambda}_i^{-1} \left(\mathbf{u}_i - \boldsymbol{\mu}_i \right) \right) d\mathbf{u}_i = (2\pi)^{P/2} |\boldsymbol{\Lambda}_i|^{1/2},$$

each integral in (S5.11) is equal to

$$(2\pi)^{P/2} \frac{|\boldsymbol{\Lambda}_i|^{1/2}}{|\mathbf{D}|^{1/2}} \exp\left(\frac{1}{2} \left[(\mathbf{u}_i^*)' H_f(\mathbf{u}_i^*) \mathbf{u}_i^* - \mathbf{b}' \mathbf{D}^{-1} \mathbf{b} + \boldsymbol{\mu}_i' \boldsymbol{\Lambda}_i^{-1} \boldsymbol{\mu}_i \right] \right).$$

Therefore, to find the parameters $\mathbf{b}$ and $\mathbf{D}$ that maximize the approximate marginal likelihood (S5.9), we can maximize

$$\log L(\mathbf{b}, \mathbf{D}) = N(2\pi)^{P/2} + \sum_{i=1}^{N} \frac{1}{2} \left(\log |\boldsymbol{\Lambda}_i| - \log |\mathbf{D}| \right) + \sum_{i=1}^{N} \frac{1}{2} \left((\mathbf{u}_i^*)' H_f(\mathbf{u}_i^*) \mathbf{u}_i^* - \mathbf{b}' \mathbf{D}^{-1} \mathbf{b} + \boldsymbol{\mu}_i' \boldsymbol{\Lambda}_i^{-1} \boldsymbol{\mu}_i \right)$$

with respect to $\mathbf{b}$ and $\mathbf{D}$. $\mathbf{D}$ has to be positive definite, but unconstrained optimization can be carried out if we optimize over the Cholesky factor $\mathbf{E}$ for $\mathbf{D}$, i.e. the lower-triangular matrix for which $\mathbf{D} = \mathbf{E}\mathbf{E}'$. Overall, the optimization problem will contain $P + \frac{1}{2}P(P+1)$ variables, which grows quadratically with P (the dimension of $\boldsymbol{\beta}_i$).

S5.3 Performance of the approximations

In our tests, both approximations were able to recover the parameters of known log-normal distributions with excellent accuracy, as long as $\mathbf{C}_i$ (the estimation uncertainty of each $\boldsymbol{\beta}_i$) is not too large relative to $\mathbf{D}$ (the log-covariance of the population). The first approximation is more easily scalable to higher dimensions P. The second one was tested with a maximal value of $P = 6$ (27 unknown variables), where the optimization (carried out by the `fmincon` Matlab function using large upper and lower bounds) takes several seconds to converge.

In the example plots below, the two approximation methods are tested in terms of their capacity to approximate log-normal distributions for the FP maturation example.

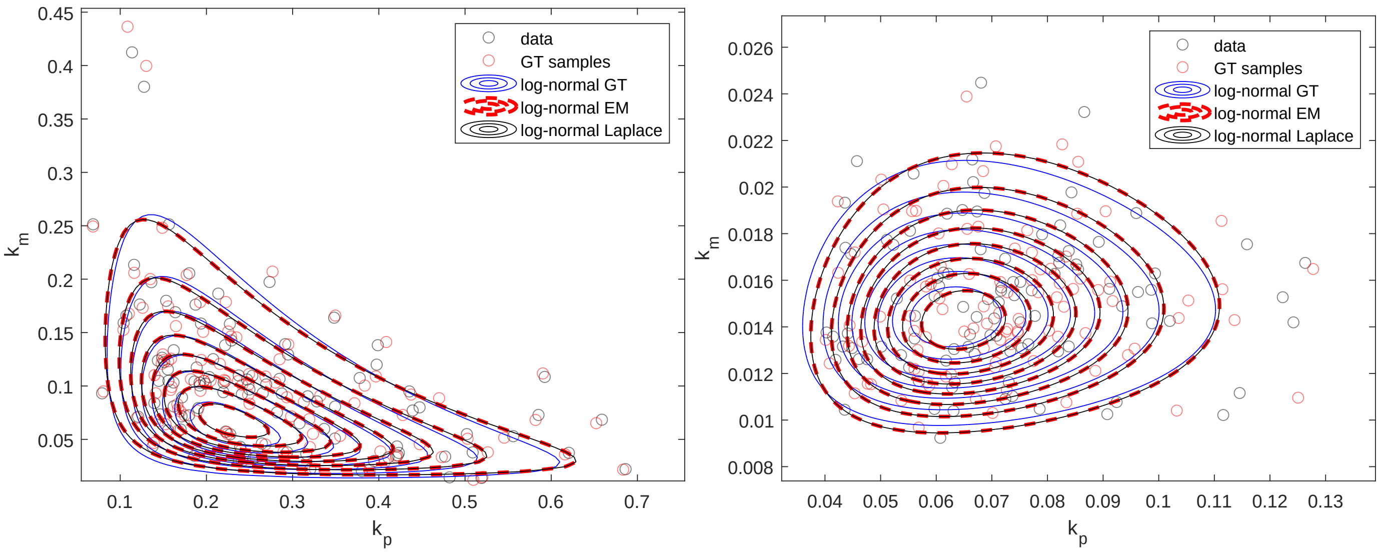

Figure S2: Level sets of log-normal ground-truth distributions for the maturation system and their approximations. Left plot: sfGFP. Right plot: mKate2. For each plot, we used the log-normal distribution parameters inferred from the experimental data to generate a sample of $N = 100$ points (red markers, representing the ground-truth (GT) data). These samples were contaminated with additive, zero-mean, component-wise independent Gaussian noise to mimic the individual parameter estimates from the GMGTS stage (grey markers). The coefficient of variation of the Gaussian noise was obtained by averaging the coefficients of variation of individual estimates from the sfGFP and mKate2 inferece runs. The two approximate schemes described above were used to infer the underlying log-normal distribution, and produced nearly overlapping distributions (red dashed and continuous black lines) which are very close to the groun-truth log-normal distribution (continuous blue line). The same contour levels were used for all three distributions to make them comparable with each other.

S6 B-spline smoothing

In this section, we present the details on how we construct our B-spline basis, estimate spline coefficients, and approximate the error variance of the measurements. As in § S2.3, we drop the subscripts i for readability for the majority of this section, writing $\mathbf{x} := \mathbf{x}_i$ and $\mathbf{y} := \mathbf{y}_i$ (see (S2.6) and (S2.4)) for each statement that applies to every individual independently of the others. Denote the number of observed states by L, so that $L \leqslant K$, and assume without loss of generality that the first L states are observed. For the i-th cell, we estimate each observed component of $x_k(\cdot)$ for $k = 1, \ldots, L$ using a basis expansion of an M-dimensional B-spline basis $\{B_{1,\nu+1}(\cdot), \ldots, B_{M,\nu+1}(\cdot)\}$ of degree $\nu = 3$ (order $\nu + 1 = 4$) with $M - \nu - 1$ internal knots $\kappa_1, \ldots, \kappa_{M-\nu-1} \in (t_1, t_T)$, cf. [Ghosal and van der Vaart, 2017, App. E.2]. Denote

$$\mathbf{B}(\cdot) := \begin{bmatrix} B_{1,\nu+1}(\cdot) & \cdots & B_{M,\nu+1}(\cdot) \end{bmatrix},$$

and let $\boldsymbol{\delta}_k$ be an M-dimensional vector of coefficients for each $k = 1, \ldots, L$. We consistently use $\nu = 4$ (cubic B-splines) throughout our tests, but there is no restriction to do so since any $\nu \geqslant 2$ produces continuous derivatives. We can then approximate

$$x_k(t) \approx \mathbf{B}(t)\boldsymbol{\delta}_k \quad \text{for} \quad t \in [t_1, t_T]$$

for each $k = 1, \ldots, L$. Collecting all basis elements in the block matrix $\mathbf{N}(t) = \mathbf{I}_L \otimes \mathbf{B}(t)$ and the coefficient vectors in $\boldsymbol{\delta} := [\boldsymbol{\delta}_1, \ldots, \boldsymbol{\delta}_L]$, we can approximate the observed states $\mathbf{x}^O(t)$ $(= \mathbf{x}(t)$ when $L = K)$ as

$$\mathbf{x}^O(t) := \begin{bmatrix} x_1(t) \\ \vdots \\ x_L(t) \end{bmatrix} \approx \mathbf{N}(t)\boldsymbol{\delta} \quad \text{for} \quad t \in [t_1, t_T].$$

Plugging in the measurement time points in $\mathbf{x}^O(t)$ and $\mathbf{N}(t)$, we obtain the matrices $\mathbf{X}^O := [\mathbf{x}^O(t_1), \ldots, \mathbf{x}^O(t_T)]$ and $\mathbf{Z} := [\mathbf{N}(t_1), \ldots, \mathbf{N}(t_T)]$, which are related via

$$\mathbf{X}^O \approx \mathbf{Z}\boldsymbol{\delta}.$$

This approximation suggests the following (weighted) linear regression framework for estimating the spline coefficients: let $\mathbf{Y} = [\mathbf{y}(t_1), \dots, \mathbf{y}(t_T)]$ be the combined (TL)-dimensional vector of observed data. Then,

$$\mathbf{Y} = \mathbf{Z}\boldsymbol{\delta} + \varepsilon, \quad \text{where} \quad \varepsilon \sim \mathrm{N}(\mathbf{0}, \boldsymbol{\Sigma}).$$

The diagonal error covariance matrix $\boldsymbol{\Sigma}$ is determined by (S2.5), i.e.

$$\boldsymbol{\Sigma} := \mathrm{diag}(\mathbf{v}(t_1), \dots \mathbf{v}(t_T)), \quad \text{where} \quad \mathbf{v}(t_j) := \mathrm{diag}(\sigma_1^2 + \tau_1^2 \left[x_1^*(t_j; \boldsymbol{\beta})\right]^2, \dots, \sigma_L^2 + \tau_L^2 \left[x_L^*(t_j; \boldsymbol{\beta})\right]^2). \tag{S6.1}$$

Using $\boldsymbol{\Sigma}$ as a weight matrix, we obtain the (weighted sum of squares) cost function

$$L(\boldsymbol{\delta}) := (\mathbf{Y} - \mathbf{Z}\boldsymbol{\delta})' \boldsymbol{\Sigma}^{-1} (\mathbf{Y} - \mathbf{Z}\boldsymbol{\delta}).$$

Since the parameters that $\boldsymbol{\Sigma}$ contains (S6.1) are generally unknown, they have to be estimated in a feasible weighted least squares (WLS) scheme. Given an estimate $\hat{\boldsymbol{\Sigma}}$ of $\boldsymbol{\Sigma}$, the WLS estimate of $\boldsymbol{\delta}$ becomes

$$\hat{\boldsymbol{\delta}} := \arg\min_{\boldsymbol{\delta}} L(\boldsymbol{\delta}) = (\mathbf{Z}' \hat{\boldsymbol{\Sigma}}^{-1} \mathbf{Z})^{-1} \mathbf{Z}' \hat{\boldsymbol{\Sigma}}^{-1} \mathbf{Y}. \tag{S6.2}$$

The log-likelihood of each (σ_k, τ_k) is given by (S2.8) with $\hat{x}_{ik}(t_j)$ replacing $x_{ik}^*(t_j; \boldsymbol{\beta})$ (note that we need to consider the cell index i here). To find the corresponding maximum likelihood estimate $(\hat{\sigma}_k, \hat{\tau}_k)$, we initialize it at the non-negative least squares estimate

$$(\tilde{\sigma}_k, \tilde{\tau}_k) = \arg\min_{\sigma_k, \tau_k \geqslant 0} \sum_{i=1}^{N} \sum_{j=1}^{T} \left[(y_{ik}(t_j) - \hat{x}_{ik}(t_j))^2 - \left(\sigma_k^2 + \tau_k^2 \left[x_{ik}^*(t_j; \boldsymbol{\beta})\right]^2 \right) \right]^2, \tag{S6.3}$$

and subsequently optimize the log-likelihood of (σ_k, τ_k) using an interior-point algorithm, for each $k = 1, \dots, L$. After alternating the estimation of $\boldsymbol{\delta}$ and $\boldsymbol{\Sigma}$ until convergence, we approximate

$$\mathbf{x}^O(t) \approx \mathbf{N}(t) \hat{\boldsymbol{\delta}} := \hat{\mathbf{x}}^O(t) \tag{S6.4}$$

and

$$\dot{\mathbf{x}}^O(t) \approx \dot{\mathbf{N}}(t) \hat{\boldsymbol{\delta}} =: \hat{\dot{\mathbf{x}}}^O(t) = \dot{\hat{\mathbf{x}}}^O(t) \quad \text{for} \quad t \in [t_1, t_T], \tag{S6.5}$$

where $\dot{\mathbf{N}}(\cdot)$ contains the time derivatives of the B-spline basis functions contained in $\mathbf{N}(\cdot)$. Anogously to $\mathbf{Z}$, we define $\dot{\mathbf{Z}} := [\dot{\mathbf{N}}(t_1), \dots, \dot{\mathbf{N}}(t_T)]$.

S7 Full observation: estimation of the residual covariance matrix and the covariances of individual parameter estimates

In order to infer $\boldsymbol{\beta}$ in (S2.12) using FGLS (as in the previous section, the cell index i has been omitted), an estimate of the residual covariance matrix

$$\hat{\mathbf{V}}(\boldsymbol{\beta}) \approx \mathrm{Var}\{\hat{\dot{\mathbf{X}}} - \hat{\mathbf{G}}\boldsymbol{\beta} - \hat{\mathbf{H}} \mid \boldsymbol{\beta}\}.$$

is required. Note the inclusion of the right-hand side term $\hat{\mathbf{G}}\boldsymbol{\beta}$, which is necessary because $\hat{\mathbf{G}}$ depends on the smoothed measurements $\hat{\mathbf{X}}$ and therefore is affected by measurement uncertainty. Upon convergence of the FGLS algorithm, an approximation of $\mathbf{C}$, the covariance matrix of $\hat{\boldsymbol{\beta}}$, is required to infer the population parameters in the second stage of GMGTS (cf. §S2.3). The present section provides derivations for $\hat{\mathbf{V}}(\boldsymbol{\beta})$ and $\mathbf{C}$, which are needed in §S2.3.

Up to this point, state values across measurement time points have been organized in block vectors and block matrices using T blocks of K or L states. However, in this and the following section, we will use block matrices with K or L blocks of T time points. This rearrangement uniformizes the notation for fully and partially observed systems. Concretely, we define

$$\mathbf{X} := \begin{bmatrix} x_1(t_1) \\ \vdots \\ x_1(t_T) \\ \vdots \\ x_K(t_1) \\ \vdots \\ x_K(t_T) \end{bmatrix}, \quad \mathbf{G} := \begin{bmatrix} \mathbf{g}_1(\mathbf{x}(t_1)) \\ \vdots \\ \mathbf{g}_1(\mathbf{x}(t_T)) \\ \vdots \\ \mathbf{g}_K(\mathbf{x}(t_1)) \\ \vdots \\ \mathbf{g}_K(\mathbf{x}(t_T)) \end{bmatrix}, \quad \mathbf{H} := \begin{bmatrix} \mathbf{h}_1(\mathbf{x}(t_1)) \\ \vdots \\ \mathbf{h}_1(\mathbf{x}(t_T)) \\ \vdots \\ \mathbf{h}_K(\mathbf{x}(t_1)) \\ \vdots \\ \mathbf{h}_K(\mathbf{x}(t_T)) \end{bmatrix}, \quad \boldsymbol{\Sigma} := \mathrm{diag}\left(\begin{bmatrix} \sigma_1^2 + \tau_1^2 \left[x_1^*(t_1; \boldsymbol{\beta})\right]^2 \\ \vdots \\ \sigma_1^2 + \tau_1^2 \left[x_1^*(t_T; \boldsymbol{\beta})\right]^2 \\ \vdots \\ \sigma_L^2 + \tau_L^2 \left[x_L^*(t_1; \boldsymbol{\beta})\right]^2 \\ \vdots \\ \sigma_L^2 + \tau_L^2 \left[x_L^*(t_T; \boldsymbol{\beta})\right]^2 \end{bmatrix} \right), \quad \mathbf{Z} := \mathbf{I}_L \otimes \begin{bmatrix} \mathbf{B}(t_1) \\ \vdots \\ \mathbf{B}(t_T) \end{bmatrix}, \tag{S7.1}$$

and consider analogous rearrangements for the estimates and the derivatives of these quantities. Similarly to the above, $\boldsymbol{\delta}$ and $\hat{\boldsymbol{\delta}}$ are organized into L blocks of M basis functions (as in §S6). Note that the GLS estimate expression (S2.15) is invariant with respect to the applied reordering.

Derivation of $\hat{\mathbf{V}}(\boldsymbol{\beta})$: To infer the parameters of the generalized linear model (S2.12), the covariance matrix of $\boldsymbol{\Delta}$ needs to be

estimated alongside the parameter vector β at each iteration of the generalized least squares algorithm. For this calculation, an approximation of $\mathrm{Var}[\hat{\mathbf{X}}, \hat{\dot{\mathbf{X}}}]$ is required. To produce this approximation, we propagate the uncertainty from the measurements to the B-splines and their derivatives through the spline coefficient vector $\hat{\delta}$, making use of the definitions of the spline basis matrices $\mathbf{Z}$, $\dot{\mathbf{Z}}$, and the error covariance matrix estimate $\hat{\boldsymbol{\Sigma}}$ provided in § S6 but reordered as stated above.

Our first goal is to calculate $\mathrm{Var}\,\hat{\delta}$, taking into account that $\hat{\delta}$ is a linear combination of the measurements $\mathbf{Y}$ (see (S6.2)). $\mathrm{Var}\,\hat{\delta}$ depends on the error covariance matrix $\mathrm{Var}\{\mathbf{Y}\} = \boldsymbol{\Sigma}$ defined in (S6.1):

$$\mathrm{Var}\,\hat{\delta} = (\mathbf{Z}'\hat{\boldsymbol{\Sigma}}^{-1}\mathbf{Z})^{-1}\mathbf{Z}'\hat{\boldsymbol{\Sigma}}^{-1}\boldsymbol{\Sigma}\hat{\boldsymbol{\Sigma}}^{-1}\mathbf{Z}(\mathbf{Z}'\hat{\boldsymbol{\Sigma}}^{-1}\mathbf{Z})^{-1}. \tag{S7.2}$$

Replacing $\boldsymbol{\Sigma}$ with the estimate $\hat{\boldsymbol{\Sigma}}$ defined in § S6, we arrive at the empirical covariance $\boldsymbol{\Omega}$ of the spline coefficient vector $\hat{\delta}$:

$$\boldsymbol{\Omega} := (\mathbf{Z}'\hat{\boldsymbol{\Sigma}}^{-1}\mathbf{Z})^{-1}. \tag{S7.3}$$

To estimate $\mathrm{Var}[\hat{\mathbf{X}}, \hat{\dot{\mathbf{X}}}]$, recall that $\hat{\mathbf{X}} = \mathbf{Z}\hat{\delta}$ and $\hat{\dot{\mathbf{X}}} = \dot{\mathbf{Z}}\hat{\delta}$ are, in turn, linear combinations of $\hat{\delta}$. Therefore,

$$\mathrm{Var}\begin{bmatrix}\hat{\mathbf{X}}\\ \hat{\dot{\mathbf{X}}}\end{bmatrix} \approx \begin{bmatrix}\mathbf{Z}\\ \dot{\mathbf{Z}}\end{bmatrix}\boldsymbol{\Omega}\begin{bmatrix}\mathbf{Z}\\ \dot{\mathbf{Z}}\end{bmatrix}'. \tag{S7.4}$$

Using (S7.4), the error between the smoothed and actual states and their derivatives is approximately given by [Zhou et al., 1998]

$$\begin{bmatrix}\hat{\mathbf{X}}\\ \hat{\dot{\mathbf{X}}}\end{bmatrix} - \begin{bmatrix}\mathbf{X}\\ \dot{\mathbf{X}}\end{bmatrix} - \begin{bmatrix}\mathbf{bias}\\ \dot{\mathbf{bias}}\end{bmatrix} \sim \mathrm{N}\left(\mathbf{0}, \begin{bmatrix}\mathbf{Z}\\ \dot{\mathbf{Z}}\end{bmatrix}\boldsymbol{\Omega}\begin{bmatrix}\mathbf{Z}\\ \dot{\mathbf{Z}}\end{bmatrix}'\right). \tag{S7.5}$$

We ignore the bias term in (S7.5) since there is no way to reliably estimate it (because it depends on derivatives of the ground truth ODE solution) and it vanishes as the number of measurement time points increases [Wu et al., 2012].

Finally, to compute $\hat{\mathbf{V}}(\beta)$ at a fixed value of β, we use the delta method [Berger and Casella, 2001, § 5.5] together with (S7.5) to obtain

$$\mathrm{Var}\{\hat{\dot{\mathbf{X}}} - \hat{\mathbf{G}}\beta - \hat{\mathbf{H}} \mid \beta\} \approx \hat{\mathbf{V}}(\beta) := \left[-\frac{\partial}{\partial \mathbf{X}}\left(\mathbf{G}(\hat{\mathbf{X}})\beta + \mathbf{H}(\hat{\mathbf{X}})\right) \quad \mathbf{I}_{TK}\right]\begin{bmatrix}\mathbf{Z}\\ \dot{\mathbf{Z}}\end{bmatrix}\boldsymbol{\Omega}\begin{bmatrix}\mathbf{Z}\\ \dot{\mathbf{Z}}\end{bmatrix}'\left[-\frac{\partial}{\partial \mathbf{X}}\left(\mathbf{G}(\hat{\mathbf{X}})\beta + \mathbf{H}(\hat{\mathbf{X}})\right) \quad \mathbf{I}_{TK}\right]'. \tag{S7.6}$$

From the definitions at the start of this section, one can see that $\hat{\mathbf{V}}(\beta)$ is organized into $K \times K$ square blocks of size $T \times T$.

Derivation of $\mathrm{Var}\,\hat{\beta}$: Upon convergence of the FGLS algorithm presented in § S2.3, we can use the covariance matrix of the splines and their gradients to obtain an estimate of the uncertainty of $\hat{\beta}$. To this end, the delta-method approximation (S2.16) provides better accuracy compared to the inverse Fisher information matrix $(\hat{\mathbf{G}}'\hat{\mathbf{V}}(\hat{\beta})^{-1}\hat{\mathbf{G}})^{-1}$. This is because the splines introduce uncertainty to the design matrix $\hat{\mathbf{G}}$ that is not accounted for in the Fisher information matrix.

Combining (S2.16) and (S7.5) we get

$$\mathbf{C} := \mathrm{Var}\,\hat{\beta} \approx \begin{bmatrix}\frac{\partial}{\partial \mathbf{X}}\hat{\beta}\\ \frac{\partial}{\partial \dot{\mathbf{X}}}\hat{\beta}\end{bmatrix}\begin{bmatrix}\mathbf{Z}\\ \dot{\mathbf{Z}}\end{bmatrix}\boldsymbol{\Omega}\begin{bmatrix}\mathbf{Z}\\ \dot{\mathbf{Z}}\end{bmatrix}'\begin{bmatrix}\frac{\partial}{\partial \mathbf{X}}\hat{\beta}\\ \frac{\partial}{\partial \dot{\mathbf{X}}}\hat{\beta}\end{bmatrix}'. \tag{S7.7}$$

Recall that when the GSS criterion is not maximized at the boundary of the parameter space (cf. Remark S5), $\hat{\beta}$ is given by (S2.15), where $\hat{\mathbf{V}}$ is obtained using (S7.6) by plugging in the value of $\hat{\beta}$ from the previous FGLS iteration. To estimate $\mathrm{Var}\,\hat{\beta}$ from (S7.7), we need to calculate the partial derivatives of $\hat{\beta}$ with respect to the state and gradient estimates. From (S2.15), it is straightforward to see that

$$\frac{\partial}{\partial \dot{\mathbf{X}}}\hat{\beta} = \left(\hat{\mathbf{G}}'\hat{\mathbf{V}}^{-1}\hat{\mathbf{G}}\right)^{-1}\hat{\mathbf{G}}'\hat{\mathbf{V}}^{-1}. \tag{S7.8}$$

To compute $\frac{\partial}{\partial \mathbf{X}}\hat{\beta}$, we apply the product rule to (S2.15), i.e.,

$$\frac{\partial}{\partial \mathbf{X}}\hat{\beta} = \underbrace{\frac{\partial}{\partial \mathbf{X}}\left[\left(\hat{\mathbf{G}}'\hat{\mathbf{V}}^{-1}\hat{\mathbf{G}}\right)^{-1}\right]\hat{\mathbf{G}}'\hat{\mathbf{V}}^{-1}\left(\hat{\dot{\mathbf{X}}} - \hat{\mathbf{H}}\right)}_{\textbf{Term 1}} + \underbrace{\left(\hat{\mathbf{G}}'\hat{\mathbf{V}}^{-1}\hat{\mathbf{G}}\right)^{-1}\frac{\partial}{\partial \mathbf{X}}\left[\hat{\mathbf{G}}'\hat{\mathbf{V}}^{-1}\left(\hat{\dot{\mathbf{X}}} - \hat{\mathbf{H}}\right)\right]}_{\textbf{Term 2}}. \tag{S7.9}$$

[**Calculation of Term 1**] We start from the fact that [Petersen and Pedersen, 2008, § 2.2]

$$\frac{\partial}{\partial \mathbf{X}}\left[\left(\hat{\mathbf{G}}'\hat{\mathbf{V}}^{-1}\hat{\mathbf{G}}\right)^{-1}\right] = -\left(\hat{\mathbf{G}}'\hat{\mathbf{V}}^{-1}\hat{\mathbf{G}}\right)^{-1}\frac{\partial}{\partial \mathbf{X}}[\hat{\mathbf{G}}'\hat{\mathbf{V}}^{-1}\hat{\mathbf{G}}]\left(\hat{\mathbf{G}}'\hat{\mathbf{V}}^{-1}\hat{\mathbf{G}}\right)^{-1}.$$

Recall $\hat{\mathbf{V}}^{-1}$ is organized into $K \times K$ blocks of dimension $T \times T$. Let $\hat{v}^{-1}_{k_1k_2,j_1j_2}$ denote the element corresponding to the j_1, j_2-th time point within the k_1, k_2-th block of $\hat{\mathbf{V}}^{-1}$. Moreover, denote the k-th row of $\mathbf{g}(\cdot)$ by $\mathbf{g}_k(\cdot)$, and the respective partial derivatives of $\mathbf{g}(\cdot)$ and $\mathbf{g}_k(\cdot)$ with respect to x_l by $\mathbf{g}_{x_l}(\cdot)$ and $\mathbf{g}_{k,x_l}(\cdot)$. Then

$$\frac{\partial}{\partial x_k(t_j)}[\hat{\mathbf{G}}'\hat{\mathbf{V}}^{-1}\hat{\mathbf{G}}] = \frac{\partial}{\partial x_k(t_j)}\left[\sum_{k_1=1}^{K}\sum_{j_1=1}^{T}\sum_{k_2=1}^{K}\sum_{j_2=1}^{T}\mathbf{g}'_{k_1}\left(\hat{\mathbf{x}}(t_{j_1})\right)\hat{v}^{-1}_{k_1k_2,j_1j_2}\mathbf{g}_{k_2}\left(\hat{\mathbf{x}}(t_{j_2})\right)\right]$$

$$= \sum_{k_1=1}^{K}\sum_{k_2=1}^{K}\sum_{j_2=1}^{T} \mathbf{g}'_{k_1,x_k}\left(\hat{\mathbf{x}}(t_j)\right)\hat{v}^{-1}_{k_1k_2,jj_2}\mathbf{g}_{k_2}\left(\hat{\mathbf{x}}(t_{j_2})\right) + \mathbf{g}'_{k_2}\left(\hat{\mathbf{x}}(t_{j_2})\right)(\hat{v}^{-1}_{k_1k_2,jj_2})'\mathbf{g}_{k_1,x_k}\left(\hat{\mathbf{x}}(t_j)\right)$$
$$= \mathbf{g}'_{x_k}\left(\hat{\mathbf{x}}(t_j)\right)\left(\hat{\mathbf{V}}^{-1}\right)_j\hat{\mathbf{G}} + \hat{\mathbf{G}}'\left(\hat{\mathbf{V}}^{-1}\right)'_j\mathbf{g}_{x_k}\left(\hat{\mathbf{x}}(t_j)\right),$$

where $\left(\hat{\mathbf{V}}^{-1}\right)_j$ is the submatrix of $\hat{\mathbf{V}}^{-1}$ with only the j-th row of each $T\times T$-dimensional block. Denote

$$\begin{aligned}\mathbf{\Theta} &:= \hat{\mathbf{G}}'\hat{\mathbf{V}}^{-1}\hat{\mathbf{G}},\\ \mathbf{\Xi} &:= \hat{\mathbf{G}}'\hat{\mathbf{V}}^{-1}\left(\hat{\hat{\mathbf{X}}} - \hat{\mathbf{H}}\right),\\ \mathbf{\Phi}_{kj} &:= \mathbf{g}'_{x_k}\left(\hat{\mathbf{x}}(t_j)\right)\left(\hat{\mathbf{V}}^{-1}\right)_j\hat{\mathbf{G}} + \hat{\mathbf{G}}'\left(\hat{\mathbf{V}}^{-1}\right)'_j\mathbf{g}_{x_k}\left(\hat{\mathbf{x}}(t_j)\right),\end{aligned} \tag{S7.10}$$

then

$$\frac{\partial}{\partial x_k(t_j)}\left[\left(\hat{\mathbf{G}}'\hat{\mathbf{V}}^{-1}\hat{\mathbf{G}}\right)^{-1}\right]\hat{\mathbf{G}}'\hat{\mathbf{V}}^{-1}\left(\hat{\hat{\mathbf{X}}} - \hat{\mathbf{H}}\right) = -\mathbf{\Theta}^{-1}\mathbf{\Phi}_{kj}\mathbf{\Theta}^{-1}\mathbf{\Xi}. \tag{S7.11}$$

Horizontally stacking (S7.11) across time points and states, we arrive at

$$\frac{\partial}{\partial \mathbf{X}}\left[\left(\hat{\mathbf{G}}'\hat{\mathbf{V}}^{-1}\hat{\mathbf{G}}\right)^{-1}\right]\hat{\mathbf{G}}'\hat{\mathbf{V}}^{-1}\left(\hat{\hat{\mathbf{X}}} - \hat{\mathbf{H}}\right) = -\mathbf{\Theta}^{-1}\left[\mathbf{\Phi}_{11}\mathbf{\Theta}^{-1}\mathbf{\Xi} \quad \cdots \quad \mathbf{\Phi}_{KT}\mathbf{\Theta}^{-1}\mathbf{\Xi}\right]. \tag{S7.12}$$

[**Calculation of Term 2**] Similarly,

$$\begin{aligned}\frac{\partial}{\partial x_k(t_j)}\left[\hat{\mathbf{G}}'\hat{\mathbf{V}}^{-1}\left(\hat{\hat{\mathbf{X}}} - \hat{\mathbf{H}}\right)\right] &= \frac{\partial}{\partial x_k(t_j)}\left[\sum_{k_1=1}^{K}\sum_{j_1=1}^{T}\sum_{k_2=1}^{K}\sum_{j_2=1}^{T}\mathbf{g}'_{k_1}\left(\hat{\mathbf{x}}(t_{j_1})\right)\hat{v}^{-1}_{k_1k_2,j_1j_2}\left(\hat{\hat{x}}_{k_2}(t_{j_2}) - h_{k_2}(\hat{\mathbf{x}}(t_{j_2}))\right)\right]\\ &= \sum_{k_1=1}^{K}\sum_{k_2=1}^{K}\sum_{j_2=1}^{T}\Bigg(\left(\mathbf{g}'_{k_1,x_k}\left(\hat{\mathbf{x}}(t_j)\right)\hat{v}^{-1}_{k_1k_2,jj_2}\left(\hat{\hat{x}}_{k_2}(t_{j_2}) - h_{k_2}(\hat{\mathbf{x}}(t_{j_2}))\right)\right)\\ &\qquad - \mathbf{g}'_{k_2}\left(\hat{\mathbf{x}}(t_{j_2})\right)(\hat{v}^{-1}_{k_1k_2,jj_2})'h_{k_2,x_k}(\hat{\mathbf{x}}(t_j))\Bigg)\\ &= \mathbf{g}'_{x_k}\left(\hat{\mathbf{x}}(t_j)\right)\left(\hat{\mathbf{V}}^{-1}\right)_j\left(\hat{\hat{\mathbf{X}}} - \hat{\mathbf{H}}\right) - \hat{\mathbf{G}}'\left(\hat{\mathbf{V}}^{-1}\right)'_j\mathbf{h}_{x_k}(\hat{\mathbf{x}}(t_j))\\ &=: \mathbf{\Psi}_{kj}\end{aligned}$$

This means

$$\left(\hat{\mathbf{G}}'\hat{\mathbf{V}}^{-1}\hat{\mathbf{G}}\right)^{-1}\frac{\partial}{\partial \mathbf{X}}\left[\hat{\mathbf{G}}'\hat{\mathbf{V}}^{-1}\left(\hat{\hat{\mathbf{X}}} - \hat{\mathbf{H}}\right)\right] = \mathbf{\Theta}^{-1}\left[\mathbf{\Psi}_{11} \quad \cdots \quad \mathbf{\Psi}_{KT}\right]. \tag{S7.13}$$

Combining (S7.12) and (S7.13), we obtain

$$\tfrac{\partial}{\partial \mathbf{X}}\hat{\beta} = \mathbf{\Theta}^{-1}\left[\mathbf{\Psi}_{11} - \mathbf{\Phi}_{11}\mathbf{\Theta}^{-1}\mathbf{\Xi} \quad \cdots \quad \mathbf{\Psi}_{KT} - \mathbf{\Phi}_{KT}\mathbf{\Theta}^{-1}\mathbf{\Xi}\right], \tag{S7.14}$$

and thus an explicit formula to approximate (S7.7).

S8 Partial observation: estimation of the residual covariance matrix and the covariance of individual parameter estimates

The goal of this section is to reconcile the residual covariance expression (S7.6) with the treatment of partially observed systems. To this end, uncertainty needs to be propagated in accordance with the iterative scheme presented in § S2.4. The flow of information, and thus of uncertainty, in the algorithm is depicted in Figure S3. As in § S7, block vectors and block matrices are organized as K or L blocks of T time points as shown in (S7.1). Without loss of generality, we assume the last $K - L$ states are hidden, so $\mathbf{X}^O$ simply consists of the first L blocks of $\mathbf{X}$. Analogously, the vector $\mathbf{X}^H$ containing the time point blocks for the hidden states consists of the last $K - L$ blocks of $\mathbf{X}$. The same holds for estimates, gradients, and estimates of gradients of $\mathbf{X}^O$ and $\mathbf{X}^H$.

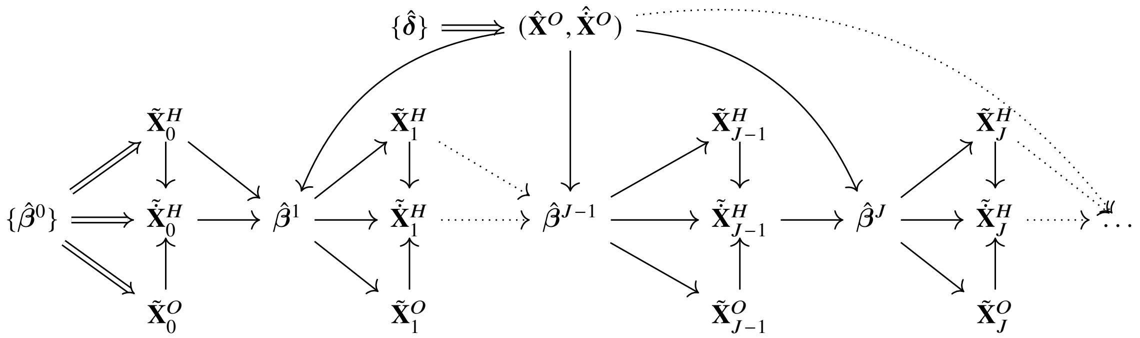

Figure S3: Dependence hierarchy in the iterative optimization scheme for partially observed systems. Uncertainty enters the scheme through the initial parameter estimate $\hat{\boldsymbol{\beta}}^0$ and the spline coefficient estimates $\hat{\boldsymbol{\delta}}$. These estimates determine the initial hidden state estimates ($\hat{\mathbf{X}}_0^H$ and $\hat{\dot{\mathbf{X}}}_0^H$) and the smoothed measurements ($\hat{\mathbf{X}}^O$ and $\hat{\dot{\mathbf{X}}}^O$), respectively, as well as their associated uncertainties. As soon as we obtain $\hat{\mathbf{X}}_0^H$ and $\hat{\dot{\mathbf{X}}}_0^H$, we use these values together with the smoothed measurements ($\hat{\mathbf{X}}^O$ and $\hat{\dot{\mathbf{X}}}^O$) to obtain a new parameter estimate $\hat{\boldsymbol{\beta}}^J$ ($J \geq 1$) via gradient matching. We also calculate the uncertainty of $\hat{\boldsymbol{\beta}}^J$ from the covariance of hidden and observed states and their derivatives at step $J-1$. The new $\hat{\boldsymbol{\beta}}^J$ estimate gives rise to new hidden state estimates ($\hat{\mathbf{X}}_J^H$ and $\hat{\dot{\mathbf{X}}}_J^H$) with their corresponding uncertainty, and the cycle repeats.

Our iterative estimation scheme starts with smoothed observed states $\hat{\mathbf{X}}^O$ (with gradients $\hat{\dot{\mathbf{X}}}^O$) from the estimated spline coefficients $\hat{\boldsymbol{\delta}}$ and an initial estimate $\hat{\boldsymbol{\beta}}^0$ of the cell-specific parameters. The spline coefficients and initial cell-specific parameter estimates have covariance matrices $\operatorname{Var}\hat{\boldsymbol{\delta}}$ (from (S7.3)) and $\operatorname{Var}\hat{\boldsymbol{\beta}}^0$ (discussed below). The smoothed observed states and their derivatives have their own covariance matrix in terms of $\operatorname{Var}\hat{\boldsymbol{\delta}}$,

$$\operatorname{Var}\begin{bmatrix}\hat{\mathbf{X}}^O \\ \hat{\dot{\mathbf{X}}}^O\end{bmatrix} = \begin{bmatrix}\mathbf{Z} \\ \dot{\mathbf{Z}}\end{bmatrix} \operatorname{Var}\hat{\boldsymbol{\delta}} \begin{bmatrix}\mathbf{Z} \\ \dot{\mathbf{Z}}\end{bmatrix}', \tag{S8.1}$$

that remains fixed throughout iterations of $\hat{\boldsymbol{\beta}}$, cf. (S7.5) and (S7.4).

Following the definition of the initial cell-specific parameter estimates $\hat{\boldsymbol{\beta}}^0$, we integrate the system (S2.6) to obtain the vectors of observed and hidden states, $\tilde{\mathbf{X}}_0^O$ and $\tilde{\mathbf{X}}_0^H$. With respect to notation, from this point on, we use a hat over a symbol to indicate estimated quantities, while a tilde exclusively corresponds to state predictions produced by numerical integration of the system (S2.6) using the current parameter estimate. Since the only way to obtain estimates of the hidden states and their derivatives is via numerical integration, we use the tilde and hat notations interchangeably for those states, i.e.,

$$\hat{\mathbf{X}}_0^H = \tilde{\mathbf{X}}_0^H \quad \text{and} \quad \hat{\dot{\mathbf{X}}}_0^H = \tilde{\dot{\mathbf{X}}}_0^H.$$

For the observed states, however, spline smoothing estimates $\hat{\mathbf{X}}^O$ and $\hat{\dot{\mathbf{X}}}^O$ are available, so in general,

$$\hat{\mathbf{X}}^O \neq \tilde{\mathbf{X}}_0^O \quad \text{and} \quad \hat{\dot{\mathbf{X}}}^O \neq \tilde{\dot{\mathbf{X}}}_0^O.$$

We can combine the estimated hidden and observed states and their gradients into

$$\hat{\mathbf{X}}_0 := \begin{bmatrix}\hat{\mathbf{X}}^O \\ \hat{\mathbf{X}}_0^H\end{bmatrix} \quad \text{and} \quad \hat{\dot{\mathbf{X}}}_0 := \begin{bmatrix}\hat{\dot{\mathbf{X}}}^O \\ \hat{\dot{\mathbf{X}}}_0^H\end{bmatrix}. \tag{S8.2}$$

As discussed above, the covariance matrix describing the uncertainty in $\hat{\boldsymbol{\beta}}^0$ needs to be initialized at the start of the iterations, and before any state, gradient or residual covariance is calculated. To prevent subsequent iterations from staying to close to $\hat{\boldsymbol{\beta}}^0$, we prescribe a large uncertainty with a coefficient of variation equal to 0.5 for each component of $\hat{\boldsymbol{\beta}}^0$ (cf. §S11.3).

To compute the equivalent of (S7.6) for the partial observation case, we first need an approximation of $\operatorname{Var}[\hat{\dot{\mathbf{X}}}_0, \hat{\mathbf{X}}_0]$. Note that the hidden part of $\hat{\dot{\mathbf{X}}}_0$ depends on $\tilde{\mathbf{X}}_0$ and $\hat{\boldsymbol{\beta}}^0$. Let $\mathbf{F}(\boldsymbol{\beta})$ denote the vector of solution values of (S2.6) given the cell-specific parameter vector $\boldsymbol{\beta}$, that is,

$$\mathbf{F}(\boldsymbol{\beta}) = [F_1(t_1;\boldsymbol{\beta}), \ldots, F_1(t_T;\boldsymbol{\beta}), \ \ldots \ , F_K(t_1;\boldsymbol{\beta}), \ldots, F_K(t_T;\boldsymbol{\beta})],$$

where $F_k(t_j;\boldsymbol{\theta})$ denotes the k-th component of the ODE solution at time t_j. Denote the respective observed and hidden parts by $\mathbf{F}^O(\boldsymbol{\beta})$ (first L blocks of $\mathbf{F}(\boldsymbol{\beta})$) and $\mathbf{F}^H(\boldsymbol{\beta})$ (last $K-L$ blocks of $\mathbf{F}(\boldsymbol{\beta})$). Finally, let $\mathbf{F}_{\boldsymbol{\beta}}(\boldsymbol{\beta})$, $\mathbf{F}_{\boldsymbol{\beta}}^O(\boldsymbol{\beta})$, and $\mathbf{F}_{\boldsymbol{\beta}}^H(\boldsymbol{\beta})$ be the corresponding partial derivatives with respect to $\boldsymbol{\beta}$, which may be obtained by numerically solving the sensitivity equations of (S2.6) or through finite difference approximations. Using the delta method, the full covariance matrix of $\hat{\boldsymbol{\beta}}^0$, $\tilde{\mathbf{X}}_0^O$, and $\tilde{\mathbf{X}}_0^H$ is given by

$$\operatorname{Var}\begin{bmatrix}\hat{\boldsymbol{\beta}}^0 \\ \tilde{\mathbf{X}}_0\end{bmatrix} = \operatorname{Var}\begin{bmatrix}\hat{\boldsymbol{\beta}}^0 \\ \tilde{\mathbf{X}}_0^O \\ \tilde{\mathbf{X}}_0^H\end{bmatrix} \approx \begin{bmatrix}\mathbf{I}_P \\ \mathbf{F}_{\boldsymbol{\beta}}^O(\hat{\boldsymbol{\beta}}^0) \\ \mathbf{F}_{\boldsymbol{\beta}}^H(\hat{\boldsymbol{\beta}}^0)\end{bmatrix} \operatorname{Var}\hat{\boldsymbol{\beta}}^0 \begin{bmatrix}\mathbf{I}_P \\ \mathbf{F}_{\boldsymbol{\beta}}^O(\hat{\boldsymbol{\beta}}^0) \\ \mathbf{F}_{\boldsymbol{\beta}}^H(\hat{\boldsymbol{\beta}}^0)\end{bmatrix}'.$$

We can now compute $\operatorname{Var}[\hat{\mathbf{X}}_0^H, \hat{\dot{\mathbf{X}}}_0^H] = \operatorname{Var}[\tilde{\mathbf{X}}_0^H, \tilde{\dot{\mathbf{X}}}_0^H]$ from the right-hand side of (S2.6) and the preceding covariance matrix as

$$\operatorname{Var}\begin{bmatrix}\hat{\mathbf{X}}_0^H \\ \hat{\dot{\mathbf{X}}}_0^H\end{bmatrix} \approx \begin{bmatrix}\mathbf{0}_{(K-L)T\times P} & \begin{bmatrix}\mathbf{0}_{(K-L)T\times LT} & \mathbf{I}_{(K-L)T\times(K-L)T}\end{bmatrix} \\ \mathbf{G}^H(\tilde{\mathbf{X}}_0) & \frac{\partial}{\partial \mathbf{X}}[\mathbf{G}^H(\tilde{\mathbf{X}}_0)\hat{\boldsymbol{\beta}}^0 + \mathbf{H}^H(\tilde{\mathbf{X}}_0)]\end{bmatrix} \operatorname{Var}\begin{bmatrix}\hat{\boldsymbol{\beta}}^0 \\ \tilde{\mathbf{X}}_0\end{bmatrix} \begin{bmatrix}\mathbf{0}_{(K-L)T\times P} & \begin{bmatrix}\mathbf{0}_{(K-L)T\times LT} & \mathbf{I}_{(K-L)T\times(K-L)T}\end{bmatrix} \\ \mathbf{G}^H(\tilde{\mathbf{X}}_0) & \frac{\partial}{\partial \mathbf{X}}[\mathbf{G}^H(\tilde{\mathbf{X}}_0)\hat{\boldsymbol{\beta}}^0 + \mathbf{H}^H(\tilde{\mathbf{X}}_0)]\end{bmatrix}'. \tag{S8.3}$$

Now that we have derived approximate covariances for the hidden states and their gradients, we can obtain a covariance matrix estimate for the full set of states and gradients. Since $\hat{\delta}$ and $\hat{\beta}^0$ were estimated independently,

$$\operatorname{Var}\begin{bmatrix}\hat{\mathbf{X}}^O\\ \hat{\dot{\mathbf{X}}}^O\\ \hat{\mathbf{X}}^H_0\\ \hat{\dot{\mathbf{X}}}^H_0\end{bmatrix} = \begin{bmatrix}\operatorname{Var}\begin{bmatrix}\hat{\mathbf{X}}^O_0\\ \hat{\dot{\mathbf{X}}}^O_0\end{bmatrix} & \mathbf{0}_{2LT\times 2(K-L)T}\\ \mathbf{0}_{2(K-L)T\times 2LT} & \operatorname{Var}\begin{bmatrix}\hat{\mathbf{X}}^H\\ \hat{\dot{\mathbf{X}}}^H\end{bmatrix}\end{bmatrix},$$

Reorganizing row and column blocks according to (S8.2), we obtain an approximation for $\operatorname{Var}[\hat{\mathbf{X}}_0, \hat{\dot{\mathbf{X}}}_0]$. This covariance will be needed in the calculation of $\hat{\mathbf{V}}_0 \approx \operatorname{Var}\{\hat{\dot{\mathbf{X}}}_0 - \mathbf{G}(\hat{\mathbf{X}}_0)\hat{\beta}^0 - \mathbf{H}(\hat{\mathbf{X}}_0) \mid \hat{\beta}^0\}$ below (i.e., (S8.4) with $J = 0$).

From this point onward, we consider expressions at a general iteration $J \geqslant 0$. Given the approximation of the covariance matrix $\operatorname{Var}[\hat{\mathbf{X}}_J, \hat{\dot{\mathbf{X}}}_J]$, we use another delta method approximation to obtain $\hat{\mathbf{V}}_J \approx \operatorname{Var}\{\hat{\dot{\mathbf{X}}}_J - \mathbf{G}(\hat{\mathbf{X}}_J)\hat{\beta}^J - \mathbf{H}(\hat{\mathbf{X}}_J) \mid \hat{\beta}^J\}$ as

$$\hat{\mathbf{V}}_J := \hat{\mathbf{V}}(\hat{\beta}^J) := \left[-\tfrac{\partial}{\partial \mathbf{X}_J}[\mathbf{G}(\hat{\mathbf{X}}_J)\hat{\beta}^J + \mathbf{H}(\hat{\mathbf{X}}_J)] \quad \mathbf{I}_{KT}\right] \operatorname{Var}\begin{bmatrix}\hat{\mathbf{X}}_J\\ \hat{\dot{\mathbf{X}}}_J\end{bmatrix} \left[-\tfrac{\partial}{\partial \mathbf{X}_J}[\mathbf{G}(\hat{\mathbf{X}}_J)\hat{\beta}^J + \mathbf{H}(\hat{\mathbf{X}}_J)] \quad \mathbf{I}_{KT}\right]' \quad (J \geqslant 0). \tag{S8.4}$$

The above expression requires $\operatorname{Var}[\hat{\mathbf{X}}_J, \hat{\dot{\mathbf{X}}}_J]$. However, the approximation of this covariance is more involved for $J \geqslant 1$ compared to the $J = 0$ presented above. This happens because $\hat{\mathbf{X}}_{J-1}$ and $\hat{\dot{\mathbf{X}}}_{J-1}$ are used to obtain $\hat{\beta}^J$ in a gradient matching step, as presented in § S2.3. In turn, this fact implies that the uncertainty in these vectors will also affect $\hat{\beta}^J$. Specifically, $\hat{\beta}^J$ is given by

$$\hat{\beta}^J := \left(\mathbf{G}(\hat{\mathbf{X}}_{J-1})'\hat{\mathbf{V}}_{J-1}^{-1}\mathbf{G}(\hat{\mathbf{X}}_{J-1})\right)^{-1} \mathbf{G}(\hat{\mathbf{X}}_{J-1})'\hat{\mathbf{V}}_{J-1}^{-1}\left(\hat{\dot{\mathbf{X}}}_{J-1} - \mathbf{H}(\hat{\mathbf{X}}_{J-1})\right). \tag{S8.5}$$

Analogously to (S2.15), this relation holds when the estimate $\hat{\beta}^J$ is not found on the boundary of the parameter space (see Remark S5).

The updated parameter estimate $\hat{\beta}^J$ is subsequently used to produce new hidden state and gradient predictions. As illustrated in Figure S3, this fact implies that uncertainty propagates from $\hat{\mathbf{X}}_{J-1}, \hat{\dot{\mathbf{X}}}_{J-1}$ to $\hat{\mathbf{X}}_J, \hat{\dot{\mathbf{X}}}_J$ through $\hat{\beta}^J$. Therefore, we may consider $\hat{\mathbf{X}}^H_J$ and $\hat{\dot{\mathbf{X}}}^H_J$ as functions of $\hat{\mathbf{X}}_{J-1} = (\hat{\mathbf{X}}^O, \hat{\mathbf{X}}^H_{J-1})$, $\hat{\dot{\mathbf{X}}}_{J-1} = (\hat{\dot{\mathbf{X}}}^O, \hat{\dot{\mathbf{X}}}^H_{J-1})$ and $\tilde{\mathbf{X}}^O_{J-1}$. Then, the relevant sensitivities for $\mathbf{U} \in \{\hat{\mathbf{X}}_{J-1}, \hat{\dot{\mathbf{X}}}_{J-1}\}$ follow from the chain rule as

$$\tfrac{\partial}{\partial \mathbf{U}}\hat{\mathbf{X}}^H_J = \mathbf{F}^H_{\beta}(\hat{\beta}^J)\tfrac{\partial}{\partial \mathbf{U}}\hat{\beta}^J \quad \text{and} \quad \tfrac{\partial}{\partial \mathbf{U}}\hat{\dot{\mathbf{X}}}^H_J = \tfrac{\partial}{\partial \beta^J}[\mathbf{G}^H(\tilde{\mathbf{X}}_J)\hat{\beta}^J + \mathbf{H}^H(\tilde{\mathbf{X}}_J)]\tfrac{\partial}{\partial \mathbf{U}}\hat{\beta}^J, \tag{S8.6}$$

where $\mathbf{G}^H(\tilde{\mathbf{X}}_J)$ and $\mathbf{H}^H(\tilde{\mathbf{X}}_J)$ consist of the last $K - L$ blocks of $\mathbf{G}(\tilde{\mathbf{X}}_J)$ and $\mathbf{H}(\tilde{\mathbf{X}}_J)$. The partial derivatives of $\hat{\beta}^J$ with respect to the gradient and state estimates derive from (S7.8) and (S7.14), respectively. Putting everything together, we find

$$\operatorname{Var}\begin{bmatrix}\hat{\mathbf{X}}_J\\ \hat{\dot{\mathbf{X}}}_J\end{bmatrix} = \operatorname{Var}\begin{bmatrix}\hat{\mathbf{X}}^O\\ \hat{\mathbf{X}}^H_J\\ \hat{\dot{\mathbf{X}}}^O\\ \hat{\dot{\mathbf{X}}}^H_J\end{bmatrix} = \begin{bmatrix}[\mathbf{I}_{LT} \quad \mathbf{0}_{LT\times(K-L)T}] & \mathbf{0}_{KT}\\ \partial\hat{\mathbf{X}}^H_J/\partial\hat{\mathbf{X}}_{J-1} & \partial\hat{\mathbf{X}}^H_J/\partial\hat{\dot{\mathbf{X}}}_{J-1}\\ \mathbf{0}_{KT} & [\mathbf{I}_{LT} \quad \mathbf{0}_{LT\times(K-L)T}]\\ \partial\hat{\dot{\mathbf{X}}}^H_J/\partial\hat{\mathbf{X}}_{J-1} & \partial\hat{\dot{\mathbf{X}}}^H_J/\partial\hat{\dot{\mathbf{X}}}_{J-1}\end{bmatrix} \operatorname{Var}\begin{bmatrix}\hat{\mathbf{X}}_{J-1}\\ \hat{\dot{\mathbf{X}}}_{J-1}\end{bmatrix} \begin{bmatrix}[\mathbf{I}_{LT} \quad \mathbf{0}_{LT\times(K-L)T}] & \mathbf{0}_{KT}\\ \partial\hat{\mathbf{X}}^H_J/\partial\hat{\mathbf{X}}_{J-1} & \partial\hat{\mathbf{X}}^H_J/\partial\hat{\dot{\mathbf{X}}}_{J-1}\\ \mathbf{0}_{KT} & [\mathbf{I}_{LT} \quad \mathbf{0}_{LT\times(K-L)T}]\\ \partial\hat{\dot{\mathbf{X}}}^H_J/\partial\hat{\mathbf{X}}_{J-1} & \partial\hat{\dot{\mathbf{X}}}^H_J/\partial\hat{\dot{\mathbf{X}}}_{J-1}\end{bmatrix}'. \tag{S8.7}$$

As with full state information, upon convergence of the iterative algorithm presented in § S2.4, we can compute the variance of $\hat{\beta}^J$ using

$$\mathbf{C} := \operatorname{Var}\hat{\beta}^J \approx \begin{bmatrix}\frac{\partial}{\partial \mathbf{X}}\hat{\beta}^J\\ \frac{\partial}{\partial \dot{\mathbf{X}}}\hat{\beta}^J\end{bmatrix} \operatorname{Var}\begin{bmatrix}\hat{\mathbf{X}}_{J-1}\\ \hat{\dot{\mathbf{X}}}_{J-1}\end{bmatrix} \begin{bmatrix}\frac{\partial}{\partial \mathbf{X}}\hat{\beta}^J\\ \frac{\partial}{\partial \dot{\mathbf{X}}}\hat{\beta}^J\end{bmatrix}', \tag{S8.8}$$

where $\operatorname{Var}[\hat{\mathbf{X}}_{J-1}, \hat{\dot{\mathbf{X}}}_{J-1}]$ has already been approximated to be able to compute $\hat{\beta}^J$. As mentioned above, the partial derivatives $\frac{\partial}{\partial \mathbf{X}}\hat{\beta}^J$ and $\frac{\partial}{\partial \dot{\mathbf{X}}}\hat{\beta}^J$ are analogous to (S7.8) and (S7.14), respectively.

S9 Including prior information and modifications for maximum a posteriori estimates

In some situations, gradient matching may produce too many parameter estimates at the boundary of the allowable parameter space. When this happens, the uncertainty computations in §S7 and §S8 become inaccurate. This inaccuracy may in turn produce inaccurate estimates of population parameters in the second stage of GMGTS. In such cases, besides redefining the parameter space boundaries, we can incorporate information on likely parameter values by including a prior distribution in the inference of individual parameters.

Let us assume a common prior $\mathrm{N}(\beta_*, \mathbf{\Pi}_*)$ for $\beta_1, \ldots, \beta_N$. Then, the GSS criterion (S2.14) becomes

$$\mathrm{GSS}^*(\beta) = \left(\hat{\dot{\mathbf{X}}} - \hat{\mathbf{G}}\beta - \hat{\mathbf{H}}\right)' \hat{\mathbf{V}}^{-1}\left(\hat{\dot{\mathbf{X}}} - \hat{\mathbf{G}}\beta - \hat{\mathbf{H}}\right) + (\beta - \beta_*)\mathbf{\Pi}_*^{-1}(\beta - \beta_*). \tag{S9.1}$$

Whereas (S2.14) is minimal at the maximum likelihood estimate, the estimate that minimizes $\mathrm{GSS}^*(\beta)$ is the *maximum a posteriori* (MAP) estimate. It is straightforward to verify that the MAP estimate for each β becomes

$$\hat{\beta} := \left(\hat{\mathbf{G}}'\hat{\mathbf{V}}^{-1}\hat{\mathbf{G}} + \mathbf{\Pi}_*^{-1}\right)^{-1}\left(\hat{\mathbf{G}}'\hat{\mathbf{V}}^{-1}\left(\hat{\dot{\mathbf{X}}} - \hat{\mathbf{H}}\right) + \mathbf{\Pi}_*^{-1}\beta_*\right).$$

Since $\boldsymbol{\beta}_*$ and $\boldsymbol{\Pi}_*$ do not depend on any state or gradient estimates, very few adjustments are needed to the uncertainty computations in § S7 and § S8. The only quantity that is directly affected is the parameter estimate $\hat{\boldsymbol{\beta}}$. It immediately follows that

$$\frac{\partial}{\partial \dot{\mathbf{X}}}\hat{\boldsymbol{\beta}} = \left(\hat{\mathbf{G}}'\hat{\mathbf{V}}^{-1}\hat{\mathbf{G}} + \boldsymbol{\Pi}_*^{-1}\right)^{-1}\hat{\mathbf{G}}'\hat{\mathbf{V}}^{-1}. \tag{S9.2}$$

In the computation of $\frac{\partial}{\partial \mathbf{X}}\hat{\boldsymbol{\beta}}$, only undifferentiated factors change. Specifically, $\boldsymbol{\Theta}$ and $\boldsymbol{\Xi}$ in (S7.10) need to be modified, i.e.,

$$\begin{aligned}\boldsymbol{\Theta} &:= \hat{\mathbf{G}}'\hat{\mathbf{V}}^{-1}\hat{\mathbf{G}} + \boldsymbol{\Pi}_*^{-1},\\ \boldsymbol{\Xi} &:= \hat{\mathbf{G}}'\hat{\mathbf{V}}^{-1}\left(\hat{\dot{\mathbf{X}}} - \hat{\mathbf{H}}\right) + \boldsymbol{\Pi}_*^{-1}\boldsymbol{\beta}_*.\end{aligned} \tag{S9.3}$$

S10 Convergence of GMGTS with partial state information

Starting from the smooth estimates of the measurements, the iterative scheme of GMGTS with partial state information (§ S2.4) can be seen as a fixed point iteration. To find the function that is being iterated, we return to the steps outlined in § S2.4. Given the current estimate of individual parameters at the J-th step, $\hat{\boldsymbol{\beta}}^J$, our scheme simulates the ODE system (S2.6) to obtain predictions of the hidden states given $\hat{\boldsymbol{\beta}}^J$ (S2.17). Together with the observed (smoothed) state estimates, these predictions are used to construct the matrix $\hat{\mathbf{X}}(\hat{\boldsymbol{\beta}}^J))$, the matrix containing the hidden state predictions and observed (smoothed) state estimates at the measurement time points. They are also used to obtain $\hat{\dot{\mathbf{X}}}(\hat{\boldsymbol{\beta}}^J)$, the matrix containing the hidden and observed (smoothed) gradient estimates, while the uncertainty of $\hat{\boldsymbol{\beta}}^J$ and the uncertainty of the smoothed state estimates are used to compute the covariance matrix $\hat{\mathbf{V}}(\hat{\boldsymbol{\beta}}^J)$ (S8.4) which is used at the gradient matching step (S8.5).

Putting everything together, the individual parameter vector at step $J+1$ is given by the map

$$\hat{\boldsymbol{\beta}}^{J+1} \leftarrow \mathbf{r}(\hat{\boldsymbol{\beta}}^J) := \left(\hat{\mathbf{G}}'(\hat{\mathbf{X}}(\hat{\boldsymbol{\beta}}^J))\hat{\mathbf{V}}^{-1}(\hat{\boldsymbol{\beta}}^J)\hat{\mathbf{G}}(\hat{\mathbf{X}}(\hat{\boldsymbol{\beta}}^J))\right)^{-1}\hat{\mathbf{G}}'(\hat{\mathbf{X}}(\hat{\boldsymbol{\beta}}^J))\hat{\mathbf{V}}^{-1}(\hat{\boldsymbol{\beta}}^J)\left(\hat{\dot{\mathbf{X}}}(\hat{\boldsymbol{\beta}}^J) - \hat{\mathbf{H}}(\hat{\mathbf{X}}(\hat{\boldsymbol{\beta}}^J))\right). \tag{S10.1}$$

Following the scheme presented in § S2.4, this map is applied iteratively with the aim of letting $\hat{\boldsymbol{\beta}}^J$ converge to a point $\boldsymbol{\beta}^*$ (more practically, the iterations stop when $\hat{\boldsymbol{\beta}}^J$ does not change appreciably from one iteration to the next). Proving that the map $\mathbf{r}(\cdot)$ has a fixed point is challenging. However, once we verify numerically that the map has a fixed point $\boldsymbol{\beta}^*$, we can examine the stability properties of that point. The local stability of $\mathbf{r}(\cdot)$ in terms of the ℓ_2 norm can be assessed by the spectral norm of the Jacobian matrix $\partial\mathbf{r}(\hat{\boldsymbol{\beta}}^J)/\partial\hat{\boldsymbol{\beta}}^J$ (i.e. the norm of $\partial\mathbf{r}(\hat{\boldsymbol{\beta}}^J)/\partial\hat{\boldsymbol{\beta}}^J$ induced by the ℓ_2 norm [Horn and Johnson, 2012]) evaluated at $\boldsymbol{\beta}^*$: if the spectral norm of $\partial\mathbf{r}(\hat{\boldsymbol{\beta}}^J)/\partial\hat{\boldsymbol{\beta}}^J$ is smaller than 1, then there exists a closed neighborhood $O_{\boldsymbol{\beta}^*}$ of $\boldsymbol{\beta}^*$ on which $\mathbf{r}(\cdot)$ is a contraction and the fixed point iteration converges to $\boldsymbol{\beta}^*$ for every starting point in $O_{\boldsymbol{\beta}^*}$ [Sastry, 2013].

Analytical differentiation of $\mathbf{r}(\cdot)$ is very challenging, as it is a composition of matrix functions of ODE solutions. However, the numerical approximation of the Jacobian at $\boldsymbol{\beta}^*$ via finite differences can be used to approximate the spectral norm. Another interesting feature of the map $\mathbf{r}(\cdot)$ from a practical point of view is the basin of attraction of the fixed point $\boldsymbol{\beta}^*$. In a low-dimensional parameter space, the shape of this region can be approximated by testing the convergence of a large number of initial points surrounding $\boldsymbol{\beta}^*$.

A final point to note is that we should more accurately write $\mathbf{r}_i(\cdot)$ instead of $\mathbf{r}(\cdot)$ (following the indexing notation of (S2.1)), since the iterated map is unique for every measured cell. This happens because $\hat{\mathbf{X}}(\hat{\boldsymbol{\beta}}^J))$ contains smoothed estimates of measurements, which differ between cells. Since we start our GMGTS iterations from a common parameter estimate for all cells, a requirement for the convergence of the iterations of $\mathbf{r}_i(\cdot)$, $i = 1, \dots, N$, is that the common initial estimate lies in the basins of attraction of $\mathbf{r}_i(\cdot)$, $i = 1, \dots, N$.

Below, we present a few examples of the aforementioned analyses for a few of the systems considered in the main text. To facilitate visualization in two dimensions, we limited these tests to systems with two parameters. The first system is the generalized Lotka-Volterra system (6) with $P = 2$ unknown parameters, inferred using *in silico* data. Panel A of Figure S4 shows all first-stage estimates with the points of the first five estimates highlighted at the top-left plot. In the remaining plots, the initial estimates that converged to the within an error margin of 5% of the corresponding fixed points are marked blue. For this system, all the initial estimates that we tested converged across all cells. The Jacobian norm was also smaller than 0.1 for all cells, indicating contractivity of the iterated map (S10.1) near the corresponding fixed points.

The second system is the one-step FP maturation model, again inferred using *in silico* data. Following the plotting conventions of Panel A, Panel B shows that there exists a large set of initial points that converge within 5% of the fixed point of each cell, though not all initial points have this property. Importantly, the common initial point $\hat{\boldsymbol{\beta}}^0$ of all GMGTS iterations (cf. §S2.4, denoted by a yellow triangle) lies within the estimated basin of attraction of each cell. Although parameter estimates for each cell quickly converge to the close vicinity of their corresponding fixed points, strongly suggesting that (S10.1) is asymptotically stable, the numerical calculation of the Jacobian presented numerical issues which did not allow us to obtain a reliable estimate of its spectral radius. A potential explanation is the fact that (S10.1) is a complicated expression that also involves ODE integration, and our calculations steps were optimized for efficiency rather than accuracy.

Finally, we carried out the same test with the two-step FP maturation model, this time inferred using experimental data from the mScarlet-I fluorescent protein (see Figure S16 for measurements and inference results). The basins of attractions for each cell are outlined in Panel C of Figure S4. Again, the initial estimate $\hat{\boldsymbol{\beta}}^0$ lies inside the estimated basin of attraction of each cell. On the other hand, numerical issues prevented us from reliably estimating the spectral radius of the Jacobian.

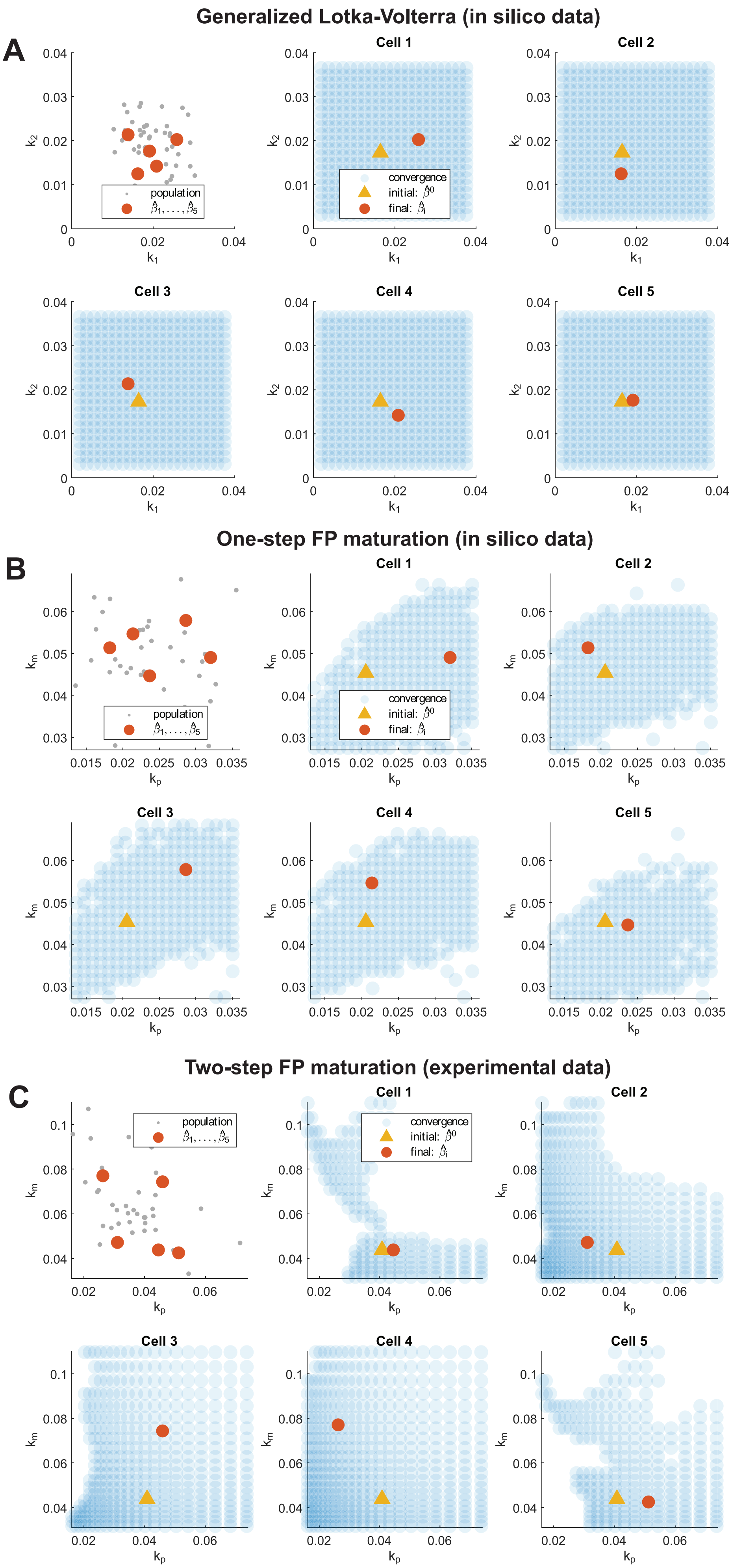

Figure S4: Basins of attraction for individual parameter estimates obtained by GMGTS for partially observed systems. In each panel, the first plot shows the collection of $N \approx 50$ first-stage parameter estimates $\hat{\beta}_i$, with the estimates of five selected cells $(\hat{\beta}_1, \ldots, \hat{\beta}_5)$ highlighted with orange disks. The remaining axes present sketches of the basin of attraction of the iterated map (S10.1) for $\hat{\beta}_1, \ldots, \hat{\beta}_5$. The initial point of the iterations used to produce all first-state estimates ($\hat{\beta}^0$, common for all cells in each panel) is marked with a yellow triangle. To approximate the basin of attraction, the iterations were initialized on a grid of points around the individual estimate. If the iterations ended up within a 5% error margin of the corresponding $\hat{\beta}_i$, the initialization point was marked blue. We used $T = 100$ time points and a multiplicative noise level of 0.1% to generate the *in silico* data; the experimental data are the ones reported in Figure S16. **A.** Basin of attraction sketches for the generalized Lotka-Volterra system using simulated data. For each $\hat{\beta}_i$, all tested initial points converge to the selected parameter estimate. **B.** Basin of attraction sketches for the one-step maturation model using simulated data. The initial point $\hat{\beta}^0$ of the iterations lies well within the estimated basins of attraction. **C.** Basin of attraction sketches for the two-step maturation model using experimental data from the mScarlet-I fluorescent protein. The grid is constructed in log-space since the inferred random effects distribution is log-normal. As in Panel B, the initial point $\hat{\beta}^0$ of the iterations lies within the sketched basins of attraction.

S11 Implementation details of GTS and GMGTS

This section outlines the software implementation details of the methodology presented in §S2. Note that cell subscript indices were omitted in some of the referenced equations for ease of reading; they are included here wherever applicable. These notes can serve as guidelines for future implementations of (GM)GTS.

We have implemented the GTS and GMGTS methods in Matlab using IQM Tools with Monolix for numerical integration. A user-ready implementation of these methods, as well as extensive documentation, can be found in Github [van Oppen, 2025]. We used the Symbolic Math Toolbox of Matlab to implement rudimentary preprocessing of a user-supplied IQM Tools model file containing differential equations, initial values, and nominal parameter values. From this file, our preprocessing code extracts the right-hand side **g** and **h** functions from (S2.6), as well as their partial derivatives with respect to the system states. This preprocessing is necessary to avoid the error-prone process of manually defining the different matrix-valued functions required by GMGTS. Since symbolic computation is slow, the derived symbolic functions are vectorized to handle multiple cells and time points and converted to regular Matlab functions. Doing so generates a small amount of overhead, so function calls are made for all cells at all time points rather than single cells or time points.

S11.1 Smoothing

Time series smoothing precedes parameter estimation in GMGTS. To facilitate and speed up this process, we implemented an interactive smoothing Matlab app (available on Github) to provide immediate visual feedback on the choice of the B-spline knot locations on the smooth trajectory estimates. The smooth estimates obtained via the app are directly fed into the gradient matching algorithm, making it easier to test the fidelity of the individual parameter estimates obtained with different smoothing settings.

The smoothing process starts by selecting the internal knots for the B-spline bases, which may be different from state to state. Our implementation includes a heuristic function `Smoother.place_knots()` (also available in the smoothing app) that automatically selects a set of internal knots for each state $k = 1, \ldots, L$. For clarity, we drop the subscript k from a measurement $y_{ik}(t_j) := y_i(t_j)$ for cell i, state k, and time point j. For each state $k = 1, \ldots, K$:

a. **Start with three equidistant internal knots by default.** Divide the smoothing interval into four equal intervals and to obtain three internal points. The measurement times closest to these points comprise the initial set of internal knots, after removing any duplicate points.

a. **Crudely estimate the gradient and curvature of each measured time series.** Estimate the gradient of each measured time series by means of finite difference approximation, i.e.,

$$\frac{dy_i}{dt}(t_j) \approx \widehat{dy}_{ij} := \frac{y_i(t_{j+1}) - y_i(t_{j-1})}{t_{j+1} - t_{j-1}} \quad \text{for} \quad j = 2, \ldots, T-1.$$

At each $j = 2, \ldots, T-1$, compute a normalized derivative approximation $\overline{d\mathbf{y}}_j$ from $\widehat{d\mathbf{y}}_{ij}$, $i = 1, \ldots, N$ by dividing the sample mean by the sample standard deviation (with respect to i).

Similarly, estimate the curvatures using

$$\frac{d^2y_i}{dt^2}(t_j) \approx \widehat{d^2y}_{ij} := \frac{2y_i(t_{j+1})}{(t_{j+1}-t_j)(t_{j+1}-t_{j-1})} - \frac{2y_i(t_j)}{(t_{j+1}-t_j)(t_j-t_{j-1})} + \frac{2y_i(t_{j-1})}{(t_{j+1}-t_{j-1})(t_j-t_{j-1})} \quad \text{for} \quad j = 2, \ldots, T-1,$$

to account for any unequal spacing in the measurement time points, and compute a normalized curvature approximation $\overline{d^2\mathbf{y}}_j$ analogous to $\overline{d\mathbf{y}}_j$.

b. **Add knots near peaks and troughs.** Locate the points where $\overline{dy}_j$ crosses zero and $\overline{d^2y}_j$ is sufficiently far from zero (to avoid adding excessive points where the derivative is mostly flat, we have used a threshold of 0.25 in terms of absolute value). Extend the set of internal knots with the measurement time points closest to the crossing points, removing existing knots if they end up on adjacent measurement time points.

c. **Add a knot at the end of initial fast dynamics (if any).** To accommodate systems with initial fast dynamics, find the first measurement point where the normalized curvature $\overline{d^2y}_j$ does not exceed a certain threshold for at least two subsequent measurement times. We have found that setting this threshold to two times the sample standard deviation of $\overline{d^2y}_j$ with respect to j works well for the systems we considered. Add this measurement time point to the set of internal knots, again removing existing knots if they end up on adjacent measurement time points.

For each system in our simulation studies and inference from experimental data, we used the knot placement heuristic together with some small manual corrections in the interactive smoothing app on a few representative cases to obtain a single selection of knots. This selection was kept *constant* under all tested conditions and across all cells to prevent additional variance in the recorded accuracies and computing times. Table S1 lists the set of knots obtained in this way for each system. While adjusting the knots per tested condition would have produced better accuracy in some cases, keeping them constant also demonstrates the robustness of GMGTS with respect to the smoothing step. To illustrate how the smoothing heuristic performs against manually curated knots, Figure S5 shows smoothed measurements using both approaches for three representative cases. Normalized Wasserstein distances

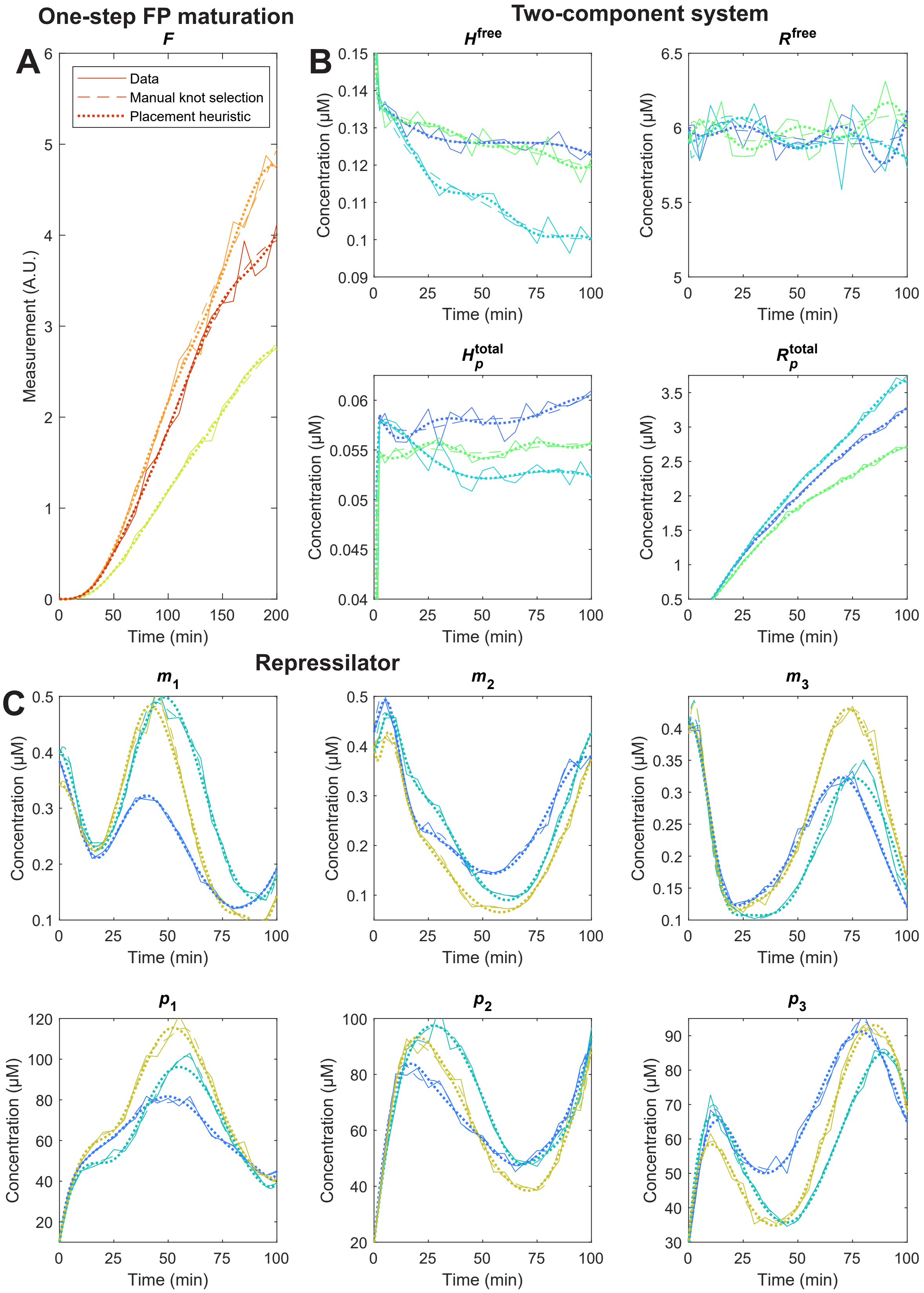

Figure S5: Comparison of smoothing using manually selected knots and the knot placement heuristic. *In silico* measurement data for the different systems using representative parameter settings, smoothed in two ways: via manually selected knots (dashed, cf. Table S1) and with the knot placement heuristic (dotted, cf. Steps a–d in § S11.1). The smoothed measurements produced by both strategies were used infer population distributions using GMGTS; the normalized Wasserstein distances to the data-generating distribution are reported for comparison. **A.** Smoothed measurements for the one-step FP maturation model with multiplicative noise level $\tau = 5\%$. The normalized Wasserstein distances (see § S11.5 to the data-generating distribution were 0.0791 for the manually selected knots versus 0.0824 for the knot placement heuristic. **B.** Smoothed measurements for the bifunctional two-component system with multiplicative noise level $\tau = 2\%$. The normalized Wasserstein distances to the data-generating distribution were 0.1050 for the manual selection versus 0.1064 for the heuristic. **C.** Smoothed measurements for the repressilator system with multiplicative noise level $\tau = 5\%$. The normalized Wasserstein distances (see §S11.5 to the data-generating distribution were 0.0489 for the manual selection versus 0.0988 for the heuristic. Although the Wasserstein distances differ more in this case, distances below 0.1 are minor from a practical point of view (as indicated in Figure S6). Altogether, the smoothing heuristic provides a good starting point for GMGTS inference in all tested cases.

In silico data			
Two-component system	One-step FP maturation	Repressilator	Generalized Lotka-Volterra
[0, 2.5, 40, 100]	[0, 10, 20, 60, 120, 200]	[0, 10, 20, . . . , 100]	[0, 5, 10, 15, 20]

Experimental data	
One-step maturation	Two-step maturation
[0, 10, 20, 40, 80, 100]	[0, 10, 20, 40, 80, 200]

Table S1: Selected system-specific B-spline knots. Sets of B-spline basis knots selected for each system using the knot placement heuristic and manual fine-tuning in the interactive smoothing app.

to the data-generating distributions are listed in the caption, indicating the heuristic does not lead to drastically lower accuracy when used to infer random effect distributions.

For the iterative estimation of noise parameters (cf. § S6), all measurement noise parameters are initialized at $\sigma_k = 1$ and $\tau_k = 0$; that is, we start by assuming homoskedastic errors. The spline basis matrices for each state are precomputed for computational efficiency. Then, the smoothing proceeds iteratively:

1. Update the spline coefficient vector estimate $\hat{\boldsymbol{\delta}}_i$ using (S6.2) and compute the corresponding estimated state values $\hat{x}_{ik}(t_j)$.

2. Optimize the log-likelihood (S2.8) with $\hat{x}_{ik}(t_j)$ in place of $x^*_k(t_j; \boldsymbol{\beta}_i)$ to update each $\hat{\sigma}_k$ and $\hat{\tau}_k$, initializing the optimization at the non-negative least squares estimate (S6.3). In our Matlab implementation, we use the interior point algorithm provided by `fmincon()` for the log-likelihood maximization and terminate when the relative step size drops below 1E-8.

3. Repeat steps 1 and 2 until convergence.

When the procedure ends, use $\hat{\boldsymbol{\delta}}$ to compute each $\hat{x}_{ik}(t)$ and $\hat{\dot{x}}_{ik}(t)$ to be used in gradient matching on a minimal set of time points t (not necessarily the measurement times) that still capture the dynamics of the system. Note the smoothed states and gradients at the first and last time points should be omitted as they often carry more bias than other points. We have found that dividing the smoothing interval into 10 equidistant segments and selecting the 9 interior points (to omit the endpoints) for optimization yields reliable first-stage estimates across the systems we tested. When the data are already sparse, it suffices to retain the $\hat{x}_{ik}(t)$ and $\hat{\dot{x}}_{ik}(t)$ on the measurement time points. Finally, also compute (S6.1) using $(\hat{\sigma}_k, \hat{\tau}_k)$ and with $\hat{x}_{ik}(t_j)$ in place of $x^*_k(t_j; \boldsymbol{\beta}_i)$.

S11.2 Individual gradient matching estimates with full observation

Initialize the residual covariance matrices $\operatorname{Var} \boldsymbol{\Delta}_i$ as $TK \times TK$ identity matrices, starting the FGLS iterations with homoskedastic errors. Then,

1. Update each $\hat{\boldsymbol{\beta}}_i$ by minimizing the GSS criterion in (S2.14) using quadratic programming to conform with parameter bounds (cf. § S2.3). Our Matlab implementation makes use of the `mpcActiveSetSolver()`, which is based on [Schmid and Biegler, 1994].

2. Compute $\operatorname{Var} \boldsymbol{\Delta}_i = \hat{\mathbf{V}} = \mathbf{V}(\hat{\boldsymbol{\beta}}_i)$ using (S7.6). This matrix may be rank-deficient due to the small number of knots versus time points. In case it is not of full rank, wherever we need its inverse, we supply its Moore-Penrose pseudoinverse [Horn and Johnson, 2012] instead.

3. Repeat steps 1 and 2 until convergence.

While cell-specific parameter estimates can in principle be computed sequentially, the preprocessing step described at the start of this section makes it significantly more efficient to make calls to right-hand side functions $\mathbf{g}$ and $\mathbf{h}$ (defined in (S2.6)) in batches. In this way, we can simultaneously update all individual parameter estimates that have not converged at each FGLS step. Since the random effects distribution rather than individual parameter estimates is the object of interest in our method, it suffices to use a relatively loose termination criteria for individual estimates. One possibility is to terminate the iterations of a cell when the relative change in the parameter vector magnitude is smaller than 1%, and terminate the first stage altogether when ~90% of the cell estimates have converged according to this criterion. Another possibility is to terminate the FGLS iterations for all cells after a fixed, empirically determined number of updates (e.g. 5–10) which ensures that nearly all individual estimates have converged. In the examples presented here, the second approach was used with 5 iterations for all systems except the FP maturation model, where 10 iterations were used.

S11.3 Individual gradient matching estimates with partial observation

For the iterative estimation scheme described in § S2.4, we initialize all cell-specific parameter estimates $\hat{\boldsymbol{\beta}}^0_i$ at the same value. To do this, we minimize the discrepancy between the ODE model prediction for a single parameter value and the mean trajectory data across cells. That is, we estimate

$$\hat{\boldsymbol{\beta}}^0_1 = \cdots \hat{\boldsymbol{\beta}}^0_N := \arg\min_{\boldsymbol{\beta}} = \sum_{k=1}^{K} \sum_{j=1}^{T} w_k^2 \left(\bar{y}_k(t_j) - x^*_k(t_j; \boldsymbol{\beta})\right)^2, \quad \text{where} \quad \bar{y}_k(t_j) := \sum_{i=1}^{N} y_{ik}(t_j).$$

The state-specific weights w_k in this sum of squares above may simply be taken to all equal 1 or be inversely proportional to the state-specific measurement averages across cells and time points.

To perform the minimization, we use the interior point algorithm provided by `fmincon()` in Matlab, multi-started at 10 points randomly sampled within the parameter bounds and terminated when the relative step size falls below 1E-4. As noted in § S8, low estimation precision should be attributed to $\hat{\beta}^0$. We have found that, in the absence of prior knowledge, a conservative estimate using a diagonal matrix with a CV of $c = 0.5$ suffices. Specifically, in this case we initialize

$$\operatorname{Var}\hat{\beta}^0 = \operatorname{diag}(c\hat{\beta}^0)^2.$$

As discussed in § S2.4, contrary to the case of full observation, we first produce estimates of the hidden states by integrating the ODE system for each cell using $\hat{\beta}_i^0$. We then estimate the initial residual covariance matrix $\hat{\mathbf{V}}_0$ using (S8.4), store the intermediate estimate of $\operatorname{Var}[\hat{\mathbf{X}}_0, \hat{\dot{\mathbf{X}}}_0]$, and at each iteration $J \geq 1$:

1. Compute each updated $\hat{\beta}_i^J$ by minimizing (S2.14) using quadratic programming, as with full observation.
2. Numerically integrate the ODE system using each $\hat{\beta}_i^J$ to update the hidden components of the state and gradient estimates $\hat{\mathbf{X}}_J, \hat{\dot{\mathbf{X}}}_J$.
3. Compute the updated $\hat{\mathbf{V}}_J$ using (S8.4). To do so, first compute the updated estimate of $\operatorname{Var}[\hat{\mathbf{X}}_J, \hat{\dot{\mathbf{X}}}_J]$ using (S7.14), (S8.6), (S8.7), and the stored value of $\operatorname{Var}[\hat{\mathbf{X}}_{J-1}, \hat{\dot{\mathbf{X}}}_{J-1}]$ from the previous iteration. When using maximum a posteriori estimates in Step 1, take note of the modifications listed in (S9.2) and (S9.3).
4. Analogously to the case of full observation, repeat steps 1–3 until convergence or until the maximum number of iterations has been reached. Upon termination, approximate $\operatorname{Var}\hat{\beta}$ using (S8.8).

In our tests, allowing for at most 5 or 10 iterations (depending on the system tested) was enough to let the parameter estimates converge for the majority of the population. To evaluate (S8.4) and (S8.6), ODE solution sensitivities with respect β need to be approximated. For the calculation of these sensitivities, central finite-difference approximations with decreased integration tolerance (1E-4 versus 1E-6 used by default) to increase efficiency turned out to be more computationally efficient than solving the forward sensitivity equations in IQM Tools and Monolix, adding $2P$ numerical ODE integrations per iteration (where P is the dimension of each $\hat{\beta}_i^J$). The consideration regarding the organization and termination of the iterations of individual parameter estimates, discussed at the end of § S11.2, also applies in the case of partial observation.

S11.4 GTS settings used for performance comparisons

We aimed to implement the GTS approach as efficiently as possible, and used the same estimator settings with GMGTS wherever applicable (e.g. in the second stage, where 10 EM iterations were used for both methods).

Wherever numerical optimization was needed in GTS, we used the interior-point method provided by the `fmincon()` function in Matlab.

In the first GTS stage, an FGLS iteration for an individual cell comprises an a non-convex optimization step followed by second optimization step to estimate the noise variance parameters (cf. § S2.2). As in GMGTS, 5 FGLS iterations were used for all models with the exception of the FP maturation, where 10 iterations were used.

Similar to GMGTS with partial observation, the first-stage individual estimates of GTS were initialized by matching the average of the single-cell measurements using multi-started optimization from 10 starting points to yield a common initial estimate for all cells. The optimization for each starting point was terminated using a relative step tolerance of 1E-4. At each subsequent FGLS iteration, the non-convex optimization routine that updates individual parameter estimates was initialized at the estimate of the previous step to speed up convergence. To terminate the optimization of each individual parameter estimate, a relative step tolerance of 1E-8 was used. Likewise, a tolerance of 1E-8 was used for estimating measurement noise parameters via optimization, as described at the end of § S2.2.

Note that the default relative step tolerance of `fmincon()` is 1E-10. To speed up the GTS method, we were able to increase this tolerance by two orders of magnitude. Further increase caused the accuracy of GTS to fall significantly below that of GMGTS for some systems.

Finally, analogously to [Dharmarajan et al., 2019], we approximated the uncertainty of each first-stage estimate with the inverse of the Fisher information matrix, ignoring second-order terms. This approximation is based on ODE solution sensitivities with respect to the individual parameter estimate. As mentioned at the end of § S11.3, these sensitivities are best calculated using finite-difference approximations.

S11.5 Wasserstein distance between multivariate normal distributions

Given two non-degenerate multivariate normal distributions $\mathrm{N}_1(\mu_1, \Sigma_1)$ and $\mathrm{N}_2(\mu_2, \Sigma_2)$, the Wasserstein distance with respect to the Euclidean norm $\|\cdot\|_2$ is given by the formula [Givens and Shortt, 1984]

$$W_2(\mathrm{N}_1, \mathrm{N}_2) = \|\mu_1 - \mu_2\|_2^2 + \operatorname{tr}\left(\Sigma_1 + \Sigma_2 - 2\left(\Sigma_2^{1/2}\Sigma_1\Sigma_2^{1/2}\right)^{1/2}\right). \tag{S11.1}$$

As (S11.1) shows, the Wasserstein distance depends on the absolute values of the means and covariances of the two distributions. In the examples considered in this work, one of the two normal distributions is a reference (ground-truth) distribution, while the other

one is an estimate that should approximate the reference. To remove the scale dependence and give equal weight to marginals that differ in scale, we can normalize the two distributions in such a way that the all marginals of the reference distribution (say N_1) have a mean of 1. This is equivalent to using a scaling matrix $C = \text{diag}(\mu_{1,1}^{-1}, \ldots, \mu_{1,p}^{-1})$ to get $N_1(C\mu_1, C\Sigma_1 C)$ and $N_2(C\mu_2, C\Sigma_2 C)$. The Wasserstein distances reported in the main text plots are the distances between these normalized distributions.

To aid the interpretation of these results, Figure S6 displays different pairs of normal distributions and their Wasserstein distances, calculated following the normalization approach outlined above.

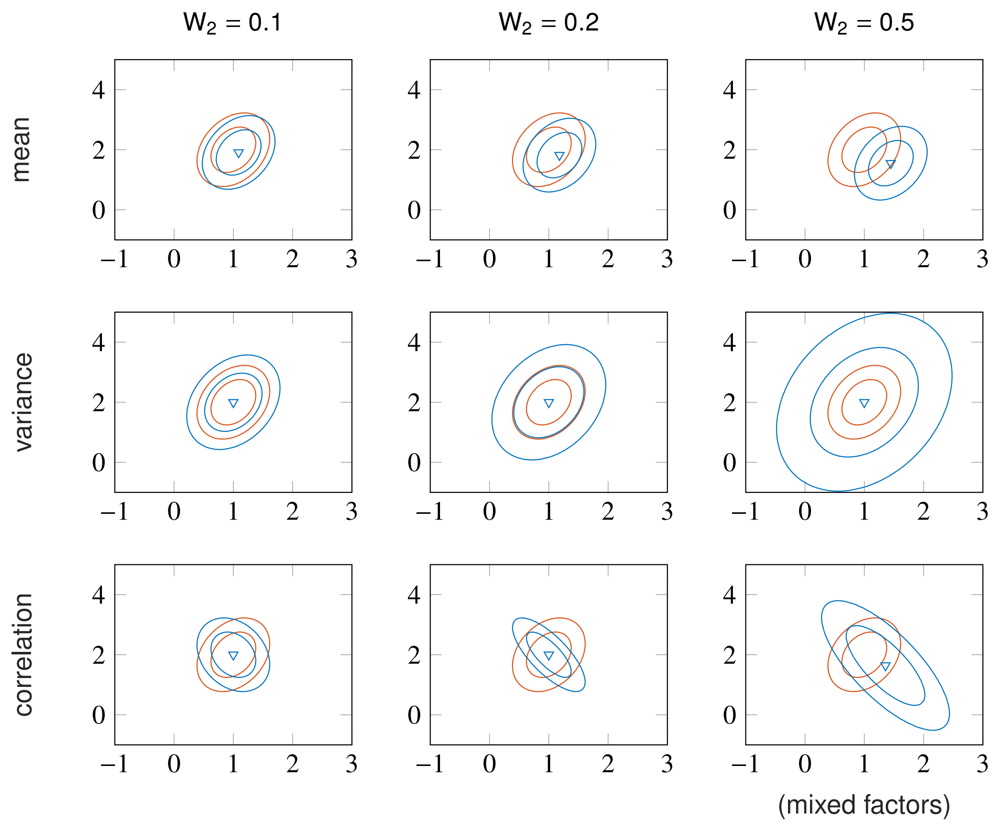

Figure S6: Normalized Wasserstein distances for various differences in distributions Differences in means *(top row)*, variances *(middle row)*, and correlation *(bottom left and middle panels)* at three normalized Wasserstein distances W_2 *(one value per column)*. The reference (ground-truth) distribution, displayed in orange, was held constant across scenarios. Distinct differences in distributional aspects may each contribute to the Wasserstein distance to the reference distribution *(bottom right panel)*.

S12 Figures related to simulation studies

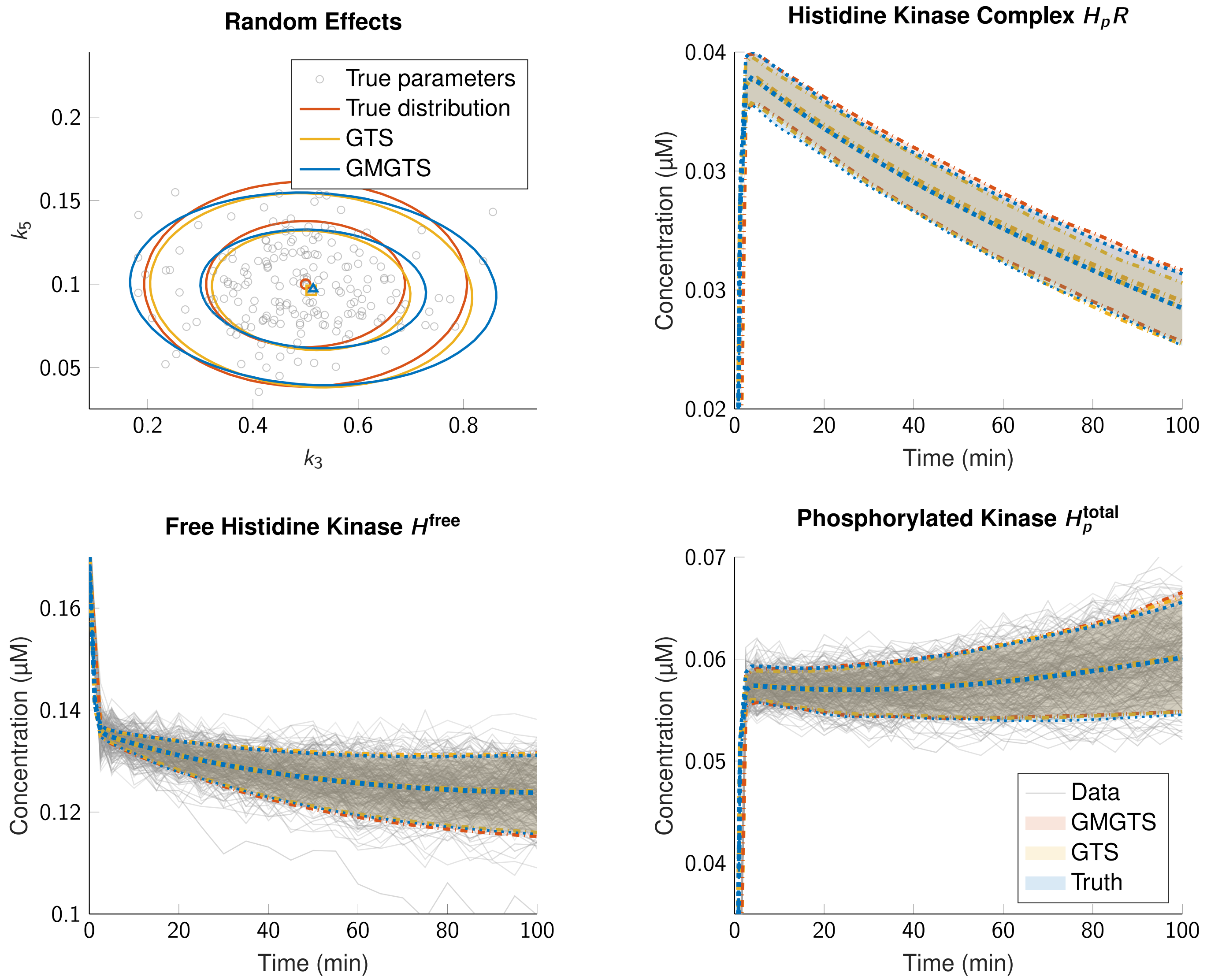

Figure S7: Simulation study of the bifunctional two-component system with partial observation: examples of inference and prediction results. *Top left panel:* example of a (marginal) random effects distribution inferred by the GMGTS and GTS methods, compared to the true data-generating distribution. Distributions are summarized by their mean and 68% and 95% contour levels. *Remaining panels:* predicted distributions of some unobserved and observed states (unphosphorylated histidine kinase H, phosphorylated response regulator-kinase complex R_pH, and total phosphorylated histidine kinase H_p^{total}), summarized by their mean and 90% confidence intervals of the state distributions. The simulated measurement data for H_p^{total} are also included in the bottom-right panel. The (multiplicative) noise level was set at $\tau = 3\%$ and the data were simulated at $T = 22$ time points. The attained normalized Wasserstein distances of the inferred random effects distributions from the ground-truth are 0.1050 versus 0.0957 for GMGTS versus GTS, respectively.

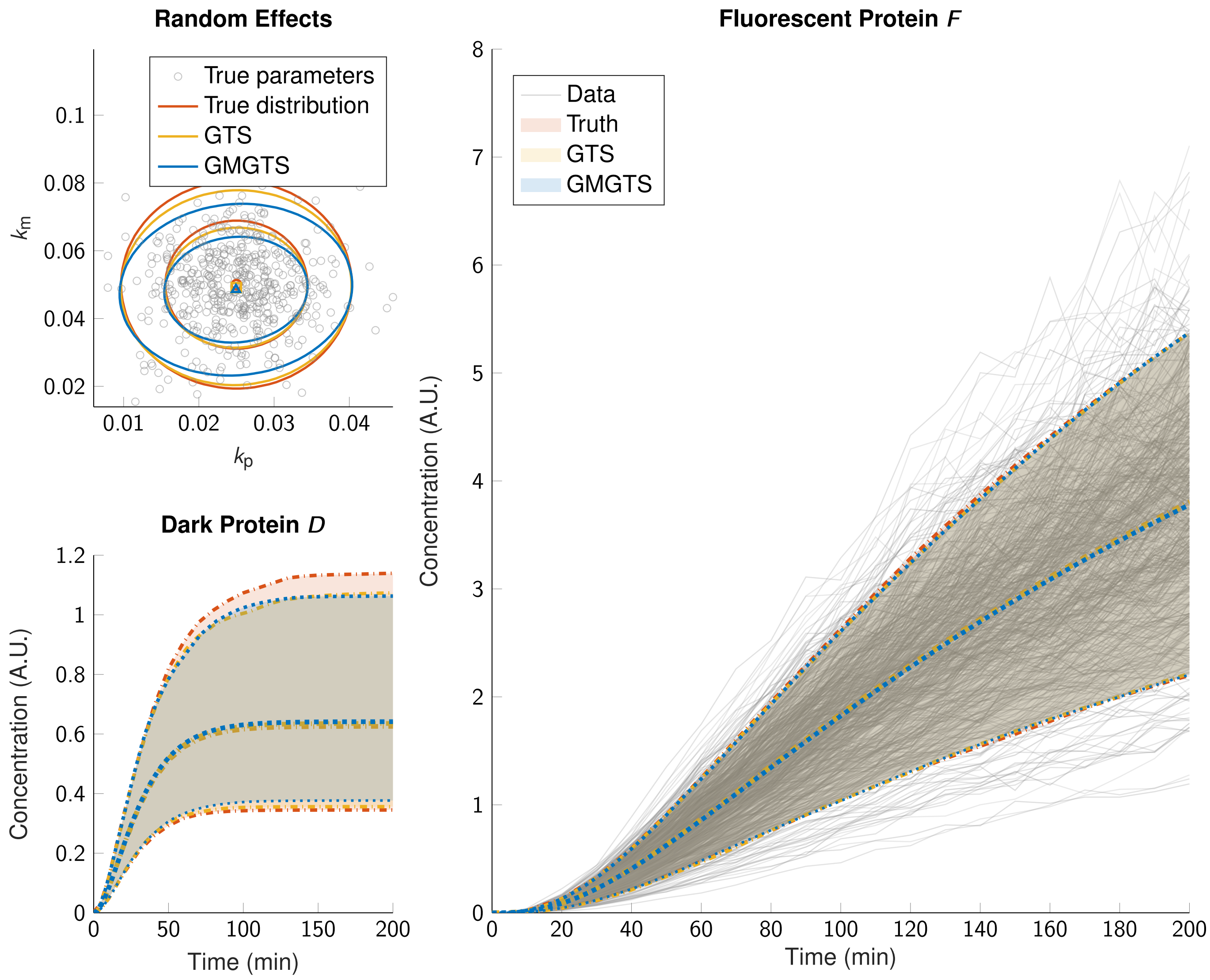

Figure S8: Simulation study of FP maturation dynamics: illustration of inference and prediction results. *Top left panel:* random effects distributions inferred by the GMGTS and GTS methods compared to the true data-generating distribution, summarized by their mean and 68% and 95% contour levels. *Remaining panels:* predicted state distributions (immature and mature FP), summarized by their mean and variability (90% confidence intervals of the state distributions). The simulated measurement data for F (mature FP) are also included in the right panel. The (multiplicative) noise level was set at $\tau = 3\%$ and the data were simulated at $T = 22$ time points. The attained normalized Wasserstein distances of the inferred random effects distributions from the ground-truth are 0.0551 versus 0.0244 for GMGTS versus GTS, respectively.

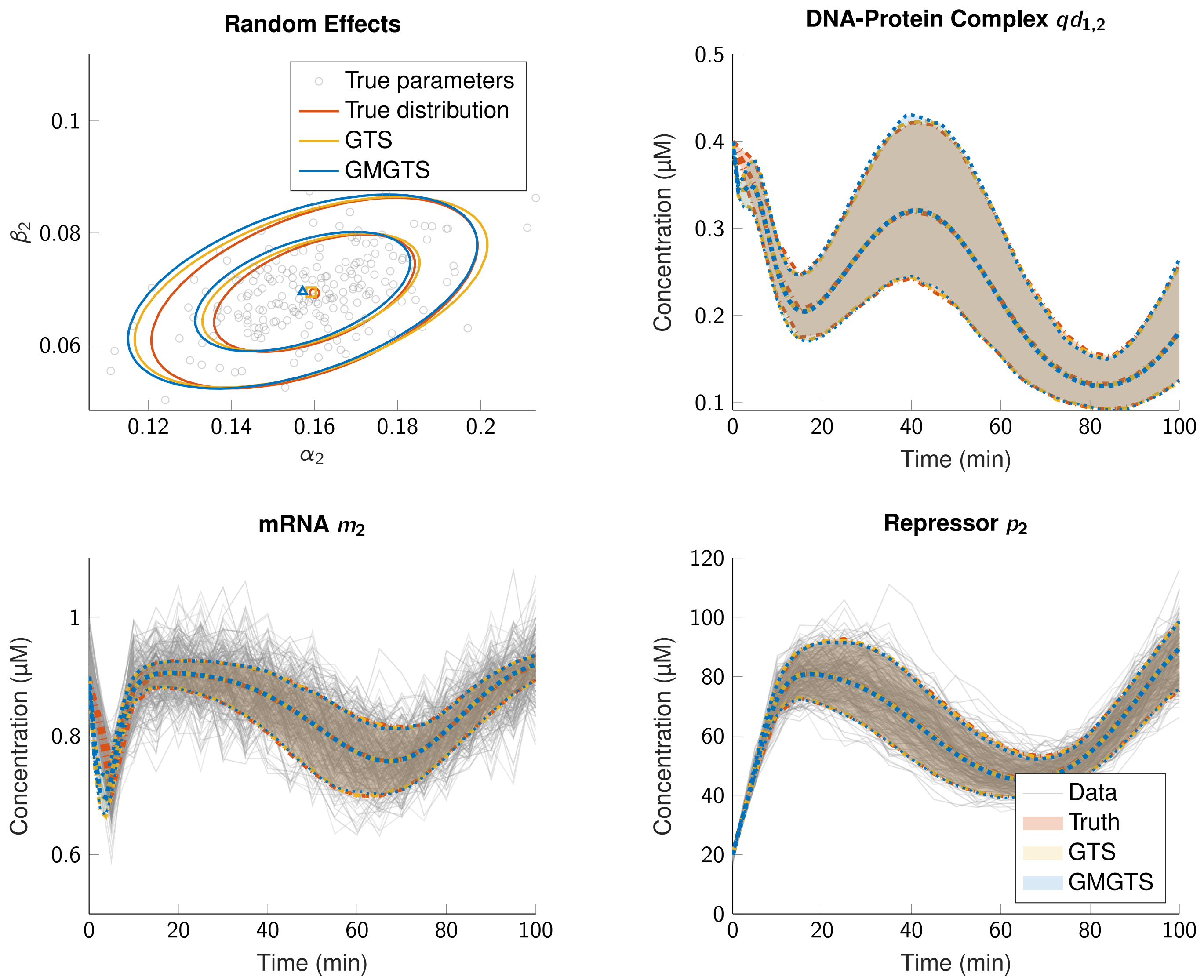

Figure S9: Simulation study of the repressilator: illustration of inference and predictions results. *Top left panel:* selected random effects distributions inferred by the GMGTS and GTS methods compared to the true data-generating distribution, summarized by their mean and 68% and 95% contour levels. *Remaining panels:* predicted state distributions (DNA-protein complex $qd_{1,2}$, mRNA m_1, and repressor p_1 concentrations) summarized by their mean and variability (90% confidence intervals of the state distributions). The selected states directly influence the concentration of the first repressor in the system (p_1), and similar results are obtained for the remaining ones. The simulated measurement data for m_1 and p_1 are also included in the bottom panels, where the (multiplicative) noise level was set at $\tau = 5\%$ and the data were simulated at $T = 21$ time points. The attained normalized Wasserstein distances of the inferred random effects distributions from the ground-truth are 0.0413 versus 0.0277 for GMGTS versus GTS, respectively.

S13 Results related to the inference of FP maturation rates

S13.1 Neglecting dilution rate variability does not significantly inflate the variability of maturation rate estimates

In the data from [Guerra et al., 2022], protein dilution at the single-cell level is driven by the increase in cell volume over the mother cell division cycle (∼100 min). Because both volume growth and division timing vary across cell cycles —and our measurements often span multiple cycles— it is not straightforward to define a unique dilution rate for each cell. To address this, we assumed a common dilution rate for all cells within a given experiment.

To understand how our simplifying assumption of a common dilution rate for all cells could affect parameter inference, we generated *in silico* measurement data, assuming that the dilution rate has a known mean (as in our model), but is also affected by cell-to-cell variability with a coefficient of variation of 0.05, a value that agrees with the experimental evidence of [Guerra et al., 2022]. We then used a model with a fixed (i.e. non-variable) dilution rate to infer the (normal) random effects distribution of k_p and k_m, assuming a coefficient of variation of 0.25 for the maturation rate. Table S2 summarizes the inferred maturation rate medians and quartiles from ten independent runs of data generation and inference. These results suggest that neglecting the variability of the dilution rate during inference does not significantly inflate the variability of maturation rate estimates, as long as the maturation rate variability is considerably larger than the variability in dilution rates.

Maturation model	k_{m}	k_{m} CV (Fixed k_{dil}) Median	$q_{0.25}$	$q_{0.75}$	k_{m} CV (Variable k_{dil}) Median	$q_{0.25}$	$q_{0.75}$
One-step	**0.025**	0.218	*0.209*	*0.222*	0.223	*0.210*	*0.229*
	**0.050**	0.222	*0.219*	*0.231*	0.228	*0.220*	*0.234*
Two-step	**0.025**	0.213	*0.210*	*0.220*	0.219	*0.212*	*0.225*
	**0.050**	0.227	*0.221*	*0.235*	0.231	*0.223*	*0.240*

Table S2: Simulation study on ignoring dilution rate variability in the FP maturation system. Synthetic data were generated under different scenarios (fixed versus variable k_{dil} and two different k_m means) and were used to infer the population distribution of k_m, assuming multiplicative measurement noise with $\tau = 4\%$. The variability of the inferred distribution (summarized by the coefficient of variation) was then compared to the variability of the ground-truth (data-generating) distribution of k_m. The nominal value $k_{\mathrm{dil}} = 0.004$ and corresponding variability in terms of a coefficient of variation (CV) of 0.05 were inferred from [Guerra et al., 2022]. *Second column:* k_{m} mean used to generate the data; each data-generating distribution of k_{m} had a CV of 0.25. *Remaining columns*: summaries of the CVs of the inferred k_m distributions for 10 independent repetitions of data generation and inference using medians and quartiles. Overall, there is no indication that treating the dilution rate as fixed when it is actually variable leads to significantly inflated variability in the maturation rate estimates.

S13.2 Comparison of FP maturation models with common vs. variable maturation rate

To further verify whether a variable maturation rate is supported by the data of each FP, we fitted a mixed-effects model with a common maturation rate for all cells (fixed at the average maturation rate of each FP) and performed a statistical comparison of this model against the model with cell-specific maturation rates. To obtain the former model, we fixed the maturation rate of each FP to its average (Table S4) and inferred the distribution of k_p from the single-cell data. We then calculated and compared the marginal likelihoods of the two models, listed in Table S3. For every FP, the model with fixed maturation rate had a substantially lower log-likelihood compared to the model with variable maturation, suggesting that the data do not support fixed maturation rates.

Marker	$\log L$ (k_{m} fixed)	$\log L$ (k_{m} variable)	Marker	$\log L$ (k_{m} fixed)	$\log L$ (k_{m} variable)
sfGFP	-256339	-349175	CFP	-3090	-1117
mCitrine	-5628	1235	mTurquoise2	-15344	-9809
mNeonGreen	-17607	-10982	mCherry	-126	3537
mVenus	-5098	-1950	mTFP1	-48361	-342
pHtdGFP	-19378	-7672	tdTomato	-55538	2894
mScarletI	296	1385	mKate2	-57851	3169

Table S3: Marginal likelihood values of FP maturation models with fixed vs. variable maturation rate. *Middle columns:* marginal log-likelihoods of a model with variable k_{p} and fixed k_{m} fixed. *Right columns:* marginal log-likelihood values of a model with variable k_{p} and k_{m}. Likelihoods are many orders of magnitude larger when k_{m} is considered variable across a population. Since all likelihood differences are substantial, the differences in degrees of freedom between the two models can be safely neglected; the model with variable maturation rate is considerably more likely given the experimental data.

S14 Additional results on the inference of variable FP maturation rates

Marker	t_{50} [Guerra et al., 2022]	Mean $t_{1/2}$ (GMGTS)	$q_{0.25}$	$q_{0.75}$	RQR
sfGFP	6.9	6.6	5.2	13.4	0.936
mCitrine	12	7.9	6.3	14.7	0.792
mNeonGreen	12	9.3	7.5	14.4	0.561
pHtdGFP	12.4	9.9	8	17.4	0.709
mVenus	13.9	10.2	8.4	14.6	0.458
CFP	8.3	12.1	9.7	21.1	0.708
mScarletI	28.9	26.7	22.4	36.2	0.386
mTurquoise2	46.6	40.9	34.4	54.8	0.373
mCherry	49.4	46	38.8	60.9	0.36
mTFP1	79.9	75.5	66.5	90.3	0.236
tdTomato	104.9	90.3	76.7	117.7	0.34
mKate2	111.9	112.3	100.5	130.2	0.198

Table S4: Summary of inferred maturation half time distributions and comparison with previously published results. *Second column:* maturation half-times (in minutes) estimated by [Guerra et al., 2022] for each fluorescent protein. *Third column:* mean half-times obtained from the GMGTS method. *Remaining columns:* summaries of the maturation half-time distributions by their 25% and 75% quartiles, as well as their relative quartile range (RQR). The latter is defined as the interquartile range divided by the mean and multiplied by 0.75 (making it comparable to the coefficient of variation for normal distributions). Faster-maturing proteins tend to have higher relative variability in terms of RQR.

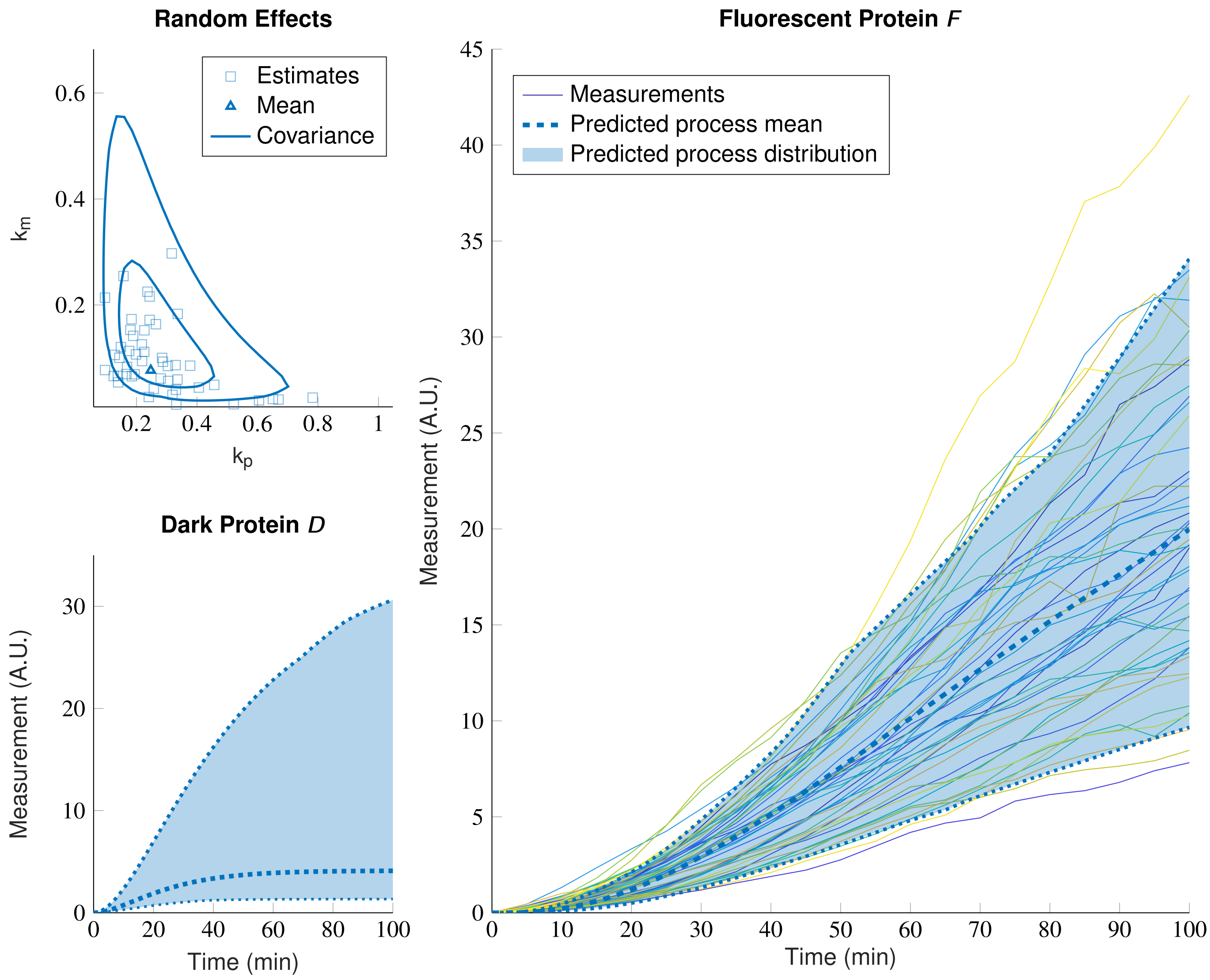

Figure S10: sfGFP: parameter inference and state predictions. *Top left panel:* individual estimates of k_p and k_m produced by the first stage of GMGTS (squares) and the associated random effects distribution obtained from the second stage, summarized by its mean and 68% and 95% contour levels. *Remaining panels:* Predicted distributions of immature and mature FP, summarized by their mean and variability (90% confidence intervals of the state distributions). The (preprocessed) experimental data are also included in the right panel.

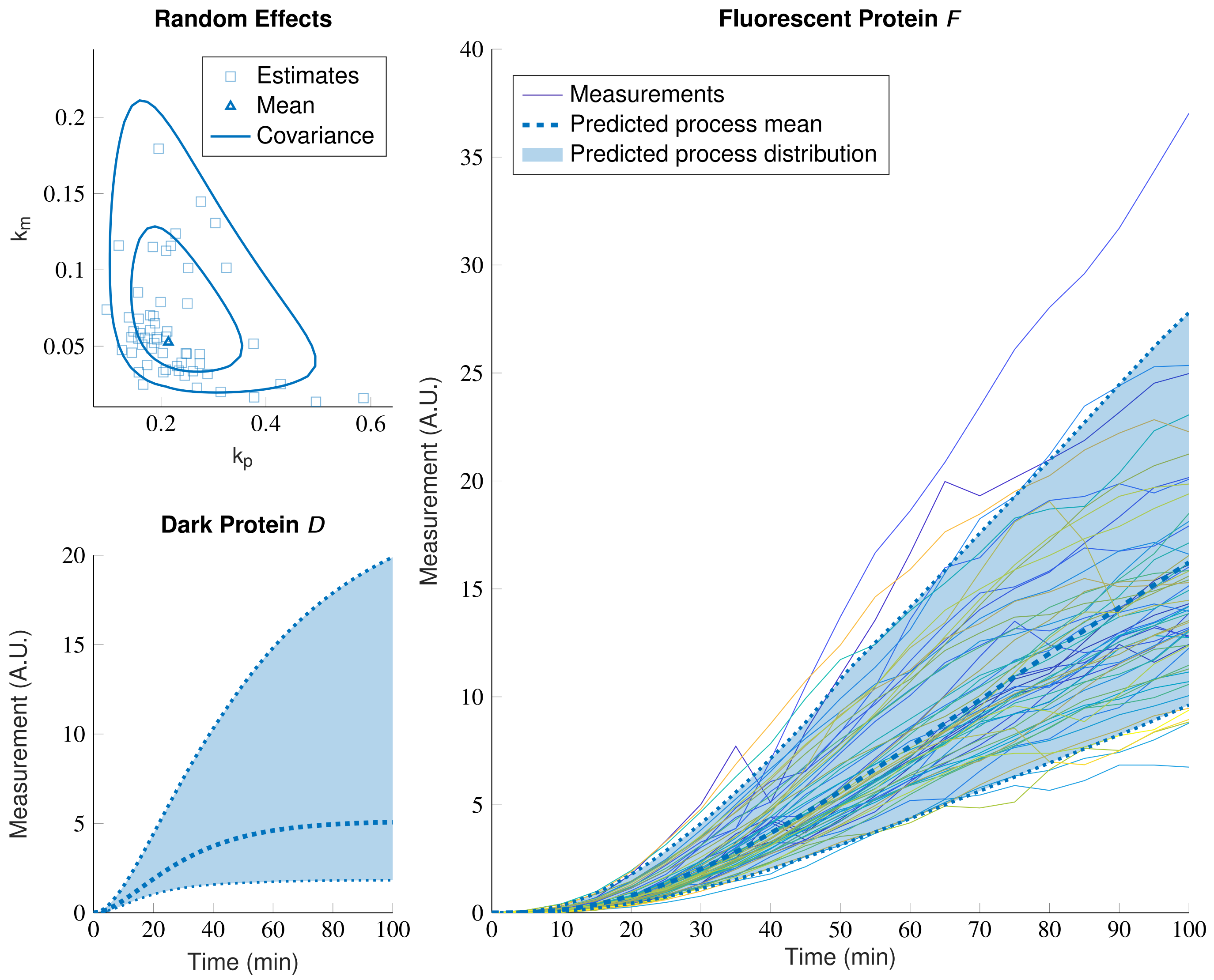

Figure S11: CFP: parameter inference and state predictions. *Top left panel:* individual estimates of k_p and k_m produced by the first stage of GMGTS (squares) and the associated random effects distribution obtained from the second stage, summarized by its mean and 68% and 95% contour levels. *Remaining panels:* Predicted distributions of immature and mature FP, summarized by their mean and variability (90% confidence intervals of the state distributions). The (preprocessed) experimental data are also included in the right panel.

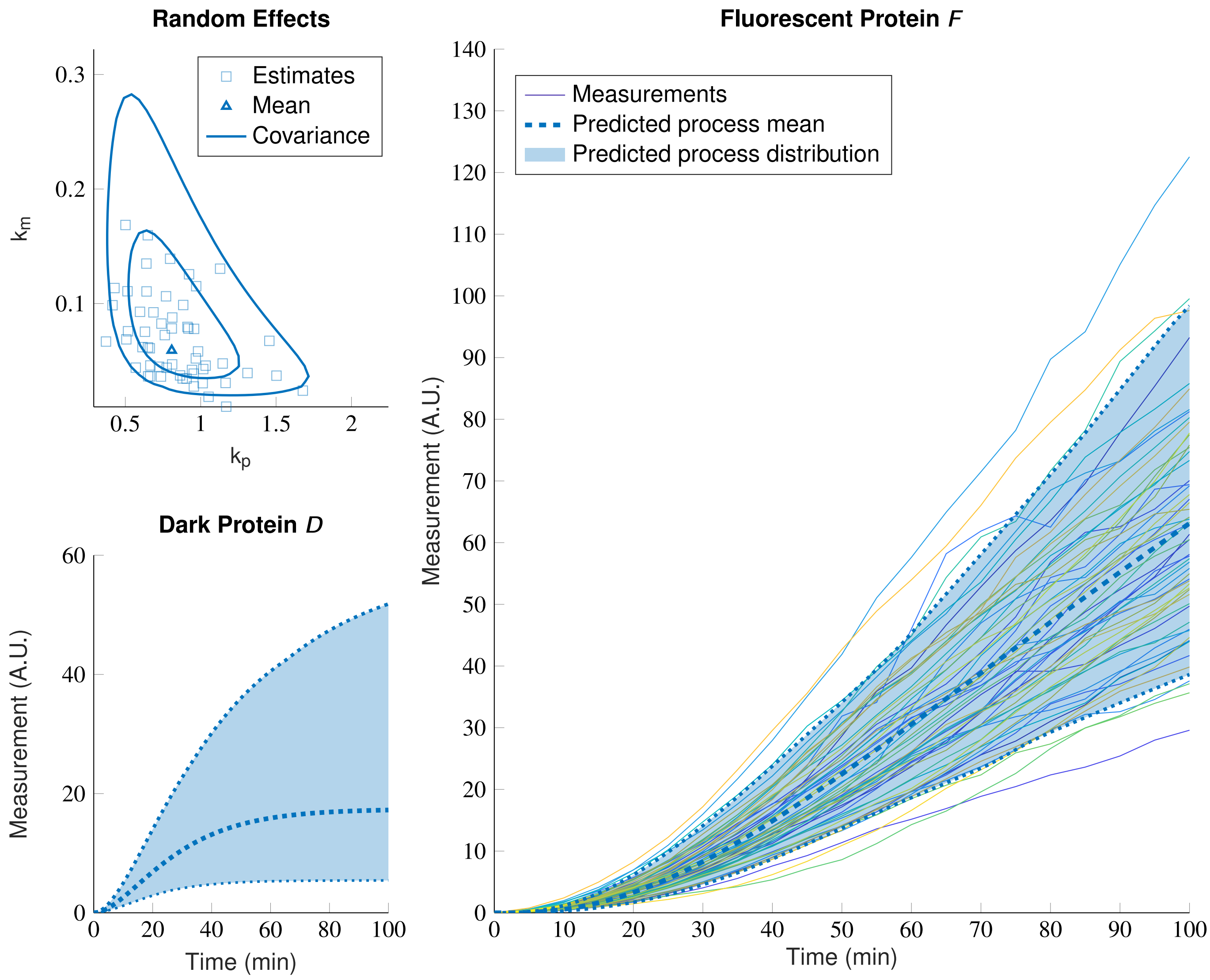

Figure S12: pHtdGFP: parameter inference and state predictions. *Top left panel:* individual estimates of k_p and k_m produced by the first stage of GMGTS (squares) and the associated random effects distribution obtained from the second stage, summarized by its mean and 68% and 95% contour levels. *Remaining panels:* Predicted distributions of immature and mature FP, summarized by their mean and variability (90% confidence intervals of the state distributions). The (preprocessed) experimental data are also included in the right panel.

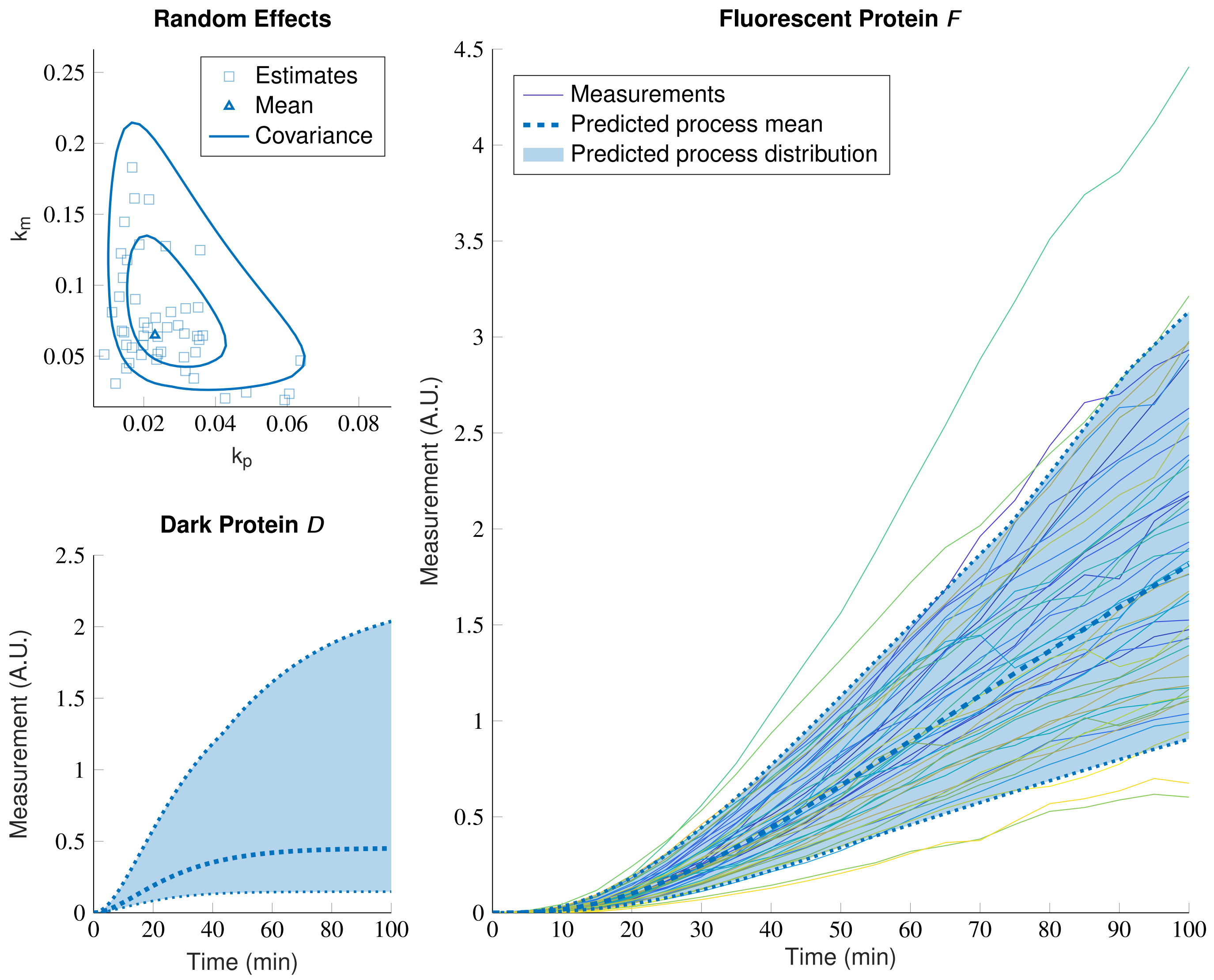

Figure S13: mNeonGreen: parameter inference and state predictions. *Top left panel:* individual estimates of k_p and k_m produced by the first stage of GMGTS (squares) and the associated random effects distribution obtained from the second stage, summarized by its mean and 68% and 95% contour levels. *Remaining panels:* Predicted distributions of immature and mature FP, summarized by their mean and variability (90% confidence intervals of the state distributions). The (preprocessed) experimental data are also included in the right panel.

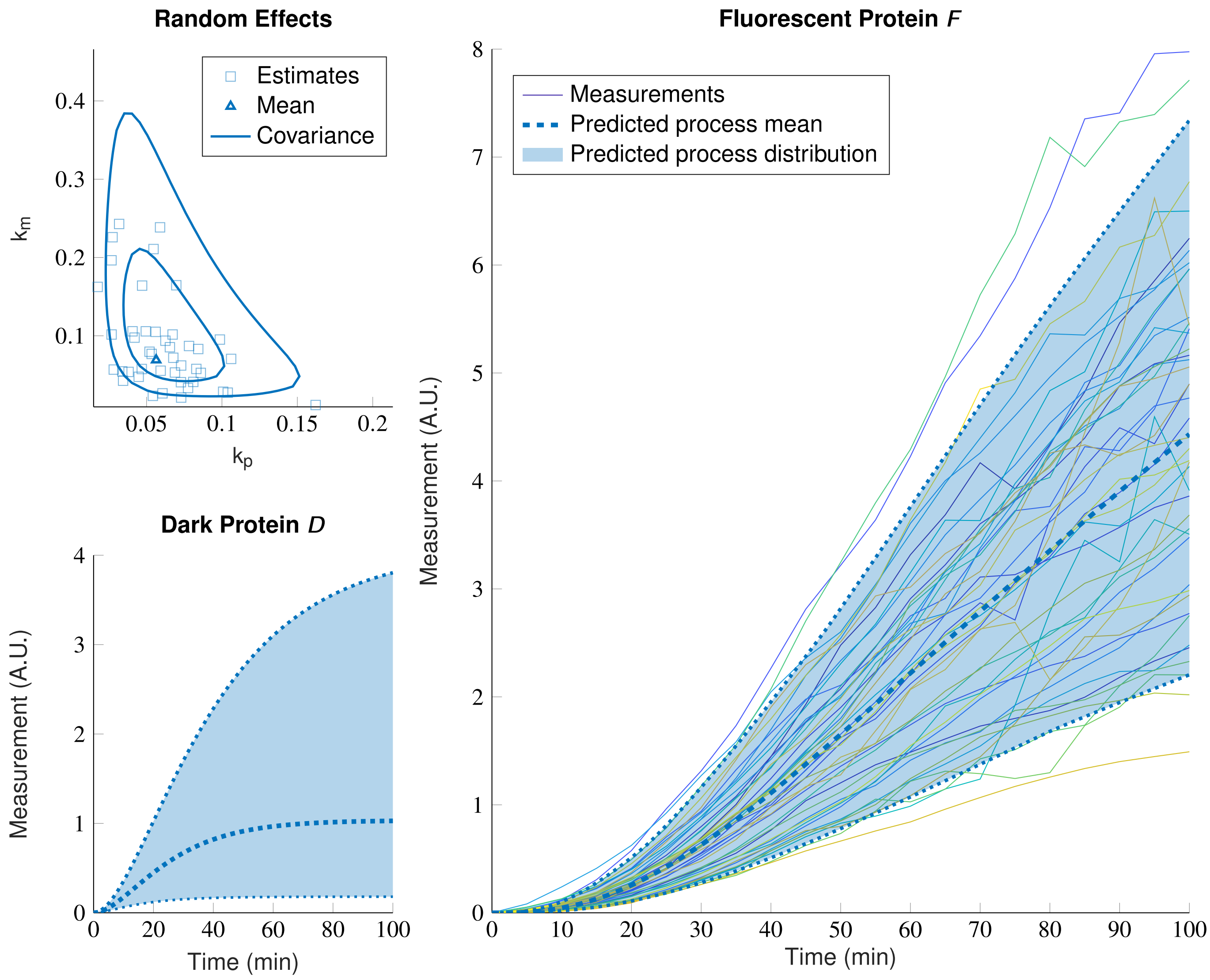

Figure S14: mCitrine: parameter inference and state predictions. *Top left panel:* individual estimates of k_p and k_m produced by the first stage of GMGTS (squares) and the associated random effects distribution obtained from the second stage, summarized by its mean and 68% and 95% contour levels. *Remaining panels:* Predicted distributions of immature and mature FP, summarized by their mean and variability (90% confidence intervals of the state distributions). The (preprocessed) experimental data are also included in the right panel.

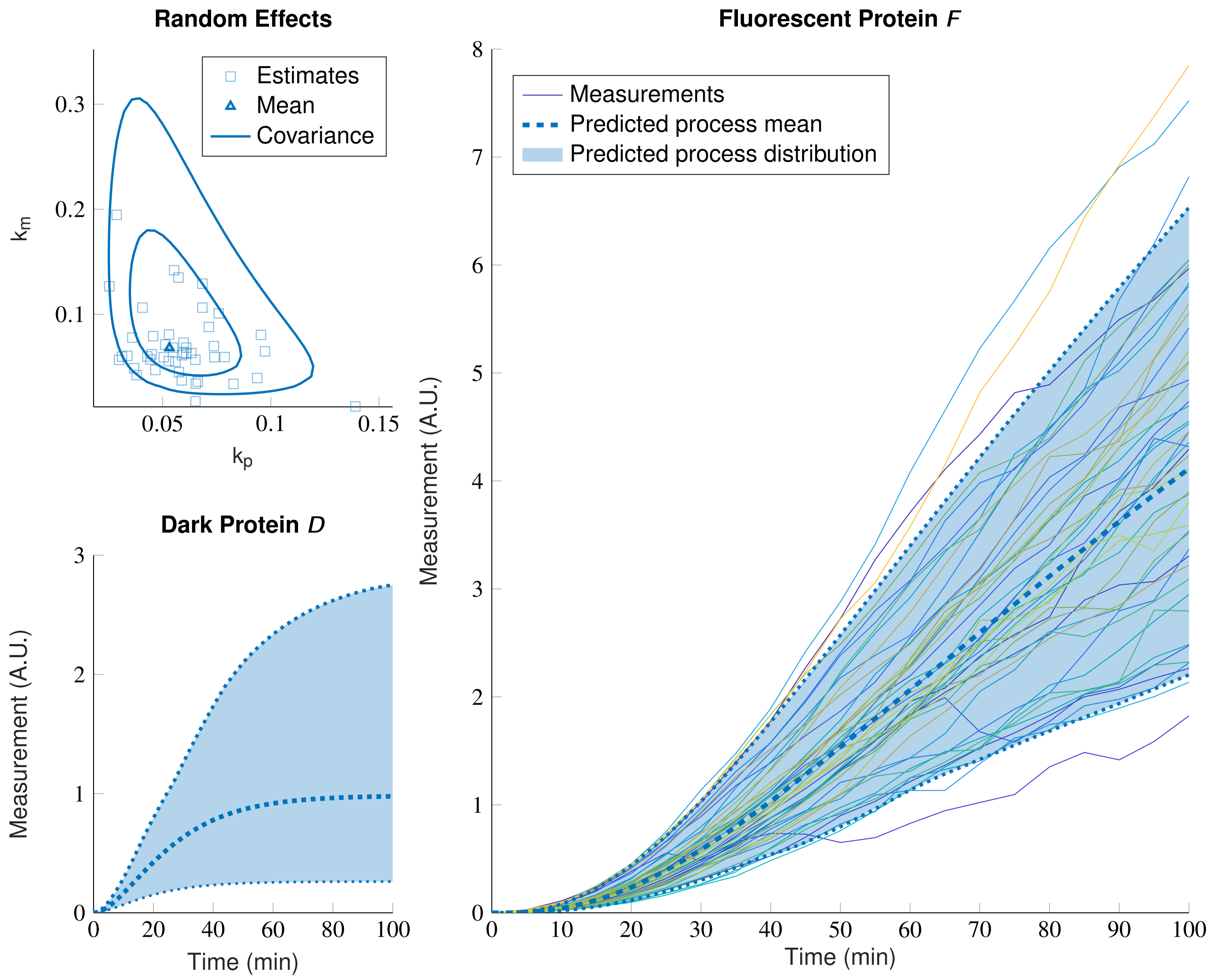

Figure S15: mVenus: parameter inference and state predictions. *Top left panel:* individual estimates of k_p and k_m produced by the first stage of GMGTS (squares) and the associated random effects distribution obtained from the second stage, summarized by its mean and 68% and 95% contour levels. *Remaining panels:* Predicted distributions of immature and mature FP, summarized by their mean and variability (90% confidence intervals of the state distributions). The (preprocessed) experimental data are also included in the right panel.

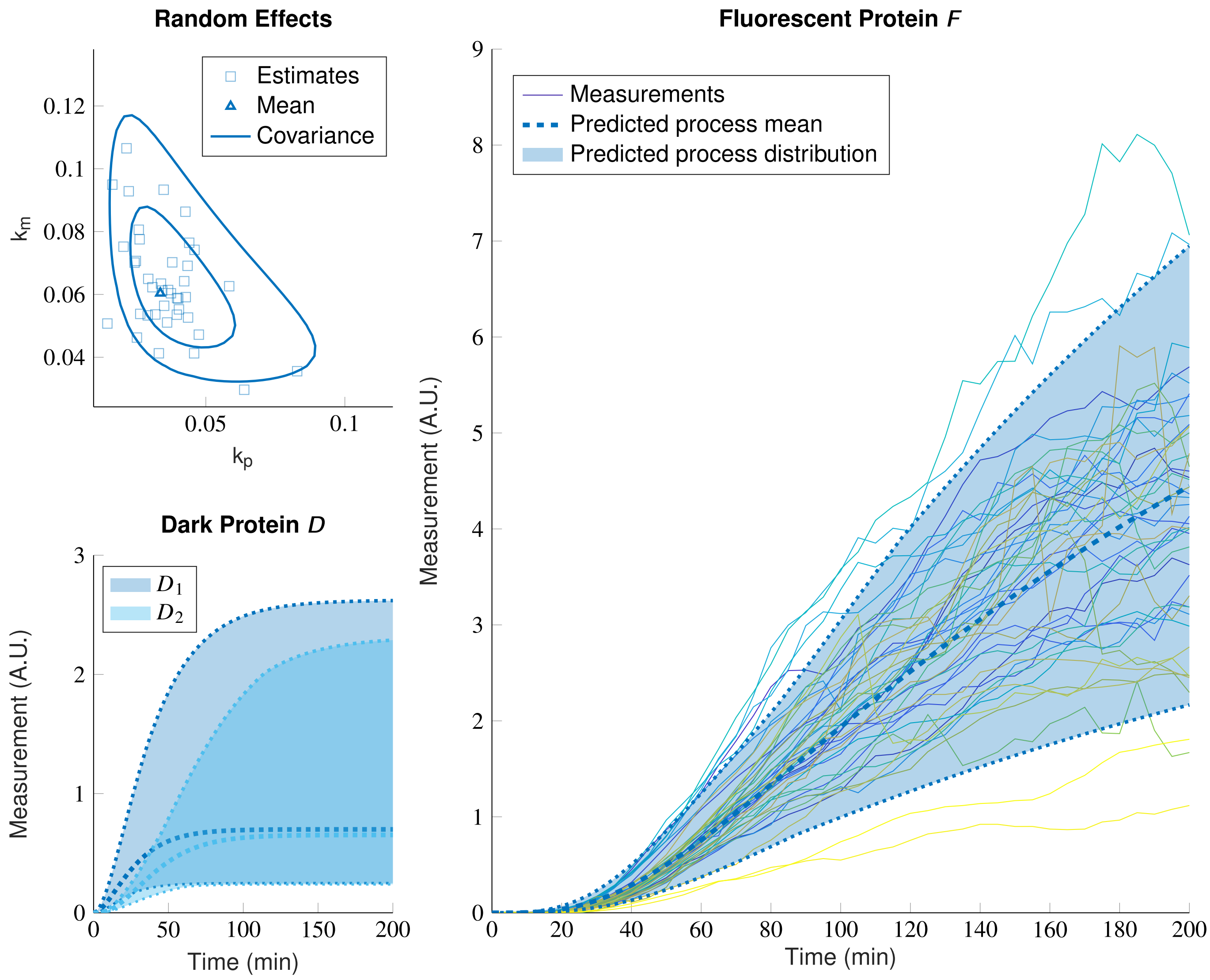

Figure S16: mScarlet-I: parameter inference and state predictions. *Top left panel:* individual estimates of k_p and k_m produced by the first stage of GMGTS (squares) and the associated random effects distribution obtained from the second stage, summarized by its mean and 68% and 95% contour levels. *Remaining panels:* Predicted distributions of immature and mature FP, summarized by their mean and variability (90% confidence intervals of the state distributions). The (preprocessed) experimental data are also included in the right panel.

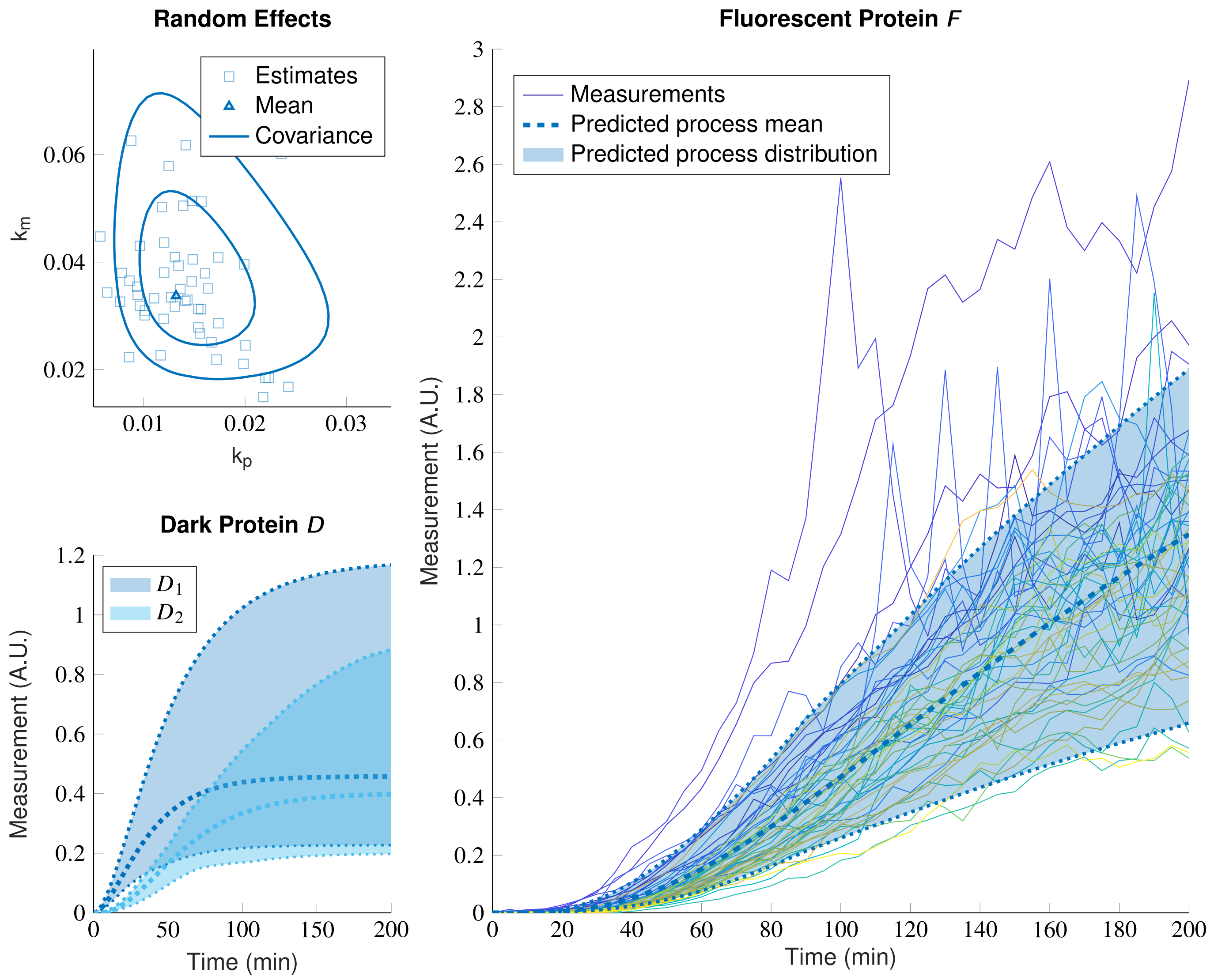

Figure S17: mCherry: parameter inference and state predictions. *Top left panel:* individual estimates of k_p and k_m produced by the first stage of GMGTS (squares) and the associated random effects distribution obtained from the second stage, summarized by its mean and 68% and 95% contour levels. *Remaining panels:* Predicted distributions of immature and mature FP, summarized by their mean and variability (90% confidence intervals of the state distributions). The (preprocessed) experimental data are also included in the right panel.

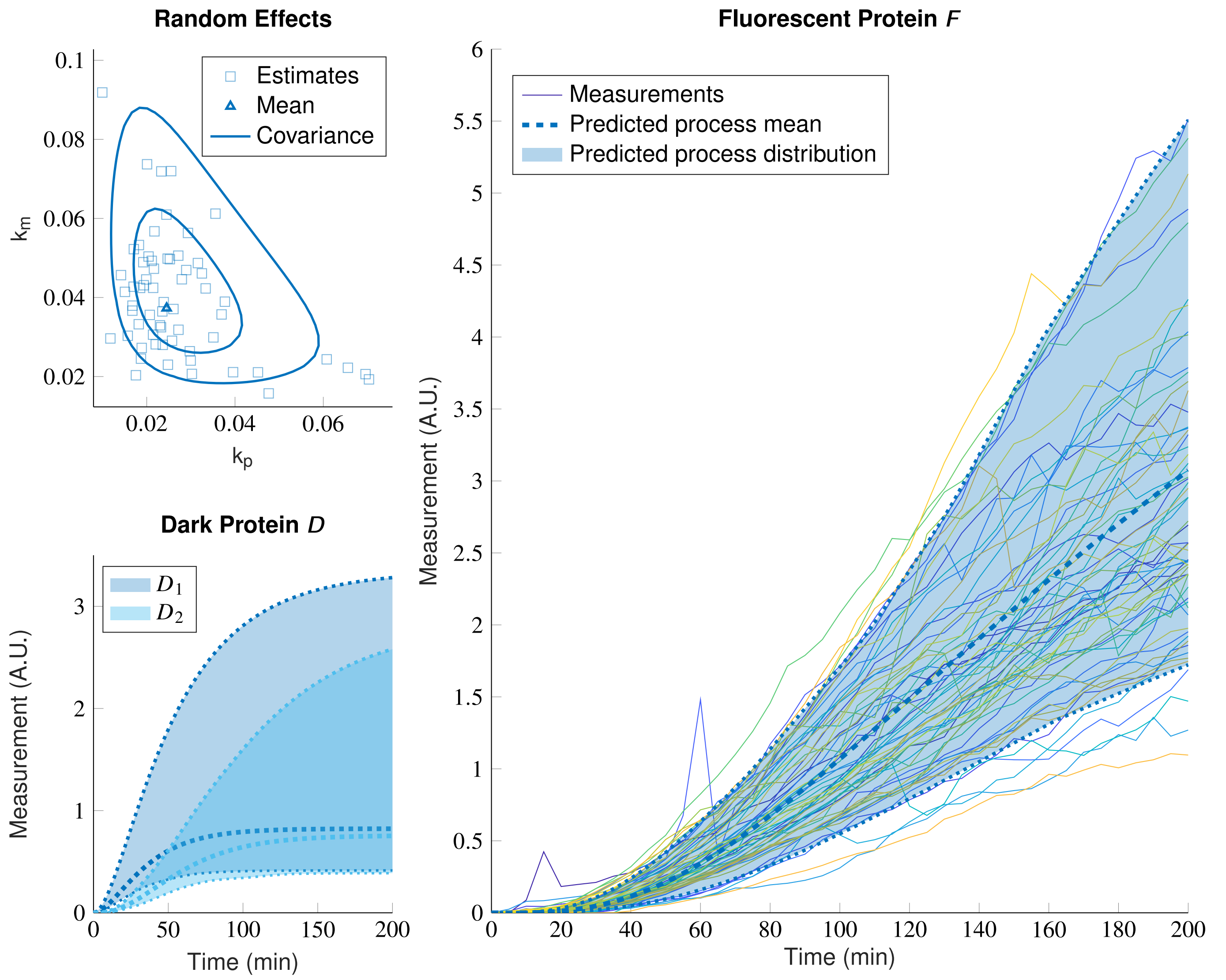

Figure S18: mTurquoise2: parameter inference and state predictions. *Top left panel:* individual estimates of k_p and k_m produced by the first stage of GMGTS (squares) and the associated random effects distribution obtained from the second stage, summarized by its mean and 68% and 95% contour levels. *Remaining panels:* Predicted distributions of immature and mature FP, summarized by their mean and variability (90% confidence intervals of the state distributions). The (preprocessed) experimental data are also included in the right panel.

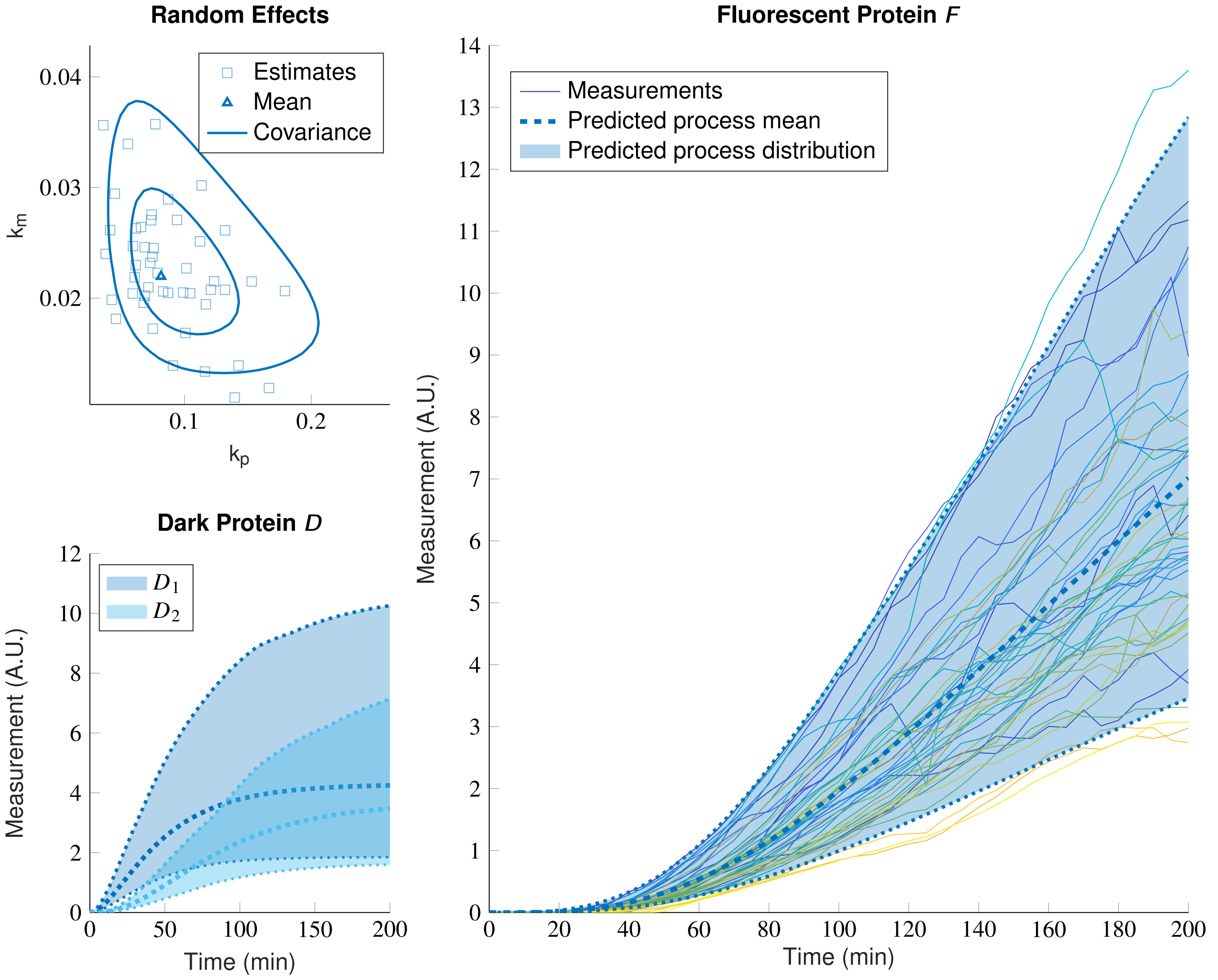

Figure S19: mTFP1: parameter inference and state predictions. *Top left panel:* individual estimates of k_p and k_m produced by the first stage of GMGTS (squares) and the associated random effects distribution obtained from the second stage, summarized by its mean and 68% and 95% contour levels. *Remaining panels:* Predicted distributions of immature and mature FP, summarized by their mean and variability (90% confidence intervals of the state distributions). The (preprocessed) experimental data are also included in the right panel.

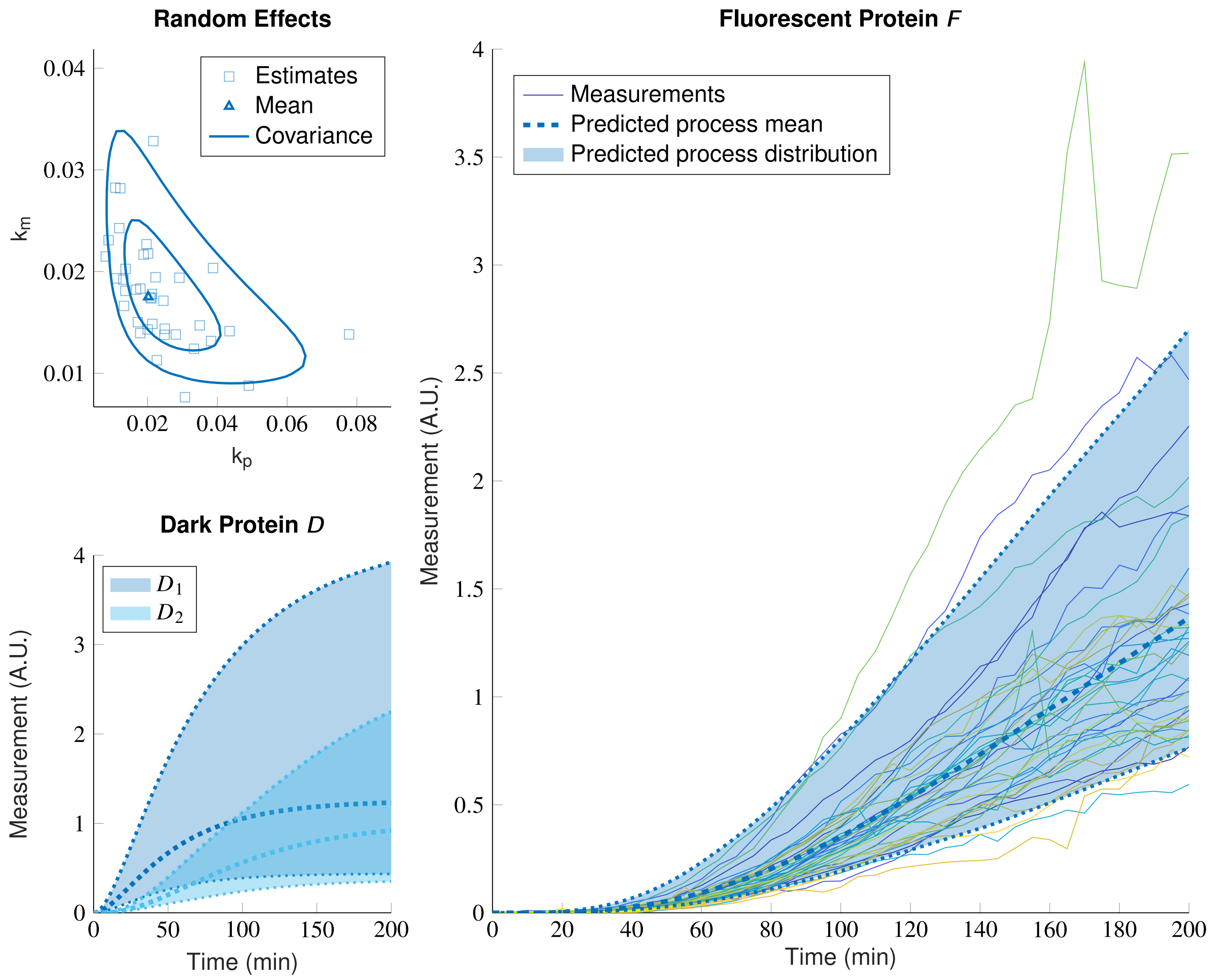

Figure S20: tdTomato: parameter inference and state predictions. *Top left panel:* individual estimates of k_p and k_m produced by the first stage of GMGTS (squares) and the associated random effects distribution obtained from the second stage, summarized by its mean and 68% and 95% contour levels. *Remaining panels:* Predicted distributions of immature and mature FP, summarized by their mean and variability (90% confidence intervals of the state distributions). The (preprocessed) experimental data are also included in the right panel.

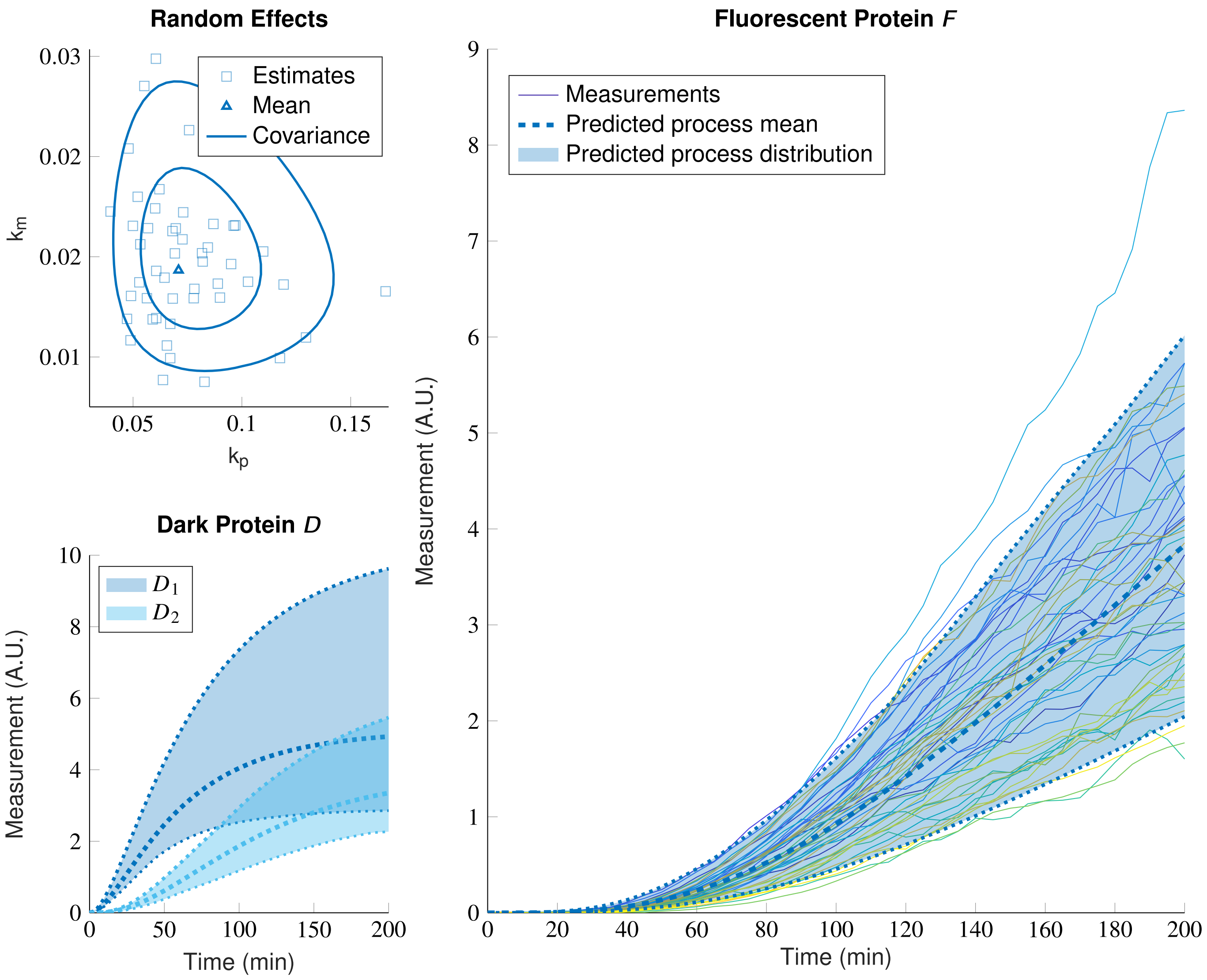

Figure S21: mKate2: parameter inference and state predictions. *Top left panel:* individual estimates of k_p and k_m produced by the first stage of GMGTS (squares) and the associated random effects distribution obtained from the second stage, summarized by its mean and 68% and 95% contour levels. *Remaining panels:* Predicted distributions of immature and mature FP, summarized by their mean and variability (90% confidence intervals of the state distributions). The (preprocessed) experimental data are also included in the right panel.

References

[Berger and Casella, 2001] Berger, R. L. and Casella, G. (2001). *Statistical Inference*. Duxbury. 4, 15

[Corless et al., 1996] Corless, R. M., Gonnet, G. H., Hare, D. E., Jeffrey, D. J., and Knuth, D. E. (1996). On the Lambert W function. *Advances in Computational mathematics*, 5:329–359. 9

[Davidian and Giltinan, 2017] Davidian, M. and Giltinan, D. M. (2017). *Nonlinear Models for Repeated Measurement Data*. Routledge. 2, 3

[Dharmarajan et al., 2019] Dharmarajan, L., Kaltenbach, H.-M., Rudolf, F., and Stelling, J. (2019). A simple and flexible computational framework for inferring sources of heterogeneity from single-cell dynamics. *Cell Systems*, 8(1):15–26. 24

[Ghosal and van der Vaart, 2017] Ghosal, S. and van der Vaart, A. W. (2017). *Fundamentals of nonparametric Bayesian inference*, volume 44. Cambridge University Press. 13

[Givens and Shortt, 1984] Givens, C. R. and Shortt, R. M. (1984). A class of Wasserstein metrics for probability distributions. *Michigan Mathematical Journal*, 31(2):231–240. 24

[Greene, 2003] Greene, W. (2003). *Econometric Analysis, 5th Ed.* Pearson. 2, 4

[Guerra et al., 2022] Guerra, P., Vuillemenot, L.-A., Rae, B., Ladyhina, V., and Milias-Argeitis, A. (2022). Systematic in vivo characterization of fluorescent protein maturation in budding yeast. *ACS Synthetic Biology*, 11(3):1129–1141. 7, 8, 9, 28, 29

[Horn and Johnson, 2012] Horn, R. A. and Johnson, C. R. (2012). *Matrix Analysis*. Cambridge University Press. 19, 23

[Kendrick, 2002] Kendrick, D. A. (2002). *Stochastic Control for Economic Models*. The University of Texas. 10

[Kurdyaeva and Milias-Argeitis, 2021] Kurdyaeva, T. and Milias-Argeitis, A. (2021). Uncertainty propagation for deterministic models of biochemical networks using moment equations and the extended kalman filter. *Journal of the Royal Society Interface*, 18(181):20210331. 6

[Petersen and Pedersen, 2008] Petersen, K. B. and Pedersen, M. S. (2008). The matrix cookbook. *Technical University of Denmark*, 7(15):510. 10, 15

[Rullan et al., 2018] Rullan, M., Benzinger, D., Schmidt, G. W., Milias-Argeitis, A., and Khammash, M. (2018). An optogenetic platform for real-time, single-cell interrogation of stochastic transcriptional regulation. *Molecular Cell*, 70(4):745–756. 8

[Sastry, 2013] Sastry, S. (2013). *Nonlinear systems: analysis, stability, and control*, volume 10. Springer Science & Business Media. 19

[Schittkowski, 2002] Schittkowski, K. (2002). *Numerical data fitting in dynamical systems: a practical introduction with applications and software*, volume 77. Kluwer Acadmic Publishers. 3

[Schmid and Biegler, 1994] Schmid, C. and Biegler, L. T. (1994). Quadratic programming methods for reduced hessian SQP. *Computers & chemical engineering*, 18(9):817–832. 23

[van Oppen, 2025] van Oppen, Y. (2025). GMGTS Matlab implementation. doi: http://doi.org/10.5281/zenodo.14884457. 21

[Varah, 1982] Varah, J. M. (1982). A spline least squares method for numerical parameter estimation in differential equations. *SIAM Journal on Scientific and Statistical Computing*, 3(1):28–46. 3

[Wu et al., 2012] Wu, H., Xue, H., and Kumar, A. (2012). Numerical discretization-based estimation methods for ordinary differential equation models via penalized spline smoothing with applications in biomedical research. *Biometrics*, 68(2):344–352. 15

[Zhou et al., 1998] Zhou, S., Shen, X., and Wolfe, D. A. (1998). Local asymptotics for regression splines and confidence regions. *The Annals of Statistics*, 26(5):1760–1782. 15
